# Association between mental health and chronic obstructive pulmonary disease (COPD) outcomes: systematic review and meta-analysis

**DOI:** 10.1136/bmjresp-2025-003977

**Published:** 2026-06-18

**Authors:** Sulaiman Alsaab, Michael Newnham, Alice M Turner, Hayyaf Aldossary, Abdullah Albukhary, Amanda Farley

**Affiliations:** 1 Department of Applied Health Research, University of Birmingham, Birmingham, UK; 2 Respiratory Care Department, Imam Abdulrahman Bin Faisal University, Dammam, Saudi Arabia; 3 Basic Medical Sciences, College of Medicine, King Saud bin Abdulaziz University for Health Sciences, Riyadh, Saudi Arabia

**Keywords:** COPD, Psychology

## Abstract

**Objective:**

To systematically synthesise evidence on the associations between presence of a mental health condition and chronic obstructive pulmonary disease (COPD) outcomes in people diagnosed with COPD, including lung function, symptom burden, functional status and clinical events.

**Methods:**

A systematic search of MEDLINE, Embase, PubMed, PsycINFO and CINAHL identified studies comparing COPD outcomes in adults (≥18 years) with and without comorbid mental illnesses. Eligible studies reported at least one relevant outcome comparing these groups. The protocol was pre-registered and followed the Preferred Reporting Items for Systematic Reviews and Meta-Analyses (PRISMA) guidelines.

**Results:**

58 studies including 707 037 participants were included. Comorbid mental illness was associated with worse outcomes: lower forced expiratory volume in 1 s (mean difference (MD)=–2.92%, 95% CI –4.35% to –1.48%), lower diffusing capacity of the lungs for carbon monoxide (MD=–3.79%, 95% CI –5.72% to –1.86%), reduced 6-minute walking test distance (MD=–30.42 m, 95% CI –41.03 to –19.82) and higher modified Medical Research Council scores (MD=0.64, 95% CI 0.45 to 0.84). Quality of life was worse (higher St George’s Respiratory Questionnaire (MD=15.23 points, 95% CI 13.25 to 17.22), COPD Assessment Test (MD=7.28, 95% CI 5.81 to 8.74)). Mental illness increased exacerbations (MD=0.87, 95% CI 0.24 to 1.50; adjusted risk ratio=1.58, 95% CI 1.05 to 2.37; incidence rate ratio (IRR)=1.64, 95% CI 1.38 to 1.96), hospitalisations (adjusted OR=1.61, 95% CI 1.43 to 1.81; adjusted IRR=2.22, 95% CI 1.30 to 3.78) and mortality (adjusted HR=1.34, 95% CI 1.07 to 1.69). Depression and anxiety showed the strongest associations; evidence for severe mental illness was limited.

**Conclusion:**

Comorbid mental illness in COPD is linked to worse lung function, greater symptom burden, higher risks of exacerbation, hospitalisation and possibly mortality. Addressing the causes of this and integrating mental healthcare into COPD management may improve outcomes and reduce preventable admissions.

**PROSPERO registration number:**

CRD42024567680.

**What is already known on this topic**

- Mental health conditions such as depression, anxiety and severe mental illness are more common among people with chronic obstructive pulmonary disease (COPD) than the general population. Some evidence shows that COPD outcomes for people with COPD and comorbid mental health conditions may be worse. Previous reviews, however, have been limited in scope and often exclude severe mental illness.

**What this study adds**

- This systematic review and meta-analysis provides a comprehensive up-to-date summary of 58 studies including over 700 000 participants and shows that comorbid mental illness is consistently associated with worse lung function, reduced exercise capacity, greater symptom burden, poorer quality of life, and higher risks of exacerbation, hospitalisation and mortality.
- Depression and anxiety demonstrated the strongest and most consistent associations, while evidence for severe mental illness remains limited but suggests similar adverse trends.
- Studies were of medium to high quality, but the majority of estimates were cross-sectional and unadjusted for confounders.

**How this study might affect research, practice or policy**

- These findings highlight inequity in COPD outcomes for people with mental health conditions and underscore the need to address the causes of this within COPD care pathways to improve outcomes and reduce preventable hospitalisations.

## Introduction

Chronic obstructive pulmonary disease (COPD) is a progressive disease characterised by chronic airflow limitation and persistent respiratory symptoms.^1^ It affects over 480 million people and has a significant economic burden due to healthcare costs and reduced healthcare workforce.^2 3^

There is increasing recognition that COPD is associated with poor mental health including depression, anxiety and severe mental illnesses (SMI).^4 5^ Depression affects up to 40% of individuals with COPD^6 7^ and anxiety disorders affect approximately one-third.^8 9^ People with anxiety and depression may experience worse COPD outcomes such as higher exacerbation rates and emergency visits^8–10^; behaviour (eg, poor engagement or adherence to treatment and higher smoking habit), increased immune response and increased systemic inflammation are potential mechanisms.^6 7^ SMIs, including schizophrenia, bipolar disorder, post-traumatic stress disorder (PTSD) and obsessive-compulsive disorder (OCD), have received comparatively limited research attention but may also be associated with worse COPD outcomes; these patients also experience higher exacerbation rates, hospitalisations and mortality.^5 11–13^

The Global Initiative for Chronic Obstructive Lung Disease recognises that anxiety and depression may be associated with COPD prognosis, hospitalisations and mortality.^1^ The National Institute for Health and Care Excellence also highlights the importance of addressing depression to improve outcomes.^14^ Previous narrative and systematic reviews summarised evidence on the association between mental health and some COPD outcomes. For example, a recent systematic review and meta-analysis investigated the association of depression and anxiety with 30-day readmission rates and acute exacerbations, finding both conditions to be significant risk factors for these outcomes. However, existing reviews often remain limited and outdated, omit various recent large-scale studies or lack a comprehensive assessment of the broader spectrum of mental health conditions, particularly SMIs.^5 6 10 11 15^

Building on these important contributions, our systematic review addresses key remaining gaps by comprehensively synthesising a larger body of existing evidence regarding the impact of depression and anxiety as well as SMI. Furthermore, our review provides a more expanded analysis of COPD outcomes, encompassing a wider range of measures such as lung function (forced expiratory volume in 1 s (FEV_1_%), diffusing capacity of the lungs for carbon monoxide (DLCO)), functional capacity (6-minute walking test (6MWT)), symptom burden (modified Medical Research Council (mMRC)), health-related quality of life (HRQoL; St George’s Respiratory Questionnaire (SGRQ), COPD Assessment Test (CAT)) and clinical events like exacerbations, 1-year hospitalisations and mortality. This broader scope aims to inform both clinical guidelines and future research priorities.

### Study design and methods

This systematic review was conducted and reported in accordance with the Preferred Reporting Items for Systematic Reviews and Meta-Analyses (PRISMA) guidelines. The protocol was pre-registered (PROSPERO: CRD42024567680).

#### Search strategy

We searched five databases (Embase, MEDLINE, PubMed, PsycINFO and CINAHL) for studies published in English between January 2004 and September 2025. Search terms included combinations of Medical Subject Headings terms and keywords related to COPD, depression, anxiety and SMI (see the online supplemental file).

10.1136/bmjresp-2025-003977.supp1 Supplementary data

#### Eligibility

Inclusion criteria were as follows: Population—adult patients with a confirmed diagnosis of COPD (FEV_1_/forced vital capacity <0.7 or diagnostic codes for studies using databases); Exposure—depression, anxiety or SMI (for included conditions see the Search strategy section); Comparator—patients with COPD without mental illnesses; Outcome—COPD exacerbations, lung function measures (FEV_1_%, DLCO), 1-year hospitalisation, mortality, symptoms and quality of life/functional capacity (SGRQ, CAT, mMRC and 6MWT); Study design—cohort, cross-sectional and case–control studies.

#### Screening and data extraction

Titles and abstracts were independently screened by two reviewers from a group of five authors (SA, AF, HA, AA, AB) using Covidence. Studies progressed to full-text screening once two independent reviewers had completed their assessment. Full-text screening was conducted independently by two reviewers from a group of three authors (SA, AA, AF).

Data extraction was performed independently by two reviewers from a group of four authors (SA, AF, HA, AA) using standardised forms within Covidence.

#### Quality assessment

Quality assessment was conducted independently by two reviewers from the same group (SA, AF, HA, AA) using the Newcastle-Ottawa Scale, adapted appropriately for cohort, case–control and cross-sectional studies (see the online supplemental file), with each study assessed by a pair of reviewers.

At all stages, Covidence ensured that each study was reviewed by two independent reviewers before progressing, and disagreements were resolved through consensus or consultation with a third reviewer (AF or MN).

Total quality scores were categorised based on predefined thresholds adapted from Zulkipli *et al*^16^: 7–9 stars as good quality, 5–6 fair and 0–4 poor. Disagreements between reviewers were resolved through consensus discussions (further details in the online supplemental file).

#### Analysis

We extracted nine outcomes grouped into three categories: (1) physiological outcomes and functional status (FEV_1_, DLCO, 6MWT), (2) symptom severity and quality of life (SGRQ, mMRC, CAT) and (3) clinical events (exacerbations, 1-year hospitalisation, mortality). Studies that presented MRC were transformed to mMRC by subtracting 1 and were combined. Exacerbations and hospitalisation were reported in different ways in included studies, preventing conversion into a single measure, and so different measurement methods were combined separately. Exacerbations were reported in four methods: (1) number of exacerbations per year, (2) odds of having at least one exacerbation during follow-up, (3) odds of being a frequent exacerbator last year (≥2/year) and (4) incidence rate ratio (IRR) during follow-up. Hospitalisation was reported as: (1) number of hospitalisations during follow-up and (2) odds of being hospitalised during follow-up. For outcomes in cohort studies, data from around 1-year follow-up (eg, hospitalisation) were extracted to reflect long-term effects. Where available, adjusted effect estimates were extracted. Adjusted data were meta-analysed separately from unadjusted data to ensure consistency across studies. All other outcomes did not require transformation or separate meta-analyses.

Exposures were categorised as: depression, anxiety, SMI (which included schizophrenia, bipolar disorder, PTSD and OCD) and mixed, referring to studies that combined multiple mental illnesses without separating their effects.

Statistical analyses were performed using Review Manager (RevMan V.7.2.0, Cochrane Collaboration). A random-effects model was applied for all meta-analyses due to expected methodological and clinical heterogeneity among included studies. Sensitivity analysis was conducted based on study quality, and subgroup analysis was done based on study design (cohort vs cross-sectional) and type of mental illness.

Publication bias was assessed using funnel plots for outcomes with ≥10 studies and visually inspected for asymmetry.

Patients and the public were not involved in the design, conduct or reporting of this research.

## Results

**Figure 1:**
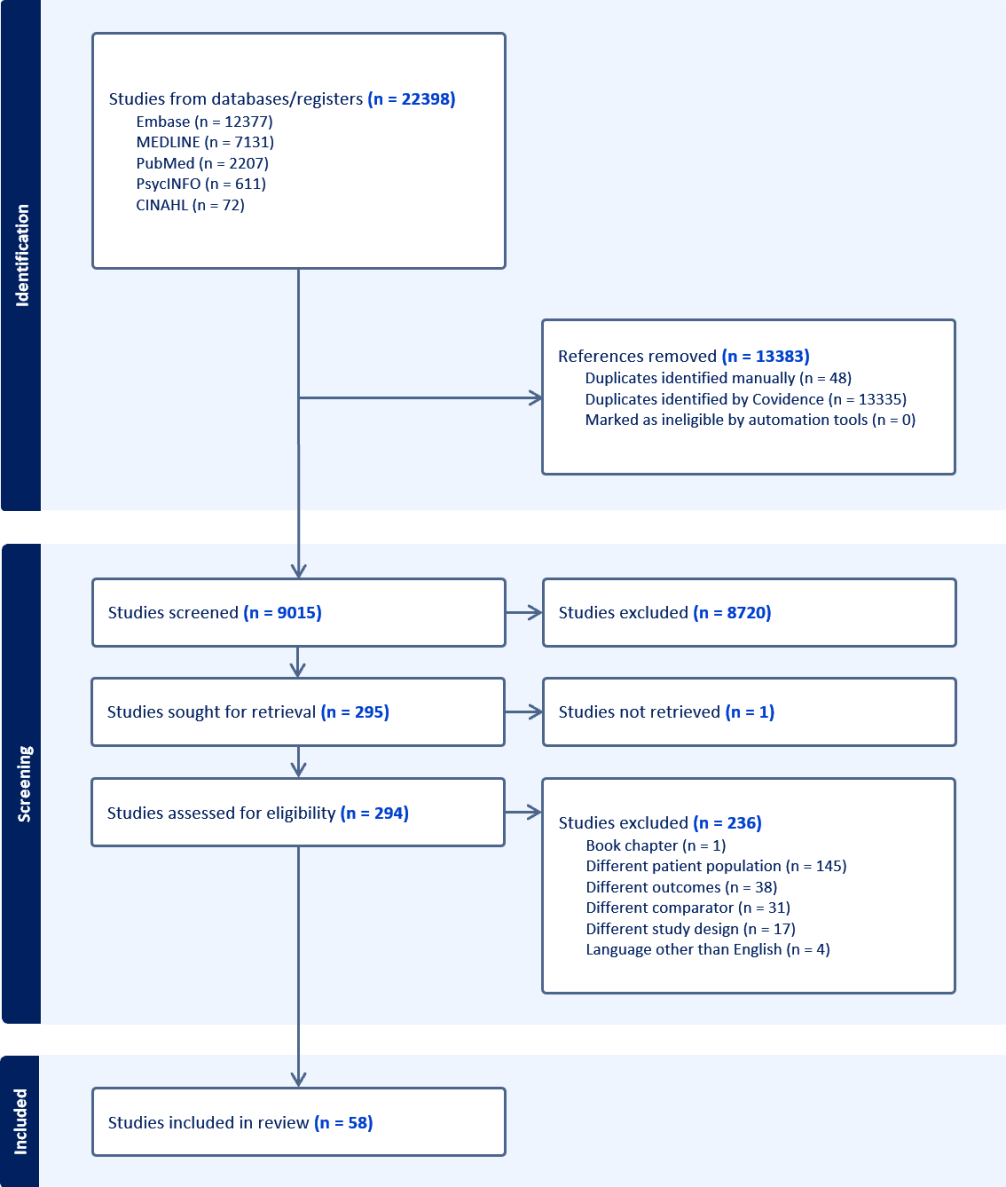
Preferred Reporting Items for Systematic Reviews and Meta-Analyses (PRISMA) flow chart of the screening process.

Searches returned 9015 unique articles, of which 294 were put forward for full-text screening and 58 were included. See the PRISMA flow chart (figure 1).

### Study characteristics

Included studies (online supplemental table 1) comprised 707 037 patients, with individual study sizes ranging from n=19 to n=382 125 participants.^7 13 17–72^ Most patients were older adults, with mean ages ranging from 58 to 77 years. Included studies were conducted worldwide across 28 counties, with the highest representation from the USA (n=12), China (n=9) and UK (n=8).^7 13 17–70 72^

All included studies had an observational design (29 cohort studies, 28 cross-sectional and one case–control); 41 reported depression, 19 anxiety, 7 SMI and 10 mental illnesses combined as one group. In most cohort studies, the relevant data were only available at baseline, resulting in cross-sectional rather than longitudinal estimates. Settings where patients were recruited varied, including inpatient, outpatient and combined inpatient/outpatient settings.

A variety of validated mental illness diagnostic tools were used, including structured clinical interviews based on Hospital Anxiety and Depression Scale (HADS), Mini International Neuropsychiatric Interview, Diagnostic and Statistical Manual of Mental Disorders, Fourth Edition (DSM-IV) or DSM-V and standardised self-reported questionnaires (online supplemental table 1).

Most studies were of fair to good quality; only eight were poor. The quality assessment can be found in online supplemental table 2.

### Physiological outcomes and functional status

#### Forced expiratory volume in 1 s

Patients with COPD with comorbid mental illness had lower FEV_1_% compared with those without (mean difference (MD)=−2.92%, 95% CI −4.35% to −1.48%, 44 studies, n=16 709), with high heterogeneity across studies (I²=86%) (online supplemental figure 1).^18 20 21 23–31 34–37 40 42 44–57 60 61 63 65 66^ Among subgroups, only depression showed a significant reduction in FEV_1_% (MD=−3.74, 95% CI −5.53 to −1.95), approaching the minimal clinically important difference (MCID) of approximately 4–10%.^73^ Anxiety (MD=−0.66, 95% CI −2.16 to 0.85) and SMI (MD=−0.94, 95% CI −8.43 to 6.54) showed no significant effect (table 1).

**Table 1:** Summary of COPD outcomes

Outcome	Subgroup effects (effect estimate (95% CI))				Pooled I² (%)
	Depression	Anxiety	SMI	All mental illnesses (pooled)	
FEV_1_%	MD −3.74 (−5.53 to −1.95)	MD −0.66 (−2.16 to 0.85)	MD −0.94 (−8.43 to 6.54)	MD −2.92 (−4.35 to −1.48)	86
DLCO%	MD −3.71 (−6.23 to −1.20)	MD −3.90 (−6.91 to −0.89)	NA	MD −3.79 (−5.72 to −1.86)	0
6MWT (m)	MD −40.13 (−46.83 to −33.42)	MD −43.77 (−60.19 to −27.36)	NA	MD −30.42 (−41.03 to −19.82)	82
SGRQ (total)	MD 15.40 (13.09 to 17.72)	MD 15.08 (10.56 to 19.61)	MD 15.46 (4.30 to 26.63)	MD 15.23 (13.25 to 17.22)	90
mMRC (point)	MD 0.83 (0.53 to 1.14)	MD 0.49 (0.39 to 0.60)	MD 0.00 (−0.37 to 0.37)	MD 0.64 (0.45 to 0.84)	89
CAT (point)	MD 7.80 (4.66 to 10.93)	MD 6.89 (5.62 to 8.16)	MD 5.80 (1.47 to 10.13)	MD 7.28 (5.81 to 8.74)	81
Exacerbations (rate)	MD 0.80 (0.13 to 1.48)	NA	MD 1.36 (0.32 to 2.40)	MD 0.87 (0.24 to 1.50)	95
Exacerbations (RR)*	RR 1.85 (0.41 to 8.38)	NA	RR 1.56 (1.02 to 2.38)	RR 1.58 (1.05 to 2.37)	0
Exacerbations (OR)*	OR 1.18 (1.07 to 1.30)	OR 4.03 (2.22 to 7.32)	NA	OR 2.10 (0.63 to 6.99)	94
Exacerbations (IRR)*	IRR 1.76 (1.40 to 2.22)	IRR 1.40 (1.03 to 1.90)	NA	IRR 1.64 (1.38 to 1.96)	9
Hospitalisation (rate)	MD 0.65 (0.08 to 1.21)	NA	NA	MD 0.65 (0.08 to 1.21)	92
Hospitalisation (OR)	OR 2.44 (1.41 to 4.22)	OR 1.17 (0.74 to 1.84)	OR 0.93 (0.41 to 2.09)	OR 1.50 (1.18 to 1.93)	96
Hospitalisation (OR)*	OR 1.74 (1.38 to 2.20)	OR 1.45 (1.30 to 1.63)	NA	OR 1.61 (1.43 to 1.81)	43
Hospitalisation (IRR)*	IRR 2.51 (1.22 to 5.17)	IRR 1.63 (0.88 to 3.02)	NA	IRR 2.22 (1.30 to 3.78)	69
Mortality	OR 4.22 (0.51 to 34.69)	OR 1.20 (0.70 to 2.06)	OR 0.95 (0.74 to 1.22)	OR 2.17 (0.77 to 6.16)	99
Mortality (OR)*	OR 0.88 (0.47 to 1.65)	OR 0.98 (0.57 to 1.69)	OR 1.25 (1.09 to 1.43)	OR 0.98 (0.75 to 1.29)	96
Mortality (HR)*	HR 1.73 (0.99 to 3.03)	HR 1.21 (1.00 to 1.47)	NA	HR 1.34 (1.07 to 1.69)	52

Effect estimates are presented as MD for continuous outcomes, rate outcomes as IRR where reported, and RR, OR or HR for dichotomous outcomes, as appropriate.

*Adjusted analysis for confounding factors.

CAT, COPD Assessment Test; COPD, chronic obstructive pulmonary disease; DLCO, diffusing capacity of the lungs for carbon monoxide; FEV_1_, forced expiratory volume in 1 s; IRR, incidence rate ratio; MD, mean difference; mMRC, modified Medical Research Council dyspnoea scale; 6MWT, 6-minute walking test ; RR, risk ratio; SGRQ, St George’s Respiratory Questionnaire; SMI, severe mental illness.

By study design, cohort studies had the same reduction direction (MD=−2.89%, 95% CI −5.05% to −0.72%, I²=88%), whereas cross-sectional studies did not (MD=−1.76%, 95% CI −3.96% to 0.43%, I²=79%). Sensitivity analyses excluding high-risk studies yielded consistent results (online supplemental table 3).

#### Diffusing capacity of the lungs for carbon monoxide

Patients with COPD with mental illnesses had lower DLCO compared with those without (MD=−3.79%, 95% CI −5.72% to −1.86%) (two studies yielding three groups, n=2315). Heterogeneity was very low (I^2^=0%)^47 67^ (online supplemental figure 2).

There were estimates available for depression and anxiety only. On subgroup analysis, reduced DLCO was found in both groups. For depression, two studies contributed data for 1288 patients (MD=−3.71%, 95% CI −6.23% to −1.20%). For anxiety, one study reported data for 1027 patients (MD=−3.90%, 95% CI −6.91% to −0.89%). None of the included studies reported the effect of SMI on DLCO (table 1).

#### 6-minute walking test

Patients with COPD with mental illnesses had lower 6MWT distances compared with those without (MD=−30.42 m, 95% CI −41.03 to −19.82, 30 studies, n=15 189, I^2^=98) (online supplemental figure 3).^17 18 20 21 27–30 35–37 42–44 46–48 51–54 56 64 66 67^ Data were available for depression and anxiety only. On subgroup analysis, the pooled MD showed a reduced 6MWT distance in the depression group (MD=−34.13 m, 95% CI −46.83 to −33.42). Heterogeneity was high (I²=91%).

For the anxiety subgroup, six studies provided data on 3633 patients. Most studies trended towards reduced 6MWT in patients with anxiety, except for one outlier study (Hong *et al*),^67^ which lacked an explanation despite showing worse outcomes for other COPD parameters in the anxiety group. After removing this outlier (online supplemental table 4), anxiety was significantly associated with lower 6MWT distance (MD=−43.77 m, 95% CI −60.19 to −27.36), and heterogeneity decreased substantially to 53%, suggesting a more consistent effect across the remaining studies. Both depression and anxiety were significantly associated with reduced functional exercise capacity in COPD, with mean reductions exceeding the MCID of 26 m for the 6MWT^73^ (table 1).

### Symptoms severity and quality of life

#### St George’s Respiratory Questionnaire

Meta-analysis of 16 972 patients across 32 studies demonstrated that patients with COPD with mental illnesses had worse HRQoL; pooled MD was 15.23 points (95% CI 13.25 to 17.22) (online supplemental figure 4).^24–30 34–36 40 41 43 44 47 50 52 54 56 64–68^

In the depression, anxiety and SMI subgroup analyses (online supplemental table 5), pooled MD=15.40 points (95% CI 13.09 to 17.72), MD=15.08 points (95% CI 10.56 to 19.61) and MD=15.46 points (95% CI 4.30 to 26.63), respectively, indicating similar trends towards worse HRQoL, all being exceeding the MCID of 4 points for SGRQ.^73^ Heterogeneity was high (I²=90%) (table 1).

#### Modified Medical Research Council

Patients with COPD with mental illnesses had higher mMRC scores (MD=0.64 points, 95% CI 0.45 to 0.84, 17 studies, n=5159) (online supplemental figure 5).^18 23 25 31 41 42 44 46–48 51 53 54 67 68^

In subgroup analyses, both depression and anxiety were associated with increased dyspnoea (online supplemental table 6). For depression, 11 studies provided data on 2340 patients. The pooled MD was 0.83 points (95% CI 0.53 to 1.14), with high heterogeneity (I²=92%). For anxiety, three studies provided data on 2222 patients. The pooled MD was 0.49 points (95% CI 0.39 to 0.60) (I²=0%). Only one study provided data on SMI, including 110 patients. This study reported no difference in mMRC scores (MD=0.00, 95% CI −0.37 to 0.37) (table 1).

#### COPD Assessment Test

Patients with COPD with mental illnesses had higher CAT scores (MD=7.28 points, 95% CI 5.81 to 8.74, 7 studies, n=3888, I^2^=81%) (online supplemental figure 6).^49–51 62 67^

In subgroup analyses, both depression and anxiety were associated with increased CAT scores; MD for depression: 7.80 points, 95% CI 4.66 to 10.93, high heterogeneity (I²=94%), MD for anxiety: 6.89 points, 95% CI 5.62 to 8.16, no heterogeneity (I²=0%) (online supplemental table 7). For the SMI subgroup, only one study provided data on 59 patients; MD was 5.80 points (95% CI 1.47 to 10.13), suggesting a potential association with increased symptom burden (table 1).

### Clinical events

#### Exacerbations

Patients with COPD with mental illnesses experienced a higher burden of exacerbations across all reported metrics. The pooled analysis of 17 studies (n=8015) showed that patients with mental illness had more frequent or severe exacerbations than those without.^18 22 23 28 31 42 45 47 49 50 54 56 61 65 67 69^

Across seven studies reporting the mean number of exacerbations per year (online supplemental figure 7a), the MD was 0.87 (95% CI 0.24 to 1.50) (I²=95%). Five studies assessing the odds of any exacerbation (vs none) (online supplemental figure 7b) showed more than double the odds among those with mental illness (OR=2.34, 95% CI 1.63 to 3.37, I²=63%). For frequent exacerbations (≥2/year), pooled data from six studies showed 87% higher odds (OR=1.87, 95% CI 1.23 to 2.82, I²=59%) (online supplemental figure 7c) (table 1).

Subgroup analyses showed higher odds of any exacerbation in depression (OR=2.23, 95% CI 1.16 to 4.31, I²=71%) and anxiety (OR=2.99, 95% CI 1.64 to 5.44, I²=67%), whereas evidence for SMI was inconclusive due to the limited number of studies; findings related to SMI should be interpreted with caution. However, for frequent exacerbations, both depression (OR=2.01, 95% CI 0.91 to 4.41) and anxiety (OR=2.36, 95% CI 0.41 to 13.43) were not significant.

**Figure 2:**
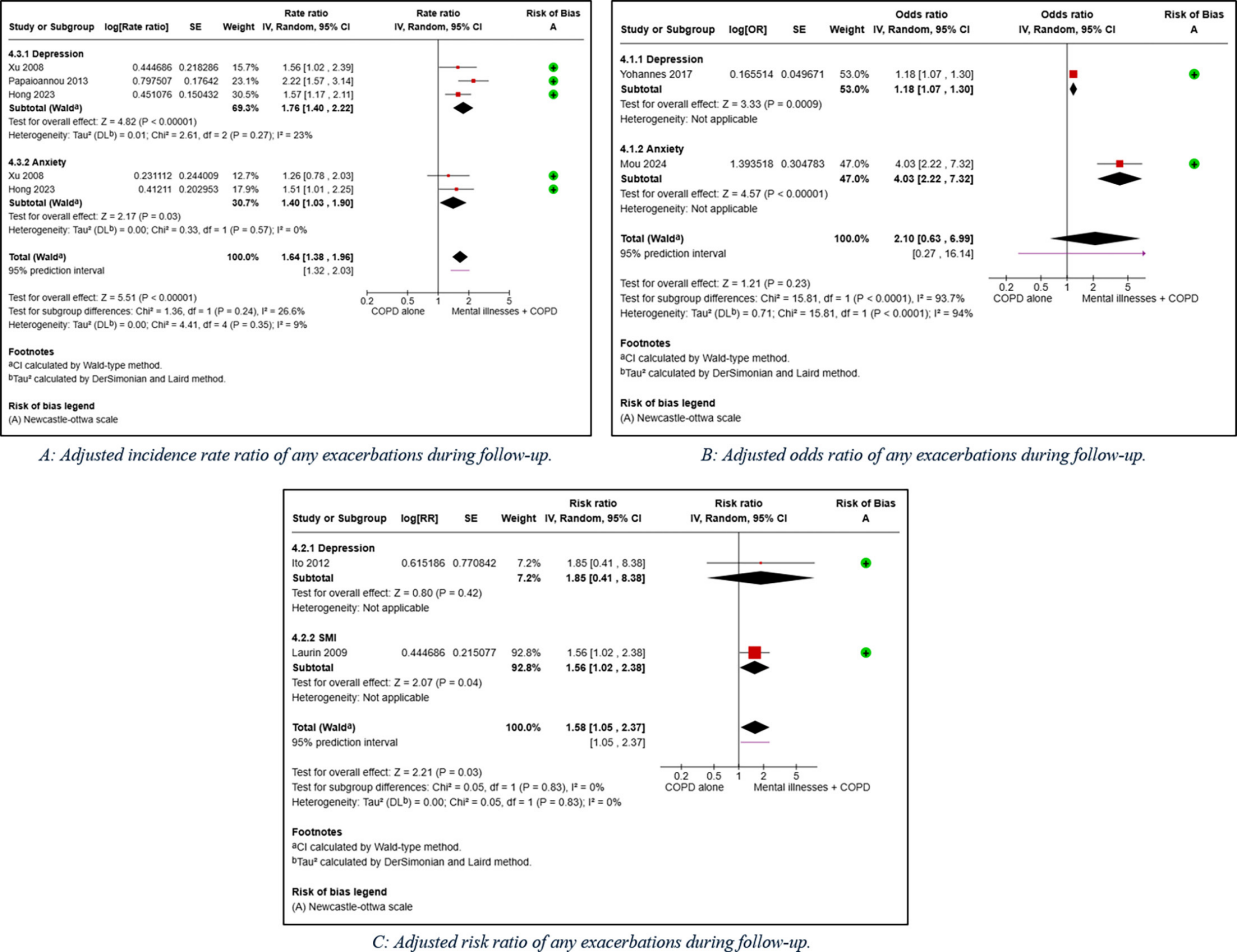
Forest plots of studies reporting exacerbations stratified by reporting method. (A) Forest plot of studies presenting adjusted incidence rate ratio of any exacerbations during follow-up for patients with COPD with and without mental illnesses (Xu *et al*^28^ adjusted for age, sex, current smoking, marital status, education level, current employment, living alone, FEV_1_%, Medical Research Council dyspnoea score, 6-minute walk distance, social support, COPD–specific self-efficacy, significant comorbidities, hospital type, use of long-acting bronchodilator and inhaled corticosteroid, and past event-based exacerbation; Papaioannou *et al*^45^ adjusted for age, sex, BMI, smoking, Charlson Comorbidity Index, GOLD stage and MRC score; Hong *et al*^67^ adjusted for age, sex, BMI, smoking history, FEV_1_ and exacerbation history). (B) Forest plot of studies presenting adjusted OR of any exacerbations during follow-up for patients with COPD with and without mental illnesses (Yohannes *et al*^56^ adjusted for age, sex, body mass index and country; Mou *et al*^22^ adjusted for age, gender, BMI, COPD course, smoking history and exacerbations in the previous year). (C) Forest plot of studies presenting adjusted risk ratio of any exacerbations during follow-up for patients with COPD with and without mental illnesses (Ito *et al*^41^ adjusted for depression, BMI, GOLD stage, requirement for LTOT and NPPV and regular use of ICS; Laurin *et al*^31^ adjusted for age, sex and length of COPD). BMI, body mass index; COPD, chronic obstructive pulmonary disease; DL, DerSimonian and Laird; FEV_1_, forced expiratory volume in 1 s; GOLD, Global Initiative for Chronic Obstructive Lung Disease; ICS, inhaled corticosteroids; IV, inverse variance; LTOT, long-term oxygen therapy; MRC, Medical Research Council; NPPV, noninvasive positive pressure ventilation; RR, risk ratio; SMI, severe mental illness.

Adjusted analyses from longitudinal cohorts confirmed these patterns. The pooled adjusted IRR (figure 2A) for exacerbations during follow-up was 1.64 (95% CI 1.38 to 1.96), with low heterogeneity (I²=9%). By subgroup, both depression (IRR=1.76, 95% CI 1.40 to 2.22, I²=23%) and anxiety (IRR=1.40, 95% CI 1.03 to 1.90, I²=0%) remained significant. Adjusted analyses based on ORs (figure 2B) and risk ratios (figure 2C) also supported elevated risk (adjusted OR=2.10, 95% CI 0.63 to 6.99; RR=1.58, 95% CI 1.05 to 2.37), though with higher heterogeneity and wide CIs.

#### One-year hospitalisation

17 studies including over 600 000 patients with COPD evaluated the association between mental illness and hospitalisation^18 19 26 28 37 39 45 49 53 54 56 58 59 61 70–72^ (table 1). Six studies reporting the number of hospitalisations (n=1719) showed a slight increase in admissions among patients with mental illness (MD=0.49, 95% CI –0.05 to 1.03, I²=91%). Excluding one study with high risk of bias made the association significant (MD=0.65, 95% CI 0.08 to 1.21) (online supplemental figure 8a).

In studies reporting hospitalisation ratios, patients with comorbid mental illness had 50% higher odds of hospital admission (OR=1.50, 95% CI 1.18 to 1.93, I²=96%) (online supplemental figure 8b). Within subgroups, depression was consistently associated with increased admissions (MD=0.65, 95% CI 0.08 to 1.21; OR=2.44, 95% CI 1.41 to 4.22), while evidence for anxiety (OR=1.17, 95% CI 0.74 to 1.84) and SMI (OR=0.93, 95% CI 0.41 to 2.09) was inconclusive. Mixed-diagnosis groups also showed higher odds (OR=2.07, 95% CI 1.26 to 3.41).

**Figure 3:**
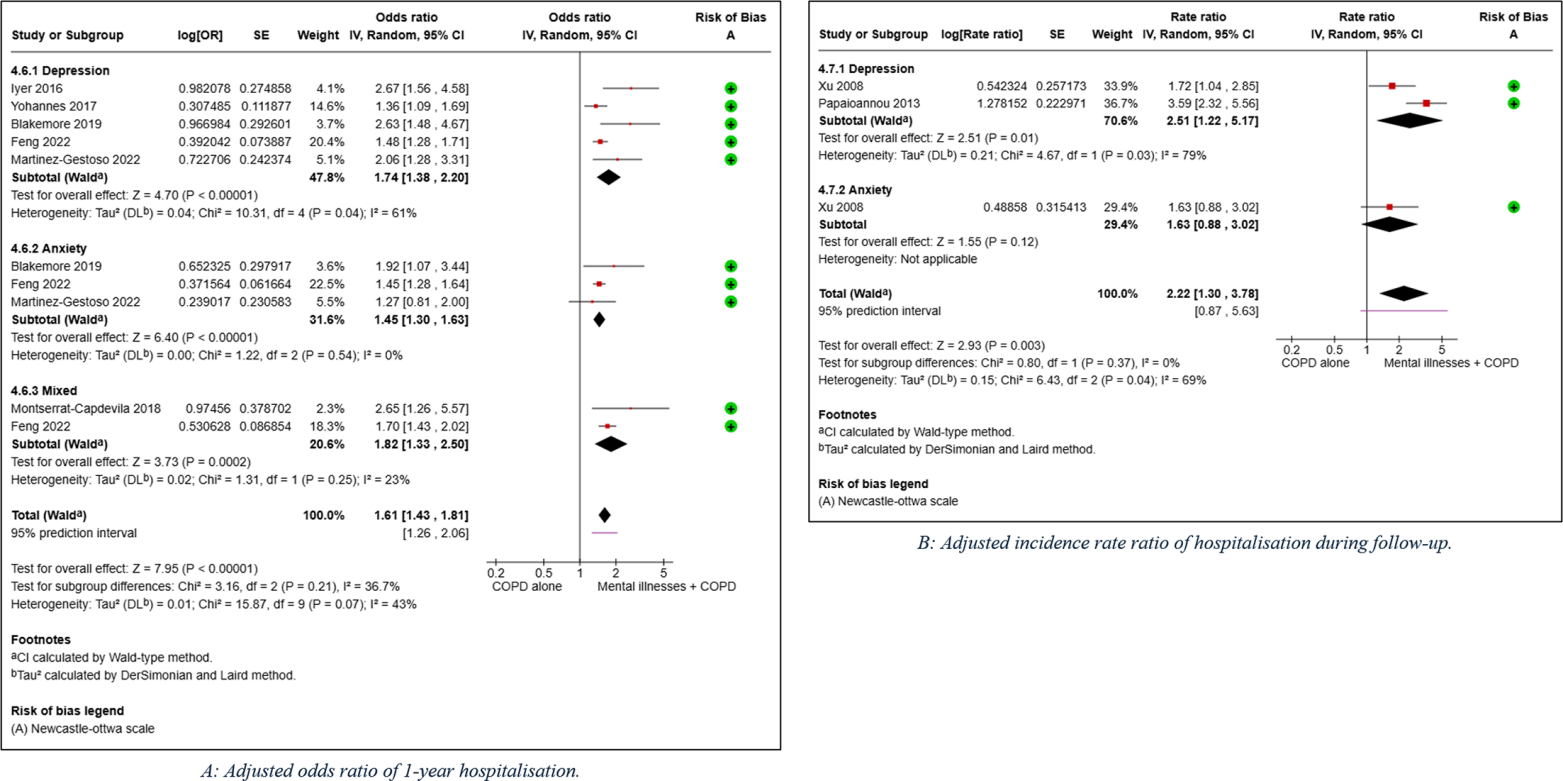
Forest plots of studies reporting hospitalisation stratified by reporting method. (A) Forest plot of studies presenting adjusted OR of 1-year hospitalisation for patients with COPD with and without mental illnesses (Iyer *et al*^70^ adjusted for age, race, sex, current smoking status, gastro-oesophageal reflux disease, depression, anxiety, serum sodium and tobacco cessation counselling; Yohannes *et al*^56^ adjusted for age, sex, body mass index and country; Blakemore *et al*^72^ adjusted for age per 10 years, number of physical diagnoses according to QOF, severity of COPD, prior use of unscheduled care in the prebaseline year and a HADS depression score of 8 or more; Feng *et al*^19^ adjusted for sex, continuous age, continuous Charlson Comorbidity Index, institute level and admission year; Martínez-Gestoso *et al*^71^ adjusted for age, gender, FEV_1_, previous year admissions and previous year emergency department). (B) Forest plot of studies presenting adjusted incidence rate ratio of hospitalisation during follow-up for patients with COPD with and without mental illnesses (Xu *et al*^28^ adjusted for age, sex, current smoking, marital status, education level, current employment, living alone, FEV_1_%, Medical Research Council dyspnoea score, 6-minute walk distance, social support, COPD–specific self-efficacy, significant comorbidities, hospital type, use of long-acting bronchodilator and inhaled corticosteroid, and past event-based exacerbation; Papaioannou *et al*^45^ adjusted for age, sex, BMI, smoking, Charlson Comorbidity Index, GOLD stage and MRC score). BMI, body mass index; COPD, chronic obstructive pulmonary disease; DL, DerSimonian and Laird; FEV_1_, forced expiratory volume in 1 s; GOLD, Global Initiative for Chronic Obstructive Lung Disease; HADS, Hospital Anxiety and Depression Scale; IV, inverse variance; MRC, Medical Research Council; QOF, Quality and Outcomes Framework.

Adjusted analyses supported these findings. The pooled adjusted OR for 1-year hospitalisation was 1.61 (95% CI 1.43 to 1.81) (I²=43%) (figure 3A), indicating a persistent association after controlling for confounders. Adjusted IRR also confirmed increased hospitalisation frequency among patients with mental illness (IRR=2.22, 95% CI 1.30 to 3.78, I²=69%) (figure 3B), particularly for depression (IRR=2.51, 95% CI 1.22 to 5.17).

#### Mortality

10 studies including over 670 000 patients with COPD evaluated the association between mental illness and mortality. The pooled unadjusted analysis showed no significant difference in mortality between patients with and without mental illness (OR=2.17, 95% CI 0.77 to 6.16, I²=99%) (online supplemental figure 9) (table 1).^13 19 26 28 39 45 58 71^

In subgroup analyses, depression was associated with higher but highly variable odds of mortality (OR=4.22, 95% CI 0.51 to 34.69, I²=99%), while anxiety (OR=1.20, 95% CI 0.70 to 2.06, I²=87%) and SMI (OR=0.95, 95% CI 0.74 to 1.22, I²=62%) showed no significant associations.

**Figure 4:**
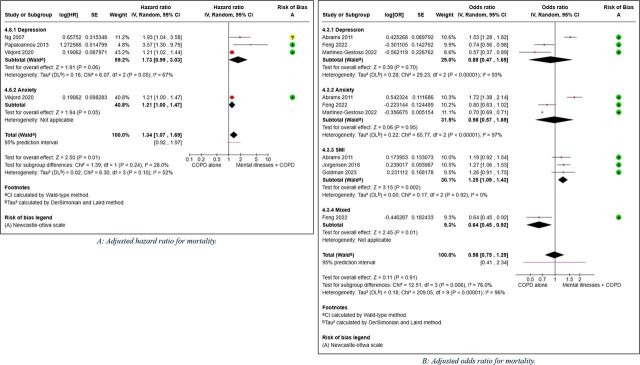
Forest plots of studies reporting mortality stratified by reporting method. (A) Forest plot of studies presenting adjusted HR for mortality for patients with COPD with and without mental illnesses (Ng *et al*^26^ adjusted for age, maternal status, length of stay, BMI, FEV_1_, FVC, SaO_2_, PaCO_2_, duration of COPD, degree of dyspnoea and smoking; Papaioannou *et al*^45^ adjusted for age, sex, BMI, smoking, Charlson Comorbidity Index, GOLD stage and MRC score; Vikjord *et al*^63^ adjusted for sex, continuous age, COPD severity, BMI, physical activity and level of education). (B) Forest plot of studies presenting adjusted OR for mortality for patients with COPD with and without mental illnesses (Abrams *et al*^39^ adjusted for age, sex, race, comorbidities, laboratory values and smoking; Feng *et al*^19^ adjusted for sex, continuous age, continuous Charlson Comorbidity Index, institute level and admission year; Martínez-Gestoso *et al*^71^ adjusted for age, gender, FEV_1_, previous year admissions and previous year emergency department; Jørgensen *et al*^58^ adjusted for age, sex and Charlson Comorbidity Index; Goldman *et al*^13^ adjusted for age, sex, deprivation, comorbidities and elective versus emergency admission). BMI, body mass index; COPD, chronic obstructive pulmonary disease; DL, DerSimonian and Laird; FEV_1_, forced expiratory volume in 1 s; FVC, forced vital capacity; GOLD, Global Initiative for Chronic Obstructive Lung Disease; IV, inverse variance; MRC, Medical Research Council; SMI, severe mental illness.

Adjusted HR for mortality was 1.34 (95% CI 1.07 to 1.69) (I²=52%) (figure 4A), showing an increase in mortality risk among patients with COPD with mental illness. Subgroup analyses showed a trend towards increased mortality in depression (HR=1.73, 95% CI 0.99 to 3.03, I²=67%) and anxiety (HR=1.21, 95% CI 1.00 to 1.47). Adjusted ORs showed no overall association (OR=0.98, 95% CI 0.75 to 1.29, I²=96%) (figure 4B), except for SMI that showed an increased mortality odds (OR=1.25, 95% CI 1.09 to 1.43).

#### Publication bias

Funnel plots for FEV_1_%, SGRQ, mMRC and hospitalisation appeared symmetrical, indicating low likelihood of publication bias. Plots for 6MWT and mortality showed asymmetry, suggesting possible publication bias or outcome reporting differences (see online supplemental figures 10–17).

## Discussion

This systematic review and meta-analysis shows an association between the presence of a comorbid mental health condition and worse COPD outcomes in people diagnosed with COPD. Our findings confirm that mental health conditions are associated with an increased burden of COPD exacerbations, 1-year hospitalisation and potential mortality. Beyond these clinical events, our analysis further reveals associations with lower FEV_1_%, DLCO, reduced 6MWT and worse quality of life and symptom burden (SGRQ, mMRC and CAT).

These associations largely remained after removal of low-quality studies in sensitivity analyses and remained regardless of the study design. In subgroup analysis, both depression and anxiety were associated with worse COPD outcomes, with depression and anxiety as the most consistently linked mental illnesses. Notably, the association between anxiety and reduced DLCO requires cautious interpretation since it was based on a single study, and although anxiety was associated with worse FEV_1_%, the result was not statistically significant. Furthermore, our review extends the analysis to include the association between mental health conditions and mortality in patients with COPD. While our meta-analysis of four studies showed a significant association between mental illness and mortality (HR=1.34, 95% CI 1.07 to 1.69), the other included studies reporting adjusted ORs were inconclusive (OR=0.98, 95% CI 0.75 to 1.29), except for those on SMI (OR=1.25, 95% CI 1.09 to 1.43). These findings warrant cautious interpretation and highlight the need for further research in this area.

Guidelines advocate for the identification and treatment of anxiety and depression in patients with COPD, and this is important given the high burden of these conditions in the COPD population.^1^ It has been hypothesised that the relationship between anxiety, depression and COPD outcomes may be bidirectional, where decline in physical health could lead to depression and anxiety symptoms and vice versa.^6^ This comprehensive systematic review searched for evidence for mental health conditions leading to worse outcomes but was not able to strongly confirm this, as available estimates were largely cross-sectional, either from cross-sectional studies or associations extracted from baseline data in cohort studies. It was also not possible to draw conclusions about causality because most studies did not adjust for potential confounders. Of particular note, smoking is more common in people with mental health conditions, and smoking is also associated with worse COPD outcomes.^74–76^ However, in the small number of studies that provided adjusted outcomes (including those that adjusted for smoking), the relationship between mental health conditions and outcomes largely remained, suggesting that these factors alone do not explain the association.

Our review also included studies in people with SMI, and we found some evidence of worse COPD outcomes in this population. However, there were relatively few studies, and similar to anxiety and depression, the estimates were mainly cross-sectional and unadjusted. Patients with SMI present unique challenges due to social isolation, medication adherence difficulties and reduced healthcare access, potentially leading to disproportionately poor COPD outcomes.^77^ However, data gaps and methodological heterogeneity limit definitive conclusions. Variations in definitions and severity assessments of SMI across studies also likely contributed to these mixed findings.^5 13^

There are several strengths to this review. We conducted a systematic and comprehensive search, and all studies were screened by two independent reviewers. The scope covers all major categories of mental illness and, for the first time, includes SMI. We also included all main relevant COPD outcomes. However, this review has several limitations. Most included studies were observational, predominantly cross-sectional and often reported unadjusted estimates, which restricts the ability to draw definitive causal conclusions. Additionally, considerable heterogeneity was observed across outcomes, reflected by high I² values. We could not fully account for the heterogeneity, but it is possible that this arises from variability in methodological quality, exposure measurement, diversity in patient populations (data were from 28 countries), publication bias or lack of control for confounding.

Building on the current evidence base, future research employing large-scale, longitudinal datasets with standardised psychiatric assessments and adjustment for confounding variables is needed to further clarify the direction and causal nature of the associations between mental health and COPD outcomes. If mental health is causally linked to worse COPD outcomes, identifying and treating mental health conditions may potentially improve respiratory outcomes. Validated tools such as HADS scale and Primary Care Evaluation of Mental Disorders could be integrated into standard COPD care to facilitate early detection and intervention using cognitive–behavioural therapy or pulmonary rehabilitation programmes incorporating psychological support and smoking cessation.^1^ These have demonstrable efficacy in improving both mental health symptoms and COPD outcomes.^78–80^ The best approach for SMI might differ from depression and anxiety due to management within secondary care, high smoking rates and failure to report physical symptoms.^77^ In such cases, proactive screening of patients with SMI for COPD and referral for COPD treatment may be a better strategy. However, more research is needed to understand mechanisms by which mental health conditions (depression, anxiety and also SMI) may lead to worse outcomes in order to identify the most effective intervention pathways to tackle the causes of inequality in outcomes.

## Conclusion

This systematic review and meta-analysis has found consistent evidence of an association between presence of a mental health condition and worse outcomes in patients with COPD. Depression and anxiety are associated with worse lung function, exercise capacity, symptoms and quality of life, as well as a higher risk of exacerbations and 1-year hospitalisation, although the association with mortality remains uncertain. Estimates are largely cross-sectional and unadjusted; so conducting large longitudinal studies that adjust for confounders and addressing the research gap surrounding SMI could further enhance our understanding and improve comprehensive care strategies for patients with COPD with comorbid mental health conditions.

## Data Availability

All data relevant to the study are included in the article or uploaded as supplementary information.
