# Supplementary material for: Association between mental health and chronic obstructive pulmonary disease (COPD) outcomes: systematic review and meta-analysis
Source: BMJ Open Respir Res. 2026 Jun 18;13(1):e003977. doi: 10.1136/bmjresp-2025-003977 (PMC13295998; doi:10.1136/bmjresp-2025-003977)
Supplement: Supplementary data [file bmjresp-13-1-s001.pdf]

Supplementary material

Contents

Search strategies: 2

Additional methods: 14

Newcastle-Ottwa scale (NOS) risk of bias assessment templates 15

Cohort studies 15

Case-control studies 17

Cross-sectional studies 19

Additional results: 21

NOS risk of bias assessment table of included studies 27

Physiological outcomes and functional status 29

FEV_1% 29

6MWT 30

Symptoms severity and quality of life: 31

SGRQ 31

mMRC 32

CAT 33

Additional figures 35

References: 55

Alsaab S, *et al. BMJ Open Respir Res* 2026; 13:e003977. doi: 10.1136/bmjresp-2025-003977

Search strategies:

Database:
MEDLINE

1	exp Pulmonary Disease, Chronic Obstructive/
2	exp Lung Diseases, Obstructive/
3	exp Bronchitis, Chronic/
4	exp Emphysema/
5	exp Pulmonary Emphysema/
6	Chronic Obstructive Pulmonary Disease.mp.
7	Chronic Obstructive Lung Disease.mp.
8	COPD.mp.
9	Chronic Obstructive Airway Disease.mp.
10	Chronic airflow obstruction.mp.
11	Chronic bronchitis.mp.
12	Emphysema.mp.
13	Pulmonary emphysema.mp.
14	Airflow limitation.mp.
15	1 or 2 or 3 or 4 or 5 or 6 or 7 or 8 or 9 or 10 or 11 or 12 or 13 or 14
16	exp Mental Disorders/
17	exp Psychiatry/
18	exp Anxiety/
19	exp Stress, Psychological/
20	exp Depressive Disorder/
21	exp Depression/
22	exp Mood Disorders/
23	exp Anxiety Disorders/
24	exp Psychotic Disorders/
25	Mental illness.mp.
26	Mental health disorder.mp.
27	Psychiatric disorder.mp.
28	Psychological disorder.mp.
29	Emotional disorder.mp.
30	Mental health*.mp.
31	Anxiety.mp.
32	exp Schizophrenia/
33	exp Bipolar Disorder/
34	exp Depressive Disorder, Major/
35	Severe mental illness.mp.
36	Serious mental illness.mp.
37	Severe psychiatric disorder.mp.
38	Serious psychiatric disorder.mp.

Alsaab S, *et al. BMJ Open Respir Res* 2026; 13:e003977. doi: 10.1136/bmjresp-2025-003977

39	Severe psychological disorder.mp.
40	Serious psychological disorder.mp.
41	Schizophrenia.mp.
42	Bipolar disorder.mp.
43	Major depressive disorder.mp.
44	Severe anxiety disorder.mp.
45	Panic*.mp.
46	16 or 17 or 18 or 19 or 20 or 21 or 22 or 23 or 24 or 25 or 26 or 27 or 28 or 29 or 30 or 31 or 32 or 33 or 34 or 35 or 36 or 37 or 38 or 39 or 40 or 41 or 42 or 43 or 44 or 45
47	exp "Quality of Life"/
48	exp Forced Expiratory Volume/
49	exacerbation*.mp.
50	quality of life.mp.
51	quality*.mp.
52	FEV1.mp.
53	forced expiratory*.mp.
54	DLCO.mp.
55	diffus*.mp.
56	TLCO.mp.
57	hospital admission.mp.
58	Mortality.mp.
59	Death.mp.
60	MRC.mp.
61	breathlessness.mp.
62	exp Dyspnea/
63	dyspnea.mp.
64	47 or 48 or 49 or 50 or 51 or 52 or 53 or 54 or 55 or 56 or 57 or 58 or 59 or 60 or 61 or 62 or 63
65	15 and 46 and 64
66	limit 65 to (english language and humans)
67	limit 66 to yr="2004 -Current"

Alsaab S, *et al. BMJ Open Respir Res* 2026; 13:e003977. doi: 10.1136/bmjresp-2025-003977

Database:
Embase

1	exp *chronic obstructive lung disease/
2	exp *obstructive lung disease/
3	exp *chronic bronchitis/
4	exp *emphysema/
5	exp *lung emphysema/
6	Chronic Obstructive Pulmonary Disease.mp.
7	Chronic Obstructive Lung Disease.mp.
8	COPD.mp.
9	Chronic bronchitis.mp.
10	Emphysema.mp.
11	Pulmonary emphysema.mp.
12	1 or 2 or 3 or 4 or 5 or 6 or 7 or 8 or 9 or 10 or 11
13	exp *mental disease/
14	exp *psychiatry/
15	exp *anxiety/
16	exp *mental stress/
17	exp *depression/
18	exp *anxiety disorder/
19	Mental illness.mp.
20	Mental health disorder.mp.
21	Psychiatric disorder.mp.
22	Psychological disorder.mp.
23	Mental health*.mp.
24	Anxiety.mp.
25	exp *schizophrenia/
26	exp *bipolar disorder/
27	exp *major depression/
28	Severe mental illness.mp.
29	Serious mental illness.mp.
30	Schizophrenia.mp.
31	Bipolar disorder.mp.
32	Major depressive disorder.mp.
33	Severe anxiety disorder.mp.
34	13 or 14 or 15 or 16 or 17 or 18 or 19 or 20 or 21 or 22 or 23 or 24 or 25 or 26 or 27 or 28 or 29 or 30 or 31 or 32 or 33
35	exp *"quality of life"/
36	exp *forced expiratory volume/
37	exacerbation*.mp.
38	quality of life.mp.

Alsaab S, *et al. BMJ Open Respir Res* 2026; 13:e003977. doi: 10.1136/bmjresp-2025-003977

39	FEV1.mp.
40	DLCO.mp.
41	TLCO.mp.
42	hospital admission.mp.
43	Mortality.mp. or exp *mortality/
44	Death.mp.
45	MRC.mp.
46	breathlessness.mp.
47	exp *dyspnea/ or Dyspnea.mp.
48	35 or 36 or 37 or 38 or 39 or 40 or 41 or 42 or 43 or 44 or 45 or 46 or 47
49	12 and 34 and 48
50	limit 49 to (human and english language and (article-in-press status or embase status) and yr="2004 -Current")

Database:
CINAHL

1	(MH “Pulmonary Diseases, Chronic Obstructive+”)
2	(MH “Lung Diseases, Obstructive+”)
3	MH “ Bronchitis, Chronic+”
4	(MH “Pulmonary Emphysema+”)
5	(MH “Emphysema+”)
6	“Chronic Obstructive Pulmonary Disease”
7	“Chronic Obstructive Lung Disease”
8	COPD
9	“Chronic Obstructive Airway Disease”
10	“Chronic airflow obstruction”
11	“Chronic bronchitis”
12	Emphysema
13	“Pulmonary emphysema”
14	“Airflow limitation”
15	1 OR 2 OR 3 OR 4 OR 5 OR 6 OR 7 OR 8 OR 9 10 OR 11 OR 12 OR 13 OR 14
16	(MH “Mental Disorders+”)
17	(MH “Psychiatry+”)
18	(MH “Anxiety+”)
19	(MH “Stress, Psychological+”)
20	(MH “Depressive Disorders+”)
21	(MH “Depression+”)
22	(MH “Mood Disorders+”)
23	(MH “Anxiety Disorders+”)
24	(MH “Psychotic Disorders+”)
25	“Mental illness”
26	“Mental health disorder”
27	“Psychiatric disorder”
28	“Psychological disorder”
29	“Emotional disorder”
30	“Mental health*”
31	Anxiety
32	(MH “Schizophrenia+”)
33	(MH “Bipolar Disorder+”)
34	(MH “Depressive Disorder, Major+”)
35	“Severe mental illness”
36	“Serious mental illness”
37	“Severe psychiatric disorder”
38	“Serious psychiatric disorder”

Alsaab S, *et al. BMJ Open Respir Res* 2026; 13:e003977. doi: 10.1136/bmjresp-2025-003977

39	"Severe psychological disorder"
40	"Serious psychological disorder"
41	Schizophrenia
42	"Bipolar Disorder"
43	"Major depressive disorder"
44	"Severe anxiety disorder"
45	Panic*
46	16 OR 17 OR 18 OR 19 OR 20 OR 21 OR 22 OR 23 OR 23 OR 24 OR 25 OR 26 OR 27 OR 28 OR 29 OR 30 OR 31 OR 32 OR 33 OR 34 OR 35 OR 36 OR 37 OR 38 OR 39 OR 40 OR 41 OR 42 OR 43 OR 44 OR 45
47	(MH "Quality of Life+")
48	(MH "Forced Expiratory Volume+")
49	Exacerbation*
50	"Quality of life"
51	Quality*
52	FEV1
53	"Forced expiratory*"
54	DLCO
55	Diffus*
56	TLCO
57	"Hospital admission"
58	Mortality
59	Death
60	MRC
61	Breathlessness
62	(MH "Dyspnea+")
63	Dyspnea
64	47 OR 48 OR 49 OR 50 OR 51 OR 52 OR 53 OR 54 OR 55 OR 56 OR 57 OR 58 OR 59 OR 60 OR 61 OR 62 OR 63
65	15 AND 46 AND 64

Database:

Psychinfo

1	exp *chronic obstructive lung disease/
2	exp *obstructive lung disease/
3	exp *chronic bronchitis/
4	exp *emphysema/
5	exp *lung emphysema/
6	Chronic Obstructive Pulmonary Disease.mp.
7	Chronic Obstructive Lung Disease.mp.
8	COPD.mp.
9	Chronic bronchitis.mp.
10	Emphysema.mp.
11	Pulmonary emphysema.mp.
12	1 or 2 or 3 or 4 or 5 or 6 or 7 or 8 or 9 or 10 or 11
13	exp *mental disease/
14	exp *psychiatry/
15	exp *anxiety/
16	exp *mental stress/
17	exp *depression/
18	exp *anxiety disorder/
19	Mental illness.mp.
20	Mental health disorder.mp.
21	Psychiatric disorder.mp.
22	Psychological disorder.mp.
23	Mental health*.mp.
24	Anxiety.mp.
25	exp *schizophrenia/
26	exp *bipolar disorder/
27	exp *major depression/
28	Severe mental illness.mp.
29	Serious mental illness.mp.
30	Schizophrenia.mp.
31	Bipolar disorder.mp.
32	Major depressive disorder.mp.
33	Severe anxiety disorder.mp.
34	13 or 14 or 15 or 16 or 17 or 18 or 19 or 20 or 21 or 22 or 23 or 24 or 25 or 26 or 27 or 28 or 29 or 30 or 31 or 32 or 33
35	exp *"quality of life"/
36	exp *forced expiratory volume/

37	exacerbation*.mp.
38	quality of life.mp.
39	FEV1.mp.
40	DLCO.mp.
41	TLCO.mp.
42	hospital admission.mp.
43	Mortality.mp. or exp *mortality/
44	Death.mp.
45	MRC.mp.
46	breathlessness.mp.
47	exp *dyspnea/ or Dyspnea.mp.
48	35 or 36 or 37 or 38 or 39 or 40 or 41 or 42 or 43 or 44 or 45 or 46 or 47
49	12 and 34 and 48
50	limit 49 to (human and english language and (article-in-press status or embase status) and yr="2004 -Current")

Alsaab S, *et al. BMJ Open Respir Res* 2026; 13:e003977. doi: 10.1136/bmjresp-2025-003977

Database:

PubMed

Search number	Search Details
**72**	("pulmonary disease, chronic obstructive"[MeSH Terms] OR "lung diseases, obstructive"[MeSH Terms] OR "bronchitis, chronic"[MeSH Terms] OR ("pulmonary emphysema"[MeSH Terms] OR "Emphysema"[MeSH Terms]) OR "chronic obstructive pulmonary disease"[Title/Abstract] OR "chronic obstructive lung disease"[Title/Abstract] OR "COPD"[Title/Abstract] OR "chronic obstructive airway disease"[Title/Abstract] OR "chronic airflow obstruction"[Title/Abstract] OR "chronic bronchitis"[Title/Abstract] OR "Emphysema"[Title/Abstract] OR "airflow limitation"[Title/Abstract] OR "pulmonary emphysema"[Title/Abstract]) AND ("mental disorders"[MeSH Terms] OR "mental disorders"[MeSH Terms] OR "mental disorders"[MeSH Terms] OR "neurotic disorders"[MeSH Terms] OR "mood disorders"[MeSH Terms] OR "anxiety disorders"[MeSH Terms] OR "depressive disorder"[MeSH Terms] OR "psychotic disorders"[MeSH Terms] OR "psychotic disorders"[MeSH Terms] OR "mental illness"[Title/Abstract] OR "mental health disorder"[Title/Abstract] OR "psychiatric disorder"[Title/Abstract] OR "psychological disorder"[Title/Abstract] OR "emotional disorder"[Title/Abstract] OR "neuropsychiatric disorder"[Title/Abstract] OR "behavioral health disorder"[Title/Abstract] OR "mental health issue"[Title/Abstract] OR "psychiatric condition"[Title/Abstract] OR "psychological condition"[Title/Abstract] OR "Schizophrenia"[MeSH Terms] OR "bipolar disorder"[MeSH Terms] OR ("depressive disorder, major"[MeSH Terms] OR "depressive disorder"[MeSH Terms]) OR (("sever"[All Fields] OR "Severe"[All Fields] OR "severed"[All Fields] OR "severely"[All Fields] OR "severer"[All Fields] OR "severes"[All Fields] OR "severing"[All Fields] OR "severities"[All Fields] OR "severity"[All Fields] OR "severs"[All Fields]) AND "mental disorders"[MeSH Terms]) OR ("Serious"[All Fields] AND "mental disorders"[MeSH Terms]) OR "severe mental illness"[Title/Abstract] OR "serious mental illness"[Title/Abstract] OR "severe psychiatric disorder"[Title/Abstract] OR "serious psychiatric disorder"[Title/Abstract] OR "severe psychological disorder"[Title/Abstract] OR "serious psychological disorder"[Title/Abstract] OR "chronic mental illness"[Title/Abstract] OR "chronic psychiatric disorder"[Title/Abstract] OR "Schizophrenia"[Title/Abstract] OR "bipolar disorder"[Title/Abstract] OR "major depressive disorder"[Title/Abstract] OR "severe anxiety disorder"[Title/Abstract] OR "obsessive compulsive disorder"[Title/Abstract] OR "severe ocd"[Title/Abstract] OR "panic*"[Title/Abstract] OR "post traumatic stress disorder"[Title/Abstract] OR "post traumatic stress disorder"[Title/Abstract] OR "PTSD"[Title/Abstract]) AND ("outcome*"[Title/Abstract] OR "association*"[Title/Abstract] OR "exacerbation*"[Title/Abstract] OR "quality of life"[Title/Abstract] OR "quality*"[Title/Abstract] OR "FEV1"[Title/Abstract] OR "forced expiratory*"[Title/Abstract] OR "DLCO"[Title/Abstract] OR "diffus*"[Title/Abstract] OR "TLCO"[Title/Abstract] OR "hospitalisation"[Title/Abstract] OR "hospitalization"[Title/Abstract] OR "mortality"[Title/Abstract])
**71**	"outcome*"[Title/Abstract] OR "association*"[Title/Abstract] OR "exacerbation*"[Title/Abstract] OR "quality of life"[Title/Abstract] OR "quality*"[Title/Abstract] OR "FEV1"[Title/Abstract] OR "forced expiratory*"[Title/Abstract] OR "DLCO"[Title/Abstract] OR "diffus*"[Title/Abstract] OR "TLCO"[Title/Abstract] OR "hospitalisation"[Title/Abstract] OR "hospitalization"[Title/Abstract] OR "mortality"[Title/Abstract]

Alsaab S, *et al. BMJ Open Respir Res* 2026; 13:e003977. doi: 10.1136/bmjresp-2025-003977

**70**	"mortality"[Title/Abstract]
**69**	"hospitalization"[Title/Abstract]
**68**	"hospitalisation"[Title/Abstract]
**67**	"TLCO"[Title/Abstract]
**66**	"diffus*"[Title/Abstract]
**65**	"DLCO"[Title/Abstract]
**64**	"forced expiratory*"[Title/Abstract]
**63**	"FEV1"[Title/Abstract]
**62**	"quality*"[Title/Abstract]
**61**	"quality of life"[Title/Abstract]
**60**	"exacerbation*"[Title/Abstract]
**59**	"association*"[Title/Abstract]
**58**	"outcome*"[Title/Abstract]
**57**	("pulmonary disease, chronic obstructive"[MeSH Terms] OR "lung diseases, obstructive"[MeSH Terms] OR "bronchitis, chronic"[MeSH Terms] OR ("pulmonary emphysema"[MeSH Terms] OR "Emphysema"[MeSH Terms]) OR "chronic obstructive pulmonary disease"[Title/Abstract] OR "chronic obstructive lung disease"[Title/Abstract] OR "COPD"[Title/Abstract] OR "chronic obstructive airway disease"[Title/Abstract] OR "chronic airflow obstruction"[Title/Abstract] OR "chronic bronchitis"[Title/Abstract] OR "Emphysema"[Title/Abstract] OR "airflow limitation"[Title/Abstract] OR "pulmonary emphysema"[Title/Abstract]) AND ("mental disorders"[MeSH Terms] OR "mental disorders"[MeSH Terms] OR "mental disorders"[MeSH Terms] OR "neurotic disorders"[MeSH Terms] OR "mood disorders"[MeSH Terms] OR "anxiety disorders"[MeSH Terms] OR "depressive disorder"[MeSH Terms] OR "psychotic disorders"[MeSH Terms] OR "psychotic disorders"[MeSH Terms] OR "mental illness"[Title/Abstract] OR "mental health disorder"[Title/Abstract] OR "psychiatric disorder"[Title/Abstract] OR "psychological disorder"[Title/Abstract] OR "emotional disorder"[Title/Abstract] OR "neuropsychiatric disorder"[Title/Abstract] OR "behavioral health disorder"[Title/Abstract] OR "mental health issue"[Title/Abstract] OR "psychiatric condition"[Title/Abstract] OR "psychological condition"[Title/Abstract] OR "Schizophrenia"[MeSH Terms] OR "bipolar disorder"[MeSH Terms] OR ("depressive disorder, major"[MeSH Terms] OR "depressive disorder"[MeSH Terms]) OR (("sever"[All Fields] OR "Severe"[All Fields] OR "severed"[All Fields] OR "severely"[All Fields] OR "severer"[All Fields] OR "severes"[All Fields] OR "severing"[All Fields] OR "severities"[All Fields] OR "severity"[All Fields] OR "severs"[All Fields]) AND "mental disorders"[MeSH Terms]) OR ("Serious"[All Fields] AND "mental disorders"[MeSH Terms]) OR "severe mental illness"[Title/Abstract] OR "serious mental illness"[Title/Abstract] OR "severe psychiatric disorder"[Title/Abstract] OR "serious psychiatric disorder"[Title/Abstract] OR "severe psychological disorder"[Title/Abstract] OR "serious psychological disorder"[Title/Abstract] OR "chronic mental illness"[Title/Abstract] OR "chronic psychiatric disorder"[Title/Abstract] OR "Schizophrenia"[Title/Abstract] OR "bipolar disorder"[Title/Abstract] OR "major depressive disorder"[Title/Abstract] OR "severe anxiety disorder"[Title/Abstract] OR "obsessive compulsive disorder"[Title/Abstract] OR "severe ocd"[Title/Abstract] OR "panic*"[Title/Abstract] OR "post traumatic stress disorder"[Title/Abstract] OR "post traumatic stress disorder"[Title/Abstract] OR "PTSD"[Title/Abstract])

Alsaab S, *et al. BMJ Open Respir Res* 2026; 13:e003977. doi: 10.1136/bmjresp-2025-003977

56	"mental disorders"[MeSH Terms] OR "mental disorders"[MeSH Terms] OR "mental disorders"[MeSH Terms] OR "neurotic disorders"[MeSH Terms] OR "mood disorders"[MeSH Terms] OR "anxiety disorders"[MeSH Terms] OR "depressive disorder"[MeSH Terms] OR "psychotic disorders"[MeSH Terms] OR "psychotic disorders"[MeSH Terms] OR "mental illness"[Title/Abstract] OR "mental health disorder"[Title/Abstract] OR "psychiatric disorder"[Title/Abstract] OR "psychological disorder"[Title/Abstract] OR "emotional disorder"[Title/Abstract] OR "neuropsychiatric disorder"[Title/Abstract] OR "behavioral health disorder"[Title/Abstract] OR "mental health issue"[Title/Abstract] OR "psychiatric condition"[Title/Abstract] OR "psychological condition"[Title/Abstract] OR "Schizophrenia"[MeSH Terms] OR "bipolar disorder"[MeSH Terms] OR ("depressive disorder, major"[MeSH Terms] OR "depressive disorder"[MeSH Terms]) OR (("sever"[All Fields] OR "Severe"[All Fields] OR "severed"[All Fields] OR "severely"[All Fields] OR "severer"[All Fields] OR "severes"[All Fields] OR "severing"[All Fields] OR "severities"[All Fields] OR "severity"[All Fields] OR "severs"[All Fields]) AND "mental disorders"[MeSH Terms]) OR ("Serious"[All Fields] AND "mental disorders"[MeSH Terms]) OR "severe mental illness"[Title/Abstract] OR "serious mental illness"[Title/Abstract] OR "severe psychiatric disorder"[Title/Abstract] OR "serious psychiatric disorder"[Title/Abstract] OR "severe psychological disorder"[Title/Abstract] OR "serious psychological disorder"[Title/Abstract] OR "chronic mental illness"[Title/Abstract] OR "chronic psychiatric disorder"[Title/Abstract] OR "Schizophrenia"[Title/Abstract] OR "bipolar disorder"[Title/Abstract] OR "major depressive disorder"[Title/Abstract] OR "severe anxiety disorder"[Title/Abstract] OR "obsessive compulsive disorder"[Title/Abstract] OR "severe ocd"[Title/Abstract] OR "panic*"[Title/Abstract] OR "post traumatic stress disorder"[Title/Abstract] OR "post traumatic stress disorder"[Title/Abstract] OR "PTSD"[Title/Abstract]
55	"PTSD"[Title/Abstract]
53	"post traumatic stress disorder"[Title/Abstract]
52	"panic*"[Title/Abstract]
51	"severe ocd"[Title/Abstract]
50	"obsessive compulsive disorder"[Title/Abstract]
49	"severe anxiety disorder"[Title/Abstract]
48	"major depressive disorder"[Title/Abstract]
47	"bipolar disorder"[Title/Abstract]
46	"Schizophrenia"[Title/Abstract]
45	"chronic psychiatric disorder"[Title/Abstract]
44	"chronic mental illness"[Title/Abstract]
43	"serious psychological disorder"[Title/Abstract]
42	"severe psychological disorder"[Title/Abstract]
41	"serious psychiatric disorder"[Title/Abstract]
40	"severe psychiatric disorder"[Title/Abstract]
39	"serious mental illness"[Title/Abstract]
38	"severe mental illness"[Title/Abstract]
35	"depressive disorder, major"[MeSH Terms] OR "depressive disorder"[MeSH Terms]
34	"bipolar disorder"[MeSH Terms]
33	"schizophrenia"[MeSH Terms]
32	"psychological condition"[Title/Abstract]

**31**	"psychiatric condition"[Title/Abstract]
**30**	"mental health issue"[Title/Abstract]
**29**	"behavioral health disorder"[Title/Abstract]
**28**	"neuropsychiatric disorder"[Title/Abstract]
**27**	"emotional disorder"[Title/Abstract]
**26**	"psychological disorder"[Title/Abstract]
**25**	"psychiatric disorder"[Title/Abstract]
**24**	"mental health disorder"[Title/Abstract]
**23**	"mental illness"[Title/Abstract]
**22**	"psychotic disorders"[MeSH Terms]
**21**	"depressive disorder"[MeSH Terms]
**20**	"anxiety disorders"[MeSH Terms]
**19**	"mood disorders"[MeSH Terms]
**18**	"neurotic disorders"[MeSH Terms]
**17**	"mental disorders"[MeSH Terms]
**16**	"mental disorders"[MeSH Terms]
**15**	"mental disorders"[MeSH Terms]
**14**	"pulmonary disease, chronic obstructive"[MeSH Terms] OR "lung diseases, obstructive"[MeSH Terms] OR "bronchitis, chronic"[MeSH Terms] OR "pulmonary emphysema"[MeSH Terms] OR "Emphysema"[MeSH Terms] OR "chronic obstructive pulmonary disease"[Title/Abstract] OR "chronic obstructive lung disease"[Title/Abstract] OR "COPD"[Title/Abstract] OR "chronic obstructive airway disease"[Title/Abstract] OR "chronic airflow obstruction"[Title/Abstract] OR "chronic bronchitis"[Title/Abstract] OR "Emphysema"[Title/Abstract] OR "airflow limitation"[Title/Abstract] OR "pulmonary emphysema"[Title/Abstract]
**13**	"pulmonary emphysema"[Title/Abstract]
**12**	"airflow limitation"[Title/Abstract]
**11**	"Emphysema"[Title/Abstract]
**10**	"chronic bronchitis"[Title/Abstract]
**9**	"chronic airflow obstruction"[Title/Abstract]
**8**	"chronic obstructive airway disease"[Title/Abstract]
**7**	"COPD"[Title/Abstract]
**6**	"chronic obstructive lung disease"[Title/Abstract]
**5**	"chronic obstructive pulmonary disease"[Title/Abstract]
**4**	"pulmonary emphysema"[MeSH Terms] OR "emphysema"[MeSH Terms]
**3**	"bronchitis, chronic"[MeSH Terms]
**2**	"lung diseases, obstructive"[MeSH Terms]
**1**	"pulmonary disease, chronic obstructive"[MeSH Terms]

Alsaab S, *et al. BMJ Open Respir Res* 2026; 13:e003977. doi: 10.1136/bmjresp-2025-003977

Additional methods:

Rating of quality using Newcastle Ottawa Scale

For cohort studies, selection evaluated representativeness of the exposed cohort, selection of the non-exposed cohort, ascertainment of exposure (mental illnesses), and demonstration that the outcome of interest was not present at the study's start. Comparability assessed whether studies controlled for confounding factors, specifically age, smoking history, and additional relevant factors (e.g., socioeconomic status, COPD severity). Outcome evaluation assessed the method of outcome ascertainment, adequacy of follow-up duration, and completeness of follow-up.

For case-control studies, the selection domain considered adequacy of case definitions, representativeness of cases, control selection, and definition of controls. Comparability followed the same criteria as for cohort studies. Exposure assessment evaluated ascertainment methods, consistency between cases and controls, and response rates.

Cross-sectional studies used an adapted NOS, assessing selection based on sample representativeness, sample size justification, exposure ascertainment, and selection of the non-exposed group. Comparability followed identical criteria to other study types. Outcome evaluation considered assessment methods, appropriateness and clarity of statistical tests, and completeness and description of data availability (Nayebirad et al., 2023).

Newcastle-Ottwa scale (NOS) risk of bias assessment templates

Cohort studies

Selection:

1) Representativeness of the Exposed Cohort

a) truly representative of the average COPD patients in the community ★

b) somewhat representative of COPD patients in the community ★

c) selected group of patients (e.g., patients from specialised clinics, volunteers)

d) no description of the derivation of the cohort

2) Selection of the Non-Exposed Cohort

a) Drawn from the same community as the exposed cohort ★

b) Drawn from a different source

c) No description of the derivation of the non-exposed cohort

3) Ascertainment of Exposure (Psychiatric Comorbidities)

a) Secure record (e.g., medical or mental health records) ★

b) Structured interview ★

c) Written self-report

d) No description

4) Demonstration that patient selection was not based worst outcome of interest at start of study/ outcome of interest was not present at start of study

a) Yes ★

b) No

Comparability:

1) Comparability of groups

a) Study controls for age and smoking history ★

b) Study controls for any additional factor (e.g., socioeconomic status, COPD severity) ★★

Alsaab S, *et al. BMJ Open Respir Res* 2026; 13:e003977. doi: 10.1136/bmjresp-2025-003977

Outcome:

1) Assessment of outcome

a) Independent blind assessment ★

b) Record linkage ★

c) Self-report

d) No description

2) Was follow-up long enough for outcomes to occur

a) Yes (e.g., at least 1 year follow-up) ★

b) No

3) Adequacy of follow-up of cohorts

a) Complete follow-up, all subjects accounted for ★

b) Subjects lost to follow-up unlikely to introduce bias, small number lost, >90% follow-up, or description provided of those lost ★

c) Follow-up rate <90% and no description of those lost

d) No statement

Alsaab S, *et al. BMJ Open Respir Res* 2026; 13:e003977. doi: 10.1136/bmjresp-2025-003977

Case-control studies

Selection:

1) Is the case definition adequate?

a) yes, with independent validation (e.g., clinical diagnosis of COPD with psychiatric comorbidity) ★

b) yes, e.g., record linkage or based on self-reports

c) no description

2) Representativeness of the cases

a) consecutive or obviously representative series of cases (e.g., COPD patients from general practice) ★

b) potential for selection biases or not stated

3) Selection of controls

a) community controls (COPD patients without psychiatric comorbidity) ★

b) hospital controls

c) no description

4) Definition of controls

a) no presence of psychiatric comorbidities ★

b) no description of controls

Comparability:

1) Comparability of groups

a) study controls for age and smoking history ★

b) study controls for any additional factor (e.g., socioeconomic status, COPD severity) ★★

Alsaab S, *et al. BMJ Open Respir Res* 2026; 13:e003977. doi: 10.1136/bmjresp-2025-003977

Exposure:

1) Ascertainment of exposure (psychiatric comorbidities)

a) secure record (e.g., medical or mental health records) ★

b) structured interview where blind to case/control status ★

c) interview not blinded to case/control status

d) written self-report

e) no description

2) Same method of ascertainment for cases and controls

a) yes ★

b) no

3) Non-response rate

a) same rate for both groups ★

b) non-respondents described

c) rate different and no designation

Alsaab S, *et al. BMJ Open Respir Res* 2026; 13:e003977. doi: 10.1136/bmjresp-2025-003977

Cross-sectional studies

Selection:

1) Representativeness of the sample

a) Truly representative of the average COPD patients in the community ★

b) Somewhat representative of COPD patients in the community ★

c) Selected group of patients (e.g., patients from specialised clinics, volunteers)

d) No description of the sample selection

2) Sample size

a) justified and satisfactory (> 200 patient included) ★

b) not justified (<200 patient included)

3) Ascertainment of exposure (psychiatric comorbidities)

a) secure record (e.g., medical or mental health records) ★

b) structured interview using standardised diagnostic tools ★

c) self-reported diagnosis

d) no description

4) Selection of non-exposed group

a) COPD patients without psychiatric comorbidities, drawn from the same population ★

b) COPD patients with other characteristics, or non-comparable selection

c) no description of non-exposed group

Comparability:

1) Controlling for confounding factors

a) study controls for age and smoking history ★

b) study controls for any additional factor (e.g., socioeconomic status, COPD severity) ★★

Alsaab S, et al. BMJ Open Respir Res 2026; 13:e003977. doi: 10.1136/bmjresp-2025-003977

Outcome:

1) Assessment of outcome

a) objective assessment with validated tools (e.g., clinical scales, medical records) ★

b) self-report with validated questionnaires ★

c) self-report with non-validated methods

d) no description

2) Statistical tests

a) Statistical test used to analyse the data clearly described, appropriate, and measures of association presented including confidence intervals and probability level (P value) ★

b) Statistical test not appropriate, not described, or incomplete

3) Data availability

a) ≥90% of patients' data available and missing data described ★

b) 60%-90% of patients' data available with limited description of missing data

c) <60% of patients' data available or no description of missing data

After reviewing the literature, NOS for cross-sectional studies was adapted with modifications that suits our systematic review and keep the total out of 9 stars, adapted from (Nayebirad et al., 2023).

Alsaab S, *et al. BMJ Open Respir Res* 2026; 13:e003977. doi: 10.1136/bmjresp-2025-003977

Additional results:

Study characteristics:

Supplement, table 1: table of characteristics of the included studies.

Study ID (country)	Exposures	Diagnosis tool	Study design	Age	Male %	GOLD stages	n (COPD+ mental illness)	n (COPD without mental illness)	Settings	Outcomes measured
**Aldhahi 2023 (Saudi Arabia)**	Anxiety	AIR >= 8. HADS, >= 8	Cross-sectional	63 (SD 11)	80%	1-4	32	38	Outpatient	FEV_1, SGRQ, 6MWT
**Eisner 2010 (United States)**	Anxiety	HADS-A, >= 8	Cohort	58.33 (SD 6.25)	42.7 %	NR	181	1024	Outpatient	FEV_1
**Mou 2024 (China)**	Anxiety	HAMA, >= 14	Cohort	70 (IQR 64-78)	89.6 %	NR	84	340	Outpatient	SGRQ, Exacerbations, CAT, mMRC, 6MWT
**Yohannes 2024 (United Kingdom)**	Anxiety	AIR >=8.	Cohort	71.51 (SD 8.76)	51.4 %	NR	348	645	Outpatient	SGRQ, mMRC
**An 2010 (China)**	Anxiety ; Depression	HADS, >= 8	Cross-sectional	64 (SD 8.4)	79.7 %	NR	48	208	Outpatient	FEV_1, SGRQ, mMRC, 6MWT
**Bugajski 2023 (United States)**	Anxiety ; Depression; Mixed Anxiety and Depression	Anxiety=(STAI) >=39. Depression= (BDI) >= 11.	Cross-sectional	67.5 (SD 5.5)	61.5 %	3 and 4	884	876	Outpatient	SGRQ, 6MWT
**Marco 2006 (Italy)**	Anxiety ; Depression	Anxiety (STAI) >45. Depression (SDS) >50.	Cross-sectional	68 (SD 14.21)	76.7 %	NR	57	145	Outpatient	FEV_1, SGRQ, mMRC

Alsaab S, *et al. BMJ Open Respir Res* 2026; 13:e003977. doi: 10.1136/bmjresp-2025-003977

**Dua 2018 (India)**	Mixed Anxiety and Depression	HADS, >=8	Cross-sectional	61 (SD 6.6)	91.4 %	1-4	40	88	Outpatient	FEV_1, Exacerbation, Hospitalisation, mMRC, 6MWT
**Feng 2022 (China)**	Anxiety ; Depression ; Mixed Anxiety and Depression	ICD-10	Cohort	77 (IQR 69-83)	66%	NR	7912	374213	Inpatient	Hospitalisation, LoS
**Gonzalez-Gutierrez 2016 (Spain)**	Mixed Anxiety and Depression	ICD-10 and HADS. Then patients are interviewed by a psychiatrist.	Cross-sectional	66.2 (SD 9.5)	90.2 %	NR	74	130	Outpatient	Exacerbation, mMRC, 6MWT
**Gudmundsson 2006 (Iceland, Sweden, Denmark, Finland and Norway)**	Anxiety ; Depression; Mixed Anxiety and Depression	HADS, >= 8	Cohort	69.4 (SD 10.4)	49.3 %	NR	191	202	Inpatient	FEV_1, SGRQ
**Hong 2023 (Korea)**	Anxiety ; Depression	BDI-II for depression, >10. BAI for anxiety, >8.	Cohort	68.9 (SD 7.7)	93.1 %	1-4	532	1713	Outpatient	FEV_1, DLCO, SGRQ, Exacerbation, CAT, mMRC, 6MWT
**Kuhl 2008 (Germany)**	Mixed Anxiety and Depression	ICD-10	Cross-sectional	67 (SD 9.5)	78.3 %	NR	28	115	Outpatient	FEV_1, SGRQ, 6MWT
**Long 2020 (China)**	Mixed Anxiety and Depression	for anxiety HAMA, >= 14. for depression HAMD, >= 20.	Cross-sectional	68.5 (SD 10.6)	65.1 %	1-4	195	112	Outpatient	Exacerbation, CAT

Alsaab S, *et al. BMJ Open Respir Res* 2026; 13:e003977. doi: 10.1136/bmjresp-2025-003977

**Lou 2012 (China)**	Anxiety ; Depression	HADS-A, >= 8. HADS-D, >= 8.	Cross-sectional	63.2 (IQR 40-75)	75.2 %	1-4	393	707	Outpatient	FEV_1, SGRQ, mMRC, 6MWT
**Mi 2017 (United Kingdom)**	Anxiety ; Depression	HADS, >=8	Cross-sectional	66-74.5	60.7 %	NR	52	60	Outpatient	FEV_1, Exacerbation, mMRC
**Montserrat-Capdevila 2018 (Spain)**	Mixed Anxiety and Depression	HADS, cutoff >= 10	Cohort	69.5 (SD 12.2)	73.2 %	NR	80	432	Outpatient	Hospitalisation
**Vikjord 2020 (Norway)**	Anxiety ; Depression	HADS-A, >= 8. HADS-D, >= 8.	Cohort	62.1 (SD 11.2)	54.6 %	1-4	668	1239	Outpatient	FEV_1, Mortality
**Xu 2008 (China)**	Anxiety ; Depression	HADS-A, HADS-D, >= 8.	Cohort	65.6 (SD 10.69)	68.8 %	1-3 vs.4	112	379	Outpatient	FEV_1, SGRQ, Exacerbation, Hospitalisation, LoS, Mortality, mMRC, 6MWT
**Zhang 2014 (China)**	Mixed Anxiety and Depression	HADS, >= 8	Cross-sectional	65.64 (SD 7.6)	91.1 %	NR	95	264	Outpatient	FEV_1, CAT, mMRC, 6MWT
**Abrams 2011 (United States)**	Anxiety ; Depression ; SMI	ICD-9	Cohort	69.12 (SD 10.4)	97.8 %	NR	6430	21726	Inpatient	Hospitalisation, Mortality
**Regvat 2011 (Slovenia)**	Mixed Anxiety; Depression and SMI	PRIME-MD	Cohort	72.4 (SD 8.4)	70%	1-4	25	25	Inpatient	FEV_1, Hospitalisation, 6MWT
**Mehta 2014 (India)**	Anxiety ; SMI	interview	Cross-sectional	59.7 (SD 9.26)	NR	NR	19	40	Outpatient	FEV_1, SGRQ, Exacerbation, CAT, mMRC
**Al-shair 2009 (United Kingdom)**	Depression	CES-D, >=16	Cross-sectional	66 (SD 6.7)	61.5 %	NR	29	93	Outpatient	FEV_1, SGRQ, mMRC, 6MWT
**Biswas 2017 (India)**	Depression	HAM-D, >= 8	Cross-sectional	62.09 (SD 9.15)	90.7 %	NR	41	34	Outpatient	FEV_1, 6MWT

Alsaab S, *et al. BMJ Open Respir Res* 2026; 13:e003977. doi: 10.1136/bmjresp-2025-003977

**Chavannes 2005 (Netherlands)**	Depression	BDI , >10	Cross-sectional	58.6 (SD 10.2)	75.5 %	NR	40	107	Outpatient	FEV_1, Exacerbation, mMRC
**Dalal 2011 (United states)**	Depression	ICD-9	Cohort	63.55 (SD 11.55)	36.9 %	NR	3761	3761	Outpatient; Inpatient	Hospitalisation
**Marco 2014 (Italy)**	Depression	HADS, >= 8	Cross-sectional	71 (SD 6)	74%	NR	19	51	Outpatient	FEV_1, DLCO, SGRQ, Exacerbation, mMRC, 6MWT
**Fan 2007 (United states)**	Depression	BDI >= 10	Cohort	66.5 (SD 5.85)	64.1 %	NR	249	361	Outpatient; Inpatient	FEV_1, SGRQ, Hospitalisation, Mortality, 6MWT
**Hanania 2011 (12 countries)**	Depression	CES-D, >= 16.	Cohort	63 (SD 7)	65%	NR	557	1561	Outpatient	FEV_1, SGRQ, Exacerbation, Hospitalisation, mMRC, 6MWT
**Horita 2013 (Japan)**	Depression	SF-GDS, >=6	Cross-sectional	72 (SD 9)	82.1 %	1-4	32	52	Outpatient	FEV_1, mMRC, 6MWT
**Horner 2023 (Austria)**	Depression	PHQ-9, >= 5	Cross-sectional	66.8 (SD 8.6)	62.5 4%	1-4	284	331	Outpatient	FEV_1, SGRQ, Exacerbation
**Iguchi 2013 (Japan)**	Depression	CES-D, >= 16	Cross-sectional	73.12 (SD 7.34)	86.5 %	NR	36	38	Inpatient	FEV_1, SGRQ, mMRC, 6MWT
**Ito 2012 (Japan)**	Depression	CES-D, >= 16	Cohort	70 (SD 7.9)	90.6 %	1-4	14	46	Outpatient	FEV_1, SGRQ, Exacerbation, mMRC
**Kil 2010 (Korea)**	Depression	BDI, >= 16	Cross-sectional	69.3 (SD 8.2)	85.7 %	NR	16	77	Outpatient	FEV_1, SGRQ
**Kim 2014 (Korea)**	Depression	CES-D, >= 24	Cross-sectional	66.91 (SD 8.16)	92%	NR	43	202	Outpatient	FEV_1, mMRC, 6MWT
**Lee 2018 (Korea)**	Depression	PHQ-9 >= 5	Cross sectional	64.7 (SD 9)	98.6 %	NR	30	181	Outpatient	FEV_1

**Lim 2019 (Korea)**	Depression	BDI-II, >= 17	Cohort	70.1 (SD 7.6)	89.3 %	1-4	53	217	Outpatient	FEV_1, SGRQ, Exacerbation, Hospitalisation, CAT, 6MWT
**Rivera 2016 (Spain)**	Depression	HADS, >= 8.	Cross sectional	66.9 (SD 8.8)	93%	NR	28	87	Outpatient	FEV_1, Hospitalisation, mMRC, 6MWT
**Miravitlles 2014 (Spain)**	Depression	BDI, >=5.	Cross sectional	68.3 (SD 9.3)	83%	NR	642	217	Outpatient	FEV_1, Exacerbation, Hospitalisation, CAT
**Narod 2023 (India)**	Depression	MADRS	Cohort	NR	NR	NR	51	44	Outpatient	FEV_1, 6MWT
**Ng 2007 (Singapore)**	Depression	HAD, >= 8	Cohort	72.2 (SD 8.33)	85.1 %	NR	167	209	Inpatient	FEV_1, SGRQ, Hospitalisation, LoS, Mortality
**Orlandi 2016 (Israel)**	Depression	MINI + interview	Cross sectional	68.93 (SD 9)	55.5 5%	NR	12	42	Outpatient	FEV_1, Hospitalisation, mMRC
**Papaioannou 2013 (Greece)**	Depression	BDI, >=19	Cohort	71.2 (SD 8.8)	88.3 %	1-4	91	139	Inpatient	FEV_1, Exacerbation, Hospitalisation, LoS, Mortality
**Patange 2023 (India)**	Depression	HAM-D	Cross sectional	NR	NR	NR	33	32	Outpatient	FEV_1, 6MWT
**Tse 2016 (China)**	Depression	GDS; >= 8	Cross sectional	75.2 (SD 0.82)	89.9 %	NR	18	71	Outpatient	FEV_1, SGRQ, Exacerbation, Hospitalisation, mMRC, 6MWT
**Venkata 2012 (United States)**	Depression	HADS, >=10	Cohort	69.11 (SD 6.66)	52.8 %	NR	16	20	Outpatient	FEV_1, Exacerbation, mMRC, 6MWT
**Xu 2018 (China)**	Depression	(HDRS) or HAM-D, >= 7	Cohort	77.74 (IQR 52-82)	69.8 %	NR	40	13	Inpatient	FEV_1
**Yohannes 2016 (12 countries)**	Depression	CES-D, >= 16	Cohort	62.96 (SD 9.94)	64%	NR	377	869	Outpatient	FEV_1, SGRQ, mMRC, 6MWT

Alsaab S, et al. BMJ Open Respir Res 2026; 13:e003977. doi: 10.1136/bmjresp-2025-003977

**Yohannes 2017 (12 countries)**	Depression	CES-D >=16	Cohort	63.4 (SD 7.1)	65.3 %	NR	540	1519	Outpatient	FEV_1, SGRQ, mMRC, 6MWT
**Giardino 2010 (United states)**	SMI	(M.I.N.I) and (ADIS-IV)	Case control	65.43 (SD 3.39)	68.4 %	NR	10	9	Outpatient	FEV_1
**Goldman 2023 (United Kingdom)**	SMI	ICD-10	Cohort	62.46 (SD 10.76)	47.6 %	NR	2096	52482	Inpatient	Hospitalisation, LoS, Mortality
**Jorgensen 2018 (Denmark)**	SMI	ICD-10	Cohort	67.22	45.2 %	1-4	1502	210366	Outpatient; Inpatient	Hospitalisation, Mortality
**Laurin 2009 (Canada)**	SMI	ADIS-IV based on the DSM-IV	Cohort	66 (SD 8)	49%	NR	54	56	Outpatient	FEV_1, Exacerbation, mMRC
**Livermore 2012 (Australia)**	SMI	ADIS-IV	Cross sectional	63 (SD 11)	44%	NR	25	37	Outpatient	FEV_1, SGRQ
**Blakemore 2019 (United Kingdom)**	Anxiety ; Depression	HAD, >= 8	Cohort	Median 60-79	52.7 %	1-4	NR	NR	Outpatient	Hospitalisation
**Iyer 2016 (United States)**	Anxiety ; Depression	Physician diagnosis or by use of medications	Cohort	64.8 (SD 11.7)	50.2 %	NR	NR	NR	Inpatient	Hospitalisation
**Martinez-Gestoso 2022 (Spain)**	Anxiety ; Depression	HADS, >= 11	Cohort	73.7 (SD 10.9)	84.7 %	1-4	197	91	Inpatient	Hospitalisation, Mortality

NR= not reported. Hospital Anxiety and Depression Scale (HADS), State-Trait Anxiety Inventory (STAI), Beck Anxiety Inventory (BAI), Hamilton Anxiety Rating Scale (HAMA), Beck Depression Inventory (BDI), Hamilton Depression Rating Scale (HAM-D), Primary Care Evaluation of Mental Disorders (PRIME-MD), Montgomery-Asberg Depression Rating Scale (MADRS), Patient Health Questionnaire (PHQ-9), Centre for Epidemiologic Studies Depression Scale (CES-D), Zung Self-Rating Depression Scale (SDS), Geriatric Depression Scale (GDS), international classification of diseases, tenth revision (ICD-10), Diagnostic and Statistical Manual of Mental Disorders, fourth edition (DSM-IV), Mini International Neuropsychiatric Interview (M.I.N.I), Anxiety Disorders Interview Schedule (ADIS-IV).

NOS risk of bias assessment table of included studies

Supplement, table 2: Bias assessment table using Newcastle-Ottwa scale (NOS).

#	Study ID	Selection	Comparability	Outcome	Total
1	Abrams 2011	3 stars	2 stars	3 stars	8 stars
2	Aldhahi 2023	3 stars	1 star	3 stars	7 stars
3	Al-shair 2009	3 stars	0 star	3 stars	6 stars
4	An 2010	2 stars	1 star	3 stars	6 stars
5	Biswas 2017	2 stars	1 star	3 stars	6 stars
6	Bugajski 2023	3 stars	0 stars	3 stars	6 stars
7	Chavannes 2005	2 stars	0 stars	3 stars	5 stars
8	Dalal 2011	4 stars	1 star	3 stars	8 stars
9	Dua 2018	3 stars	1 star	3 stars	7 stars
10	Eisner 2010	4 stars	2 stars	3 stars	9 stars
11	Fan 2007	3 stars	1 star	3 stars	7 stars
12	Feng 2022	4 stars	1 star	2 stars	7 stars
13	Giardino 2010	1 star	0 star	2 stars	3 stars
14	Goldman 2023	4 stars	1 star	3 stars	8 stars
15	Gonzalez-Gutierrez 2016	3 stars	0 stars	3 stars	6 stars
16	Gudmundsson 2006	4 stars	0 star	2 stars	6 stars
17	Hanania 2011	4 stars	0 star	3 stars	7 stars
18	Hong 2023	4 stars	2 stars	3 stars	9 stars
19	Horita 2013	3 stars	0 star	3 stars	6 stars
20	Horner 2023	3 stars	0 star	3 stars	6 stars
21	Iguchi 2013	1 star	0 star	3 stars	4 stars
22	Ito 2012	4 stars	2 stars	2 stars	8 stars
23	Iyer 2016	2 stars	2 stars	3 stars	7 stars
24	Jorgensen 2018	4 stars	1 star	2 stars	7 stars
25	Kil 2010	1 star	0 star	3 stars	4 stars
26	Kim 2014	4 stars	0 star	3 stars	7 stars
27	Kuhl 2008	2 stars	0 star	3 stars	5 stars
28	Laurin 2009	4 stars	1 star	2 stars	7 stars
29	Lee 2018	4 stars	0 star	2 stars	6 stars
30	Lim 2019	3 stars	0 star	3 stars	6 stars
31	Livermore 2012	2 stars	0 star	2 stars	4 stars
32	Long 2020	3 stars	0 star	3 stars	6 stars
33	Lou 2012	3 stars	0 star	3 stars	6 stars
34	Marco 2006	3 stars	0 star	3 stars	6 stars
35	Marco 2014	2 stars	0 star	3 stars	5 stars
36	Mehta 2014	2 stars	0 star	3 stars	5 stars
37	Mi 2017	2 stars	0 star	3 stars	5 stars

Alsaab S, *et al. BMJ Open Respir Res* 2026; 13:e003977. doi: 10.1136/bmjresp-2025-003977

38	Miravitlles 2014	3 stars	1 star	3 stars	7 stars
39	Montserrat-Capdevila 2018	3 stars	2 stars	3 stars	8 stars
40	Mou 2024	3 stars	1 star	3 stars	7 stars
41	Narod 2023	2 stars	0 stars	1 star	3 stars
42	Ng 2007	2 stars	1 star	2 stars	5 stars
43	Orlandi 2016	2 stars	0 star	3 stars	5 stars
44	Papaioannou 2013	2 stars	2 star	3 stars	7 stars
45	Patange 2023	1 star	0 star	3 stars	4 stars
46	Regvat 2011	2 stars	0 star	1 star	3 stars
47	Rivera 2016	3 stars	0 star	3 stars	6 stars
48	Tse 2016	2 stars	1 star	2 stars	5 stars
49	Venkata 2012	2 stars	0 star	1 star	3 stars
50	Vikjord 2020	4 stars	2 stars	2 stars	6 stars
51	Xu 2008	4 stars	2 stars	2 stars	6 stars
52	Xu 2018	2 stars	0 star	3 stars	5 stars
53	Yohannes 2016	4 stars	0 stars	3 stars	7 stars
54	Yohannes 2017	4 stars	1 star	2 stars	7 stars
55	Yohannes 2024	3 stars	0 stars	3 stars	6 stars
56	Zhang 2014	4 stars	0 stars	3 stars	7 stars
57	Blakemore 2019	3 stars	2 stars	3 stars	8 stars
58	Martinez-Gestoso 2022	4 stars	1 star	3 stars	8 stars

Selection domain is out of 4 stars, Comparability domain out of 2 stars and outcome domain is out of 3 stars. Total quality scores were categorised based on predefined thresholds adapted from (Zulkipli et al., 2018). 7-9 stars as good quality, 5-6 fair, and 0-4 poor.

Physiological outcomes and functional status

FEV_1%

Sensitivity analysis

A sensitivity analysis was conducted to evaluate the robustness of these findings (Supplement, table 3) by excluding studies with poor quality and stratifying results based on study design (cohort and cross-sectional studies). The exclusion of poor quality studies showed results consistent with the primary analysis, with depression remaining associated with lower FEV_1% (MD: -3.20 [95% CI: -5.15 to -1.26]), while anxiety and SMI remained non-significant. Stratification by study design revealed that in cohort studies, depression was still associated with lower FEV_1% (MD: -3.77 [95% CI: -6.55 to -1.00]), whereas in cross-sectional studies, the association was no longer significant (MD: -2.71 [95% CI: -5.69 to 0.26]). Anxiety and SMI remained non-significant in both study designs, with confidence intervals crossing the null value. Heterogeneity remained high across all analyses, with I^2 values ranging from 79% to 88%, indicating persistent variability between studies even after stratification.

Supplement, table 3: Sensitivity analysis of FEV1%.

**Subgroup**	**Mean difference [95% CI]**	**Fair and high quality**	**Cohort**	**Cross-sectional**
Depression	**MD: -3.74 [-5.53, -1.95]**	**MD: -3.20 [-5.15, -1.26]**	**MD: -3.77 [-6.55, -1.00]**	MD: -2.71 [-5.69, 0.26]
Anxiety	MD: -0.66 [-2.16, 0.85]	MD: -0.66 [-2.16, 0.85]	MD: -1.07 [-2.88, 0.74]	MD: 0.57 [-2.76, 3.89]
SMI	MD: -0.94 [-8.43, 6.54]	MD: 2.61 [-2.70, 7.92]	MD: 3 [-3.62, 9.62]*	MD: 1.90 [-6.98, 10.78]*
Mixed group	MD: -2.95 [-6.14, 0.24]	MD: -2.33 [-5.55, 0.90]	NA	MD: -2.33 [-5.55, 0.90]
**Total**	**MD: -2.92 [-4.35, -1.48]**	**MD: -2.27 [-3.76, -0.78]**	**MD: -2.89 [-5.05, -0.72]**	MD: -1.76 [-3.96, 0.43]
N of studies	44	37	14	23
I^2	86%	83%	88%	79%

**One study remained in this subgroup*

Alsaab S, *et al. BMJ Open Respir Res* 2026; 13:e003977. doi: 10.1136/bmjresp-2025-003977

6MWT

Sensitivity analysis

A sensitivity analysis was conducted to assess the robustness of the findings (Supplement, table 4) by excluding poor quality studies and stratifying results based on study design (cohort and cross-sectional studies). After the removal of low quality studies, the association between depression and reduced 6MWT performance became stronger, with a pooled MD of -50.83 meters (95% CI: -64.74 to -36.92) compared to MD: -40.13 meters in the primary analysis. Similarly, anxiety, which was previously non-significant, became significantly associated with lower 6MWT performance (MD: -43.77 meters [95% CI: -60.19 to -27.36]), suggesting that study quality influenced the observed results. Heterogeneity (I^2) decreased from 91% to 86% in the depression subgroup and from 99% to 53% in the anxiety subgroup, highlighting the role of methodological differences and outlier studies in driving variability.

When stratified by study design, cohort studies still showed a reduction in 6MWT distance for depression (MD: -42.78 meters). Cross-sectional studies showed a stronger reduction in 6MWT for depression (MD: -58.51 meters) and anxiety (MD: -42.59 meters), suggesting a potentially more pronounced effect in cross-sectional analyses. The total pooled effect remained statistically significant across all stratifications, reinforcing the consistent association between mental health comorbidities and reduced 6MWT in COPD patients.

For mixed mental illnesses, results remained largely unchanged after sensitivity analysis, with a pooled MD of -8.65 meters (95% CI: -28.38 to 11.09) and I^2 remaining at 0%, indicating consistency across studies. No data were available for SMI, limiting the ability to draw conclusions about its effect on 6MWT performance.

Overall, the sensitivity analysis confirmed that depression and anxiety remained associated with reduced 6MWT performance, while anxiety became significant after the removal of an outlier study. Heterogeneity decreased considerably, particularly in the anxiety subgroup, suggesting that study quality and methodological differences may explain some of the variability observed in the primary analysis.

Supplement, table 4: Sensitivity analysis of 6MWT

Subgroup	Mean difference [95% CI]	Fair and high quality	Cohort	Cross-sectional
Depression	**MD: -40.13 [-46.83, -33.42]**	**MD: -50.83 [-64.74, -36.92]**	**MD: -42.78 [-62.05, -23.51]**	**MD: -58.51 [-79.28, -37.73]**
Anxiety	MD: 4.09 [-115.04, 123.23]	**MD: -43.77 [-60.19, -27.36]**	**MD: -56.50 [-99.25, -13.75]***	**MD: -42.59 [-61.20, -23.98]**
SMI	NA	NA	NA	NA
Mixed group	MD: -8.66 [-27.53, 10.21]	MD: -8.65 [-28.38, 11.09]	NA	MD: -8.65 [-28.38, 11.09]
**Total**	**MD: -30.42 [-41.03, -19.82]**	**MD: -45.29 [-56.11, -34.46]**	**MD: -43.96 [-61.99, -25.93]**	**MD: -46.49 [-60.64, -32.34]**
N of studies	30	24	7	17
I^2	98%	82%	82%	83%

**One study remained in this subgroup.*

Symptoms severity and quality of life:

SGRQ

Sensitivity analysis

A sensitivity analysis was conducted (Supplement, table 5) by excluding poor quality studies and stratifying by study design (cohort and cross-sectional). The overall pooled effect remained consistent, with COPD patients with mental illnesses continuing to show worse HRQoL. After excluding low-quality studies, the total pooled MD slightly increased from 15.23 [95% CI: 13.25 to 17.22] to 15.43 [95% CI: 13.35 to 17.50].

For depression, the effect remained associated (MD: 15.51 [95% CI: 13.09 to 17.93]), with minimal impact from study quality or design. Anxiety also remained associated with worse HRQoL (MD: 15.08 [95% CI: 10.56 to 19.61]), with no major differences between cohort (-14.54) and cross-sectional studies (-15.60).

For SMI, after removing low-quality studies, one study left (21.4 [95% CI: 11.46 to 31.34]), and data for cohort studies were unavailable. Mixed mental illnesses remained inconclusive (MD: 11.00 [95% CI: -0.68 to 22.68]), with no changes after sensitivity analysis.

Alsaab S, *et al. BMJ Open Respir Res* 2026; 13:e003977. doi: 10.1136/bmjresp-2025-003977

Supplement, table 5: Sensitivity analysis for SGRQ.

Subgroup	Mean difference [95% CI]	Fair and high quality	Cohort	Cross-sectional
Depression	**MD: 15.40 [13.09, 17.72]**	**MD: 15.51 [13.09, 17.93]**	**MD: 15.94 [12.87, 19.02]**	**MD: 15.04 [11.19, 18.89]**
Anxiety	**MD: 15.08 [10.56, 19.61]**	**MD: 15.08 [10.56, 19.61]**	**MD: 14.54 [11.87, 17.21]**	**MD: 15.60 [7.04, 24.17]**
SMI	**MD: 15.46 [4.30, 26.63]**	**MD: 21.4 [11.46, 31.34]***	NA	**MD: 21.4 [11.46, 31.34]***
Mixed group	MD: 11 [-0.68, 22.68]*	MD: 11 [-0.68, 22.68]*	NA	MD: 11 [-0.68, 22.68]*
**Total**	**MD: 15.23 [13.25, 17.22]**	**MD: 15.43 [13.35, 17.50]**	**MD: 15.44 [13.20, 17.68]**	**MD: 15.42 [12.02, 18.82]**
N of studies	32	29	13	16
I^2	90%	91%	86%	93%

**One study remained in this subgroup.*

mMRC

Sensitivity analysis

A sensitivity analysis was conducted (Supplement, table 6) by excluding poor quality studies and stratifying by study design (cohort and cross-sectional studies). The overall effect remained consistent, confirming that COPD patients with mental illnesses experience higher mMRC scores. After excluding poor quality studies, the total pooled MD remained almost the same 0.65 [95% CI: 0.45 to 0.86], reinforcing the association between the total mental illnesses and mMRC scores. For depression, the effect remained significant across all analyses (MD: 0.89 [95% CI: 0.54 to 1.23] in fair and high-quality studies), with cohort studies showing a wider confidence interval and a non-significant effect (MD: 0.74 [95% CI: -0.31 to 1.80]), while cross-sectional studies remained significant (MD: 0.93 [95% CI: 0.63 to 1.24]).

For anxiety, the association remained significant overall (MD: 0.49 [95% CI: 0.39 to 0.60]) and in fair and high-quality studies, but when stratified, cohort studies showed a consistent effect (MD:

Alsaab S, *et al. BMJ Open Respir Res* 2026; 13:e003977. doi: 10.1136/bmjresp-2025-003977

0.51 [95% CI: 0.41 to 0.61]), whereas cross-sectional studies showed a non-significant effect (MD: 0.20 [95% CI: -0.24 to 0.64]).

For SMI, there was only one study which was not enough to draw a definitive conclusion.

For mixed mental illnesses, the MD remained unchanged at 0.46 [95% CI: 0.30 to 0.63], with no variation after removing poor-quality studies.

Supplement, table 6: Sensitivity analysis for mMRC.

Subgroup	Mean difference [95% CI]	Fair and high quality	Cohort	Cross-sectional
Depression	**MD: 0.83 [0.53, 1.14]**	**MD: 0.89 [0.54, 1.23]**	MD: 0.74 [-0.31, 1.80]	**MD: 0.93 [0.63, 1.24]**
Anxiety	**MD: 0.49 [0.39, 0.60]**	**MD: 0.49 [0.39, 0.60]**	**MD: 0.51 [0.41, 0.61]**	MD: 0.20 [-0.24, 0.64]*
SMI	MD: 0.0 [-0.37, 0.37]*	MD: 0.0 [-0.37, 0.37]*	MD: 0.0 [-0.37, 0.37]*	NA
Mixed group	**MD: 0.46 [0.30, 0.63]**	**MD: 0.46 [0.30, 0.63]**	NA	**MD: 0.46 [0.30, 0.63]**
**Total**	**MD: 0.64 [0.45, 0.84]**	**MD: 0.65 [0.45, 0.86]**	**MD: 0.41 [0.23, 0.59]**	**MD: 0.77 [0.50, 1.03]**
N of studies	17	15	5	10
I^2	89%	91%	74%	88%

**One study remained in this subgroup.*

CAT

Sensitivity analysis

A sensitivity analysis was conducted (Supplement, table 7) by stratifying studies based on design (cohort and cross-sectional) since all the included studies were good quality. The stratified analysis showed that the overall association between mental health conditions and higher CAT scores remained significant in both study designs, with a pooled MD of 6.42 points [95% CI: 5.62 to 7.23] in cohort studies and 7.67 points [95% CI: 5.46 to 9.89] in cross-sectional studies.

Within subgroups, all mental health conditions showed an association with higher CAT scores. The association for SMI couldn't be meta-analysed due to the presence of one study only in that subgroup.

Heterogeneity remained high in cross-sectional studies (I^2 = 82%) but dropped to 0% in cohort studies, suggesting more consistent results in longitudinal designs. Overall, the sensitivity analysis confirmed the primary findings, with mental health comorbidities, particularly depression and anxiety, being consistently associated with higher CAT scores in COPD patients.

Supplement, table 7: Sensitivity analysis for CAT score

Subgroup	Mean difference [95% CI]	Fair and high quality	Cohort	Cross-sectional
Depression	**MD: 7.8 [4.66, 10.93]**	NA	**MD: 6.2 [5.18, 7.22]***	**MD: 9.4 [8.33, 10.47]***
Anxiety	**MD: 6.89 [5.62, 8.16]**	NA	**MD: 6.8 [5.48, 8.12]***	**MD: 8.10 [3.26, 12.94]***
SMI	**MD: 5.8 [1.47, 10.13]***	NA	NA	**MD: 5.8 [1.47, 10.13]***
Mixed group	**MD: 7.17 [3.07, 11.28]**	NA	NA	**MD: 7.17 [3.07, 11.28]**
**Total**	**MD: 7.28 [5.81, 8.74]**	NA	**MD: 6.42 [5.62, 7.23]**	**MD: 7.67 [5.46, 9.89]**
N of studies	7	NA	2	5
I^2	81%	NA	0%	82%

**One study remained in this subgroup.*

Additional figures

Alsaab S, *et al. BMJ Open Respir Res* 2026; 13:e003977. doi: 10.1136/bmjresp-2025-003977

Study or Subgroup	Mental illnesses + COPD Mean [%]	SD [%]	Total	COPD alone Mean [%]	SD [%]	Total	Weight	Mean difference IV, Random, 95% CI [%]	Risk of Bias A
**1.1.1 Depression**									
Chavannes 2005	63.9	15.2	40	63.4	19.7	107	2.1%	0.50 [-5.51 , 6.51]	?
Gudmundsson 2006	36.6	15.9	31	39.6	18.4	56	1.8%	-3.00 [-10.39 , 4.39]	?
Marco 2006	54	18.5	38	54	12.8	164	2.0%	0.00 [-6.20 , 6.20]	?
Fan 2007	27.5	7.3	249	26.8	6.9	361	3.2%	0.70 [-0.45 , 1.85]	+
Ng 2007	47.3	21.8	167	48.2	20.2	209	2.6%	-0.90 [-5.19 , 3.39]	?
Xu 2008	45.7	16.4	112	48.2	15.8	379	2.8%	-2.50 [-5.93 , 0.93]	+
Al-shair 2009	50.4	16.4	29	52.4	19.1	93	1.8%	-2.00 [-9.12 , 5.12]	?
An 2010	42.5	10.8	48	46.2	11	208	2.8%	-3.70 [-7.10 , -0.30]	?
Kil 2010	52.2	17.8	16	59.1	19.4	77	1.3%	-6.90 [-16.64 , 2.84]	-
Hanania 2011	46	16	557	49	16	1561	3.2%	-3.00 [-4.55 , -1.45]	+
Venkata 2012	49	17	16	49	16	20	1.1%	0.00 [-10.89 , 10.89]	-
Horita 2013	38	14	32	51	13	52	2.1%	-13.00 [-19.00 , -7.00]	?
Iguchi 2013	32.9	12.8	36	41.5	20.2	38	1.7%	-8.60 [-16.26 , -0.94]	-
Papaioannou 2013	40.4	10.7	91	60.6	20.7	139	2.6%	-20.20 [-24.28 , -16.12]	+
Kim 2014	60.37	23.65	43	60.57	22.48	202	1.7%	-0.20 [-7.92 , 7.52]	+
Marco 2014	61	11	19	63	15	51	2.0%	-2.00 [-8.44 , 4.44]	?
Miravitlles 2014	51.6	17.5	642	53.6	16.5	217	3.0%	-2.00 [-4.58 , 0.58]	+
Orlandi 2016	51.92	5.31	12	43.55	2.28	42	2.9%	8.37 [5.29 , 11.45]	?
Yohannes 2016	48.4	27.9	377	50.7	18.3	869	2.9%	-2.30 [-5.37 , 0.77]	+
Rivera 2016	38.8	15.9	28	46.2	15.3	87	1.9%	-7.40 [-14.11 , -0.69]	?
Tse 2016	38.7	4.1	18	46.1	2.23	71	3.1%	-7.40 [-9.36 , -5.44]	?
Mi 2017	37.8	26.5	48	38.3	17.2	64	1.5%	-0.50 [-9.10 , 8.10]	?
Yohannes 2017	46.3	30.5	540	48.7	18.2	1519	3.0%	-2.40 [-5.13 , 0.33]	+
Lee 2018	77.7	12.8	30	80.1	13.2	181	2.4%	-2.40 [-7.37 , 2.57]	?
Lim 2019	53.5	17.8	53	55.1	16.4	217	2.3%	-1.60 [-6.87 , 3.67]	?
Vikjord 2020	61.4	17.3	331	65	18.4	1443	3.1%	-3.60 [-5.69 , -1.51]	+
Horner 2023	51	16.53	284	56.9	16	331	3.0%	-5.90 [-8.48 , -3.32]	?
Narod 2023	48	2	51	55	4	44	3.2%	-7.00 [-8.30 , -5.70]	-
Patange 2023	46	3	33	54	4	32	3.2%	-8.00 [-9.72 , -6.28]	-
**Subtotal (Wald[a])**			**3971**			**8834**	**70.2%**	**-3.74 [-5.53 , -1.95]**	
Test for overall effect: Z = 4.09 (P < 0.0001)									
Heterogeneity: Tau² (DL[b]) = 18.08; Chi² = 267.57, df = 28 (P < 0.00001); I² = 90%									
**1.1.2 Anxiety**									
Marco 2006	54	15.1	57	54	24.1	145	2.2%	0.00 [-5.55 , 5.55]	?
Gudmundsson 2006	38.4	19	81	39.6	18.4	146	2.3%	-1.20 [-6.30 , 3.90]	?
Xu 2008	46	15.8	47	47.8	15.9	444	2.4%	-1.80 [-6.55 , 2.95]	+
An 2010	44.2	11.4	32	45.7	11	224	2.6%	-1.50 [-5.70 , 2.70]	?
Mehta 2014	44.5	18.4	11	45.9	16.1	48	1.0%	-1.40 [-13.19 , 10.39]	?
Mi 2017	43.7	22.3	40	34.9	19.2	60	1.7%	8.80 [1.03 , 16.57]	?
Vikjord 2020	64.2	17.4	337	65.1	18.3	1239	3.1%	-0.90 [-3.02 , 1.22]	+
Aldhahi 2023	55.97	14.9	32	56.57	12.14	38	2.0%	-0.60 [-7.05 , 5.85]	+
**Subtotal (Wald[a])**			**649**			**2344**	**17.3%**	**-0.66 [-2.16 , 0.85]**	
Test for overall effect: Z = 0.85 (P = 0.39)									
Heterogeneity: Tau² (DL[b]) = 0.00; Chi² = 6.23, df = 7 (P = 0.51); I² = 0%									
**1.1.3 SMI**									
Laurin 2009	44	20	54	41	15	56	1.9%	3.00 [-3.62 , 9.62]	+
Livermore 2012	46.7	19.7	25	57	22.8	37	1.2%	-10.30 [-20.96 , 0.36]	-
Mehta 2014	46.9	16	19	45	16.8	40	1.5%	1.90 [-6.98 , 10.78]	?
**Subtotal (Wald[a])**			**98**			**133**	**4.6%**	**-0.94 [-8.43 , 6.54]**	
Test for overall effect: Z = 0.25 (P = 0.80)									
Heterogeneity: Tau² (DL[b]) = 24.45; Chi² = 4.55, df = 2 (P = 0.10); I² = 56%									
**1.1.4 Mixed**									
Kuhl 2008	50.3	15.6	28	50	14.8	115	2.0%	0.30 [-6.08 , 6.68]	?
Regvat 2011	42.6	14.4	25	51.1	19.6	25	1.3%	-8.50 [-18.03 , 1.03]	-
Zhang 2014	43.37	16.19	95	48	28.5	264	2.4%	-4.63 [-9.36 , 0.10]	+
Dua 2018	51.6	16.1	40	52.51	16.61	88	2.1%	-0.91 [-6.99 , 5.17]	+
**Subtotal (Wald[a])**			**188**			**492**	**7.9%**	**-2.95 [-6.14 , 0.24]**	
Test for overall effect: Z = 1.81 (P = 0.07)									
Heterogeneity: Tau² (DL[b]) = 0.75; Chi² = 3.22, df = 3 (P = 0.36); I² = 7%									
**Total (Wald[a])**			**4906**			**11803**	**100.0%**	**-2.92 [-4.35 , -1.48]**	
95% prediction interval								[-10.94 , 5.11]	

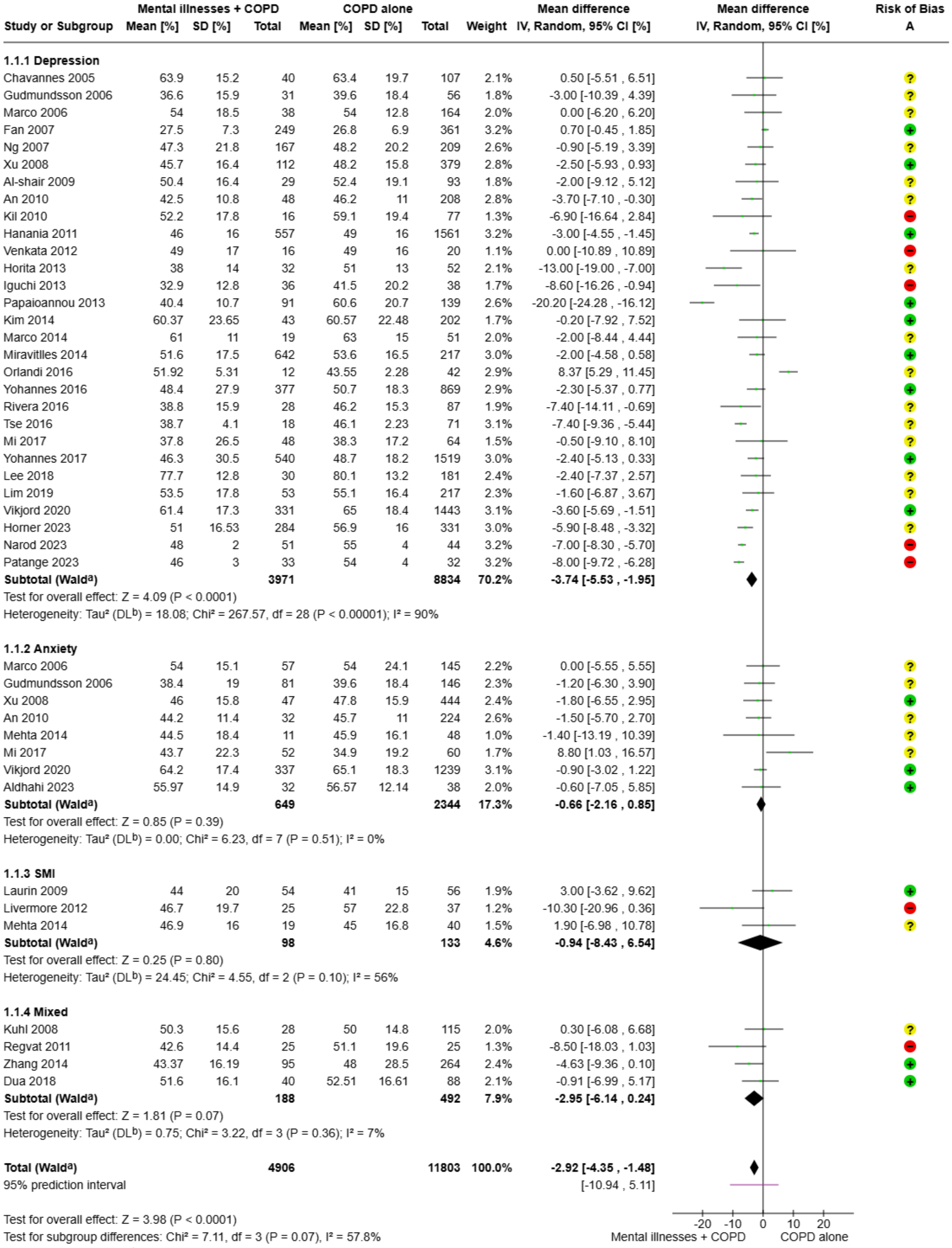

Test for overall effect: Z = 3.98 (P < 0.0001)
Test for subgroup differences: Chi² = 7.11, df = 3 (P = 0.07), I² = 57.8%
Heterogeneity: Tau² (DL[b]) = 16.23; Chi² = 297.28, df = 43 (P < 0.00001); I² = 86%

Footnotes
[a]CI calculated by Wald-type method.
[b]Tau² calculated by DerSimonian and Laird method.

Risk of bias legend
(A) Newcastle-ottwa scale

Supplement, Figure 1:Forest plot of studies presenting data on FEV1% for COPD patients with and without mental illnesses.

	Mental illnesses + COPD			COPD alone				Mean difference	Risk of Bias
**Study or Subgroup**	**Mean [%]**	**SD [%]**	**Total**	**Mean [%]**	**SD [%]**	**Total**	**Weight**	**IV, Random, 95% CI [%]**	**A**
**1.2.1 Depression**									
Marco 2014	66	16	51	70	23	19	2.9%	-4.00 [-15.24 , 7.24]	?
Hong 2023	60.5	20.3	334	64.2	20.9	884	56.0%	-3.70 [-6.28 , -1.12]	+
**Subtotal (Wald[a])**			**385**			**903**	**58.9%**	**-3.71 [-6.23 , -1.20]**	
Test for overall effect: Z = 2.90 (P = 0.004)									
Heterogeneity: Tau² (DL[b]) = 0.00; Chi² = 0.00, df = 1 (P = 0.96); I² = 0%									
**1.2.2 Anxiety**									
Hong 2023	60.1	19.2	198	64	20.2	829	41.1%	-3.90 [-6.91 , -0.89]	+
**Subtotal**			**198**			**829**	**41.1%**	**-3.90 [-6.91 , -0.89]**	
Test for overall effect: Z = 2.54 (P = 0.01)									
Heterogeneity: Not applicable									
**Total (Wald[a])**			**583**			**1732**	**100.0%**	**-3.79 [-5.72 , -1.86]**	
95% prediction interval								[-5.72 , -1.86]	

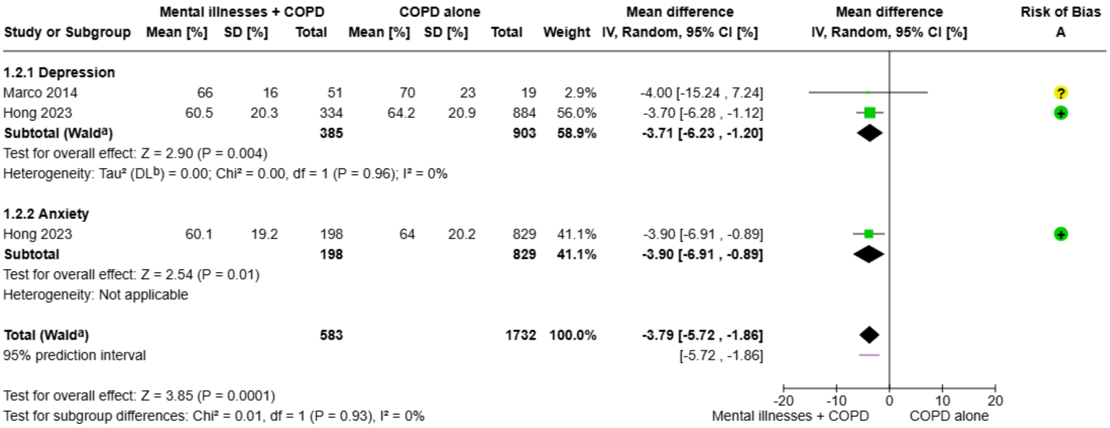

Test for overall effect: Z = 3.85 (P = 0.0001)
Test for subgroup differences: Chi² = 0.01, df = 1 (P = 0.93), I² = 0%
Heterogeneity: Tau² (DL[b]) = 0.00; Chi² = 0.01, df = 2 (P = 0.99); I² = 0%

Footnotes
[a]CI calculated by Wald-type method.
[b]Tau² calculated by DerSimonian and Laird method.

Risk of bias legend
(A) Newcastle-ottwa scale

Supplement, Figure 2: Forest plot of studies presenting raw DLCO data for patients with and without mental illnesses.

Study or Subgroup	Mental illnesses + COPD Mean [meter]	Mental illnesses + COPD SD [meter]	Mental illnesses + COPD Total	COPD alone Mean [meter]	COPD alone SD [meter]	COPD alone Total	Weight	Mean difference IV, Random, 95% CI [meter]	Risk of Bias A
**1.3.1 Depression**									
Fan 2007	349.5	91.8	249	372.3	95.71	361	4.2%	-22.80 [-37.88 , -7.72]	+
Xu 2008	320.5	138.3	112	402.7	129.9	379	3.5%	-82.20 [-110.96 , -53.44]	+
Al-shair 2009	325.6	65.4	29	354.6	73	93	3.5%	-29.00 [-57.05 , -0.95]	?
An 2010	429.2	94.8	48	463.8	80.3	208	3.4%	-34.60 [-63.55 , -5.65]	?
Hanania 2011	331	119	557	382	120	1561	4.4%	-51.00 [-62.54 , -39.46]	+
Lou 2012	401.5	88.5	393	460.6	100.5	707	4.4%	-59.10 [-70.56 , -47.64]	?
Venkata 2012	291	115	16	290	90	20	1.6%	1.00 [-67.78 , 69.78]	−
Horita 2013	232	183	32	477	96	52	1.6%	-245.00 [-313.56 , -176.44]	?
Iguchi 2013	238	102.2	36	335.1	98.3	38	2.5%	-97.10 [-142.83 , -51.37]	−
Kim 2014	320.77	85.74	43	352.43	79.48	202	3.5%	-31.66 [-59.53 , -3.79]	+
Marco 2014	337	85	19	393	92	51	2.5%	-56.00 [-101.81 , -10.19]	?
Rivera 2016	268.4	126.5	28	426.9	106.7	87	2.2%	-158.50 [-210.44 , -106.56]	?
Tse 2016	183.2	28.2	18	234.7	13.4	71	4.3%	-51.50 [-64.90 , -38.10]	?
Yohannes 2016	353	211	377	404	139	869	3.8%	-51.00 [-74.22 , -27.78]	+
Biswas 2017	412.79	90.57	41	430.7	76.29	34	2.9%	-17.91 [-55.67 , 19.85]	?
Yohannes 2017	331.3	233.9	540	383	139	1519	3.9%	-51.70 [-72.63 , -30.77]	+
Hong 2023	380.7	108.9	334	389.4	122.8	884	4.3%	-8.70 [-22.91 , 5.51]	+
Narod 2023	412.75	2.6	51	432.5	4.2	44	4.6%	-19.75 [-21.18 , -18.32]	−
Patange 2023	414.72	2.5	33	433.6	4.3	32	4.6%	-18.88 [-20.60 , -17.16]	−
Bugajski 2023	334.75	97.1	264	360.63	105.64	444	4.2%	-25.88 [-41.17 , -10.59]	?
**Subtotal (Wald[a])**			**3220**			**7656**	**70.0%**	**-40.13 [-46.83 , -33.42]**	
Test for overall effect: Z = 11.74 (P < 0.00001)									
Heterogeneity: Tau² (DL[b]) = 108.98; Chi² = 215.07, df = 19 (P < 0.00001); I² = 91%									
**1.3.2 Anxiety**									
Xu 2008	333	143	47	389.5	134.4	444	2.6%	-56.50 [-99.25 , -13.75]	+
An 2010	406.8	97.7	32	464.6	79.6	224	3.1%	-57.80 [-93.22 , -22.38]	?
Lou 2012	408.5	78.6	201	456.8	95.5	899	4.4%	-48.30 [-60.83 , -35.77]	?
Aldhahi 2023	308.75	129.16	32	373.76	106.56	38	2.0%	-65.01 [-121.14 , -8.88]	+
Bugajski 2023	337.28	94.21	257	360.63	105.64	432	4.2%	-23.35 [-38.58 , -8.12]	?
Hong 2023	377.9	119.8	198	106.2	108.8	829	4.1%	271.70 [253.44 , 289.96]	+
**Subtotal (Wald[a])**			**767**			**2866**	**20.4%**	**4.09 [-115.04 , 123.23]**	
Test for overall effect: Z = 0.07 (P = 0.95)									
Heterogeneity: Tau² (DL[b]) = 21869.49; Chi² = 915.06, df = 5 (P < 0.00001); I² = 99%									
**1.3.3 Mixed**									
Kuhl 2008	298	221	28	297	227	115	1.0%	1.00 [-90.77 , 92.77]	?
Regvat 2011	204	98	25	212.8	131.9	25	1.7%	-8.80 [-73.21 , 55.61]	−
Zhang 2014	431.38	124.34	95	429	112.24	264	3.5%	2.38 [-26.05 , 30.81]	+
Dua 2018	397.1	79.66	40	417.95	70.38	88	3.5%	-20.85 [-49.58 , 7.88]	+
**Subtotal (Wald[a])**			**188**			**492**	**9.7%**	**-8.66 [-27.53 , 10.21]**	
Test for overall effect: Z = 0.90 (P = 0.37)									
Heterogeneity: Tau² (DL[b]) = 0.00; Chi² = 1.31, df = 3 (P = 0.73); I² = 0%									
**Total (Wald[a])**			**4175**			**11014**	**100.0%**	**-30.42 [-41.03 , -19.82]**	
95% prediction interval								[-80.81 , 19.97]	

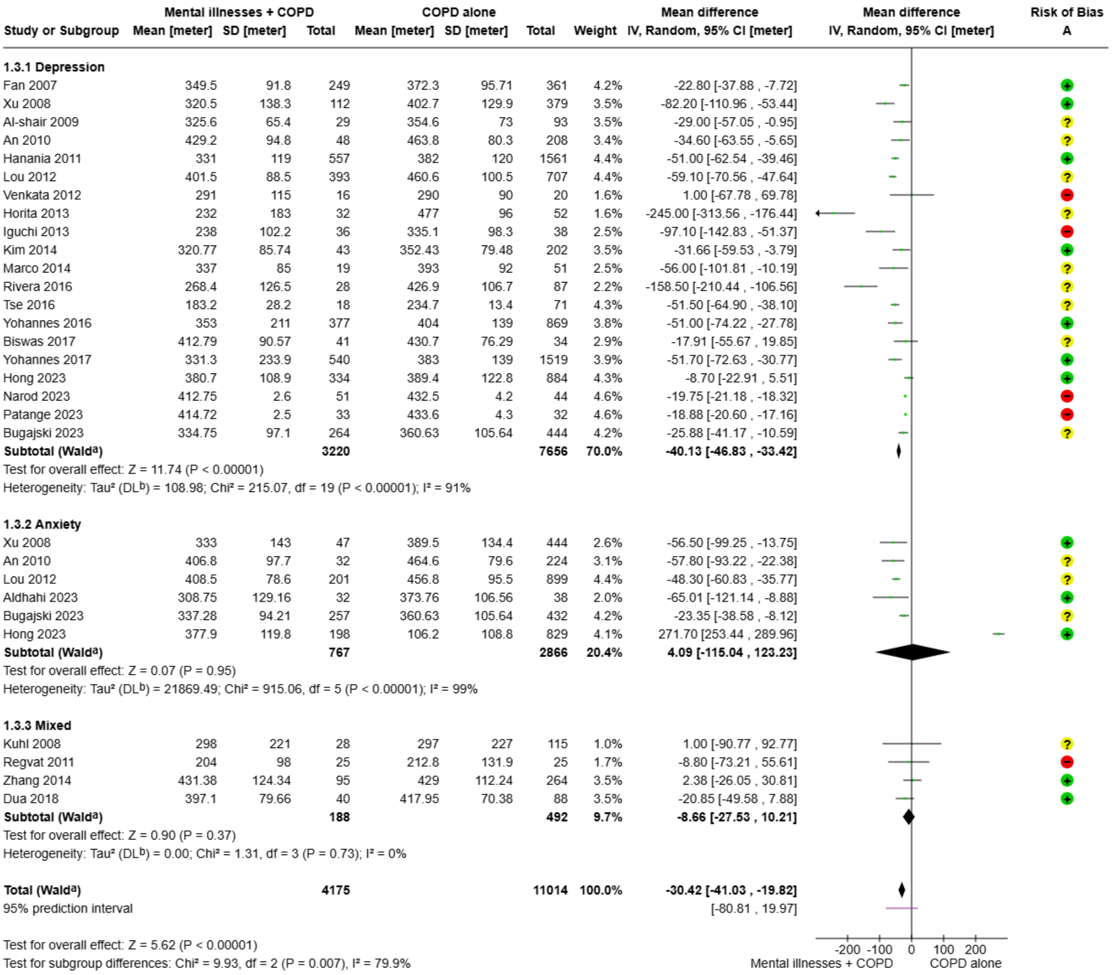

Test for overall effect: Z = 5.62 (P < 0.00001)
Test for subgroup differences: Chi² = 9.93, df = 2 (P = 0.007), I² = 79.9%
Heterogeneity: Tau² (DL[b]) = 631.72; Chi² = 1229.35, df = 29 (P < 0.00001); I² = 98%

Footnotes
[a]CI calculated by Wald-type method.
[b]Tau² calculated by DerSimonian and Laird method.

Risk of bias legend
(A) Newcastle-ottwa scale

Supplement, Figure 3: Forest plot of studies presenting raw 6MWT data for patients with and without mental illnesses.

Alsaab S, *et al. BMJ Open Respir Res* 2026; 13:e003977. doi: 10.1136/bmjresp-2025-003977

Study or Subgroup	Mental illnesses + COPD Mean [point]	SD [point]	Total	COPD alone Mean [point]	SD [point]	Total	Weight	Mean difference IV, Random, 95% CI [point]	Risk of Bias A
**1.4.1 Depression**									
Gudmundsson 2006	64.1	14	31	52.3	17.8	56	2.8%	11.80 [5.02 , 18.58]	?
Marco 2006	56.4	21	38	42.4	23	164	2.6%	14.00 [6.45 , 21.55]	?
Fan 2007	59	11.8	249	49.9	11.9	361	4.0%	9.10 [7.19 , 11.01]	+
Ng 2007	51.9	15.3	167	38.8	14.1	209	3.8%	13.10 [10.09 , 16.11]	?
Xu 2008	62.3	17.5	112	41.6	16.6	379	3.6%	20.70 [17.05 , 24.35]	+
Al-shair 2009	63.8	16.1	29	41.1	18.3	93	2.7%	22.70 [15.76 , 29.64]	?
An 2010	51.8	15.2	48	37.4	15.8	208	3.3%	14.40 [9.59 , 19.21]	?
Kil 2010	52.5	23.2	16	38.9	18.7	77	1.6%	13.60 [1.49 , 25.71]	−
Hanania 2011	64	17	557	45	19	1561	4.0%	19.00 [17.30 , 20.70]	+
Ito 2012	56.1	23	14	29.4	16.4	46	1.5%	26.70 [13.75 , 39.65]	+
Lou 2012	55.3	16.2	393	40.4	17.8	707	3.9%	14.90 [12.83 , 16.97]	?
Iguchi 2013	60.8	15.7	36	46.7	16.9	38	2.6%	14.10 [6.67 , 21.53]	−
Marco 2014	44.3	9.9	19	25.2	11.1	51	3.2%	19.10 [13.71 , 24.49]	?
Tse 2016	57	4.5	18	49.2	2.5	71	3.9%	7.80 [5.64 , 9.96]	?
Yohannes 2016	56.4	30.4	377	39.9	20	869	3.7%	16.50 [13.16 , 19.84]	+
Yohannes 2017	61	32.3	540	43.5	19.3	1519	3.8%	17.50 [14.61 , 20.39]	+
Bugajski 2023	60.9	10.5	264	51.7	12.8	444	4.0%	9.20 [7.46 , 10.94]	?
Hong 2023	41.2	20.1	334	25.7	15.9	884	3.9%	15.50 [13.10 , 17.90]	+
Horner 2023	50.43	20.47	284	29.3	16.2	331	3.8%	21.13 [18.18 , 24.08]	?
**Subtotal (Wald[a])**			**3526**			**8068**	**62.8%**	**15.40 [13.09 , 17.72]**	
Test for overall effect: Z = 13.03 (P < 0.00001)									
Heterogeneity: Tau² (DL[b]) = 20.59; Chi² = 178.80, df = 18 (P < 0.00001); I² = 90%									
**1.4.2 Anxiety**									
Gudmundsson 2006	61.8	15	81	52.3	17.8	146	3.4%	9.50 [5.14 , 13.86]	?
Marco 2006	59.6	19.6	57	39.3	22.9	145	2.9%	20.30 [13.99 , 26.61]	?
Xu 2008	61.3	18.7	47	44.1	17.5	444	3.1%	17.20 [11.61 , 22.79]	+
An 2010	57.5	14.3	32	37.6	15.4	224	3.2%	19.90 [14.55 , 25.25]	?
Lou 2012	61.5	17.3	201	40.6	16.4	899	3.8%	20.90 [18.28 , 23.52]	?
Mehta 2014	56	23.6	11	42.1	19.9	48	1.2%	13.90 [-1.14 , 28.94]	?
Aldhahi 2023	56.54	22.36	32	42.9	24.76	38	1.8%	13.64 [2.59 , 24.69]	+
Bugajski 2023	56.3	12.2	257	51.7	12.8	432	4.0%	4.60 [2.68 , 6.52]	?
Hong 2023	41.1	20.7	198	25.2	16.1	829	3.8%	15.90 [12.82 , 18.98]	+
Yohannes 2024	63.07	15.49	348	47.84	16.77	645	3.9%	15.23 [13.15 , 17.31]	?
**Subtotal (Wald[a])**			**1264**			**3850**	**31.2%**	**15.08 [10.56 , 19.61]**	
Test for overall effect: Z = 6.54 (P < 0.00001)									
Heterogeneity: Tau² (DL[b]) = 44.08; Chi² = 132.52, df = 9 (P < 0.00001); I² = 93%									
**1.4.3 SMI**									
Livermore 2012	53.5	18.7	25	43.5	15.3	37	2.3%	10.00 [1.17 , 18.83]	−
Mehta 2014	59.2	17.7	19	37.8	19.2	40	2.0%	21.40 [11.46 , 31.34]	?
**Subtotal (Wald[a])**			**44**			**77**	**4.3%**	**15.46 [4.30 , 26.63]**	
Test for overall effect: Z = 2.72 (P = 0.007)									
Heterogeneity: Tau² (DL[b]) = 41.97; Chi² = 2.82, df = 1 (P = 0.09); I² = 65%									
**1.4.4 Mixed**									
Kuhl 2008	64.8	29.1	28	53.8	24.6	115	1.7%	11.00 [-0.68 , 22.68]	?
**Subtotal**			**28**			**115**	**1.7%**	**11.00 [-0.68 , 22.68]**	
Test for overall effect: Z = 1.85 (P = 0.06)									
Heterogeneity: Not applicable									
**Total (Wald[a])**			**4862**			**12110**	**100.0%**	**15.23 [13.25 , 17.22]**	
95% prediction interval								[5.25 , 25.22]	

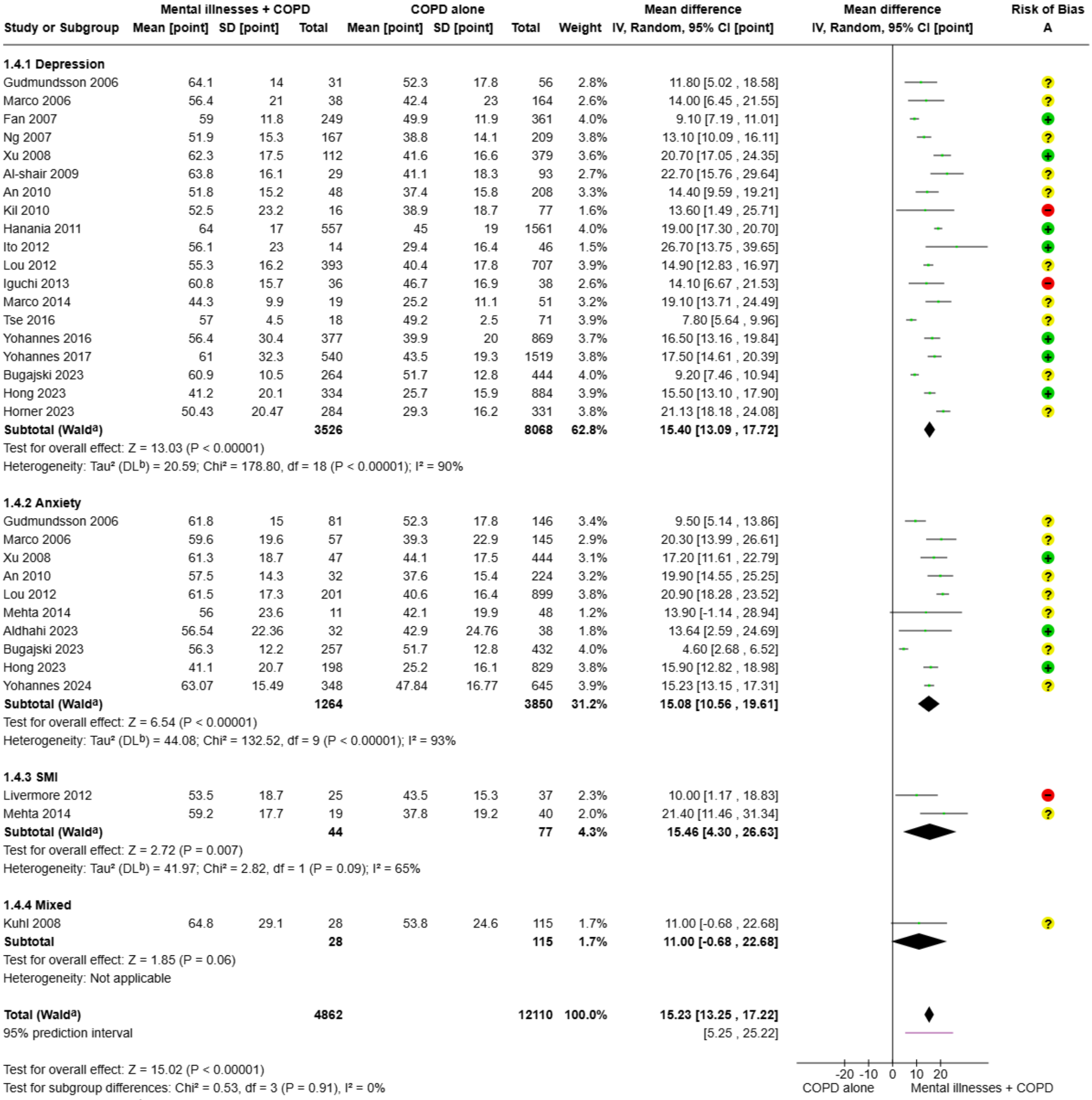

Test for overall effect: Z = 15.02 (P < 0.00001)
Test for subgroup differences: Chi² = 0.53, df = 3 (P = 0.91), I² = 0%
Heterogeneity: Tau² (DL[b]) = 24.93; Chi² = 317.06, df = 31 (P < 0.00001); I² = 90%

Footnotes
[a]CI calculated by Wald-type method.
[b]Tau² calculated by DerSimonian and Laird method.

Risk of bias legend
(A) Newcastle-ottwa scale

Supplement, Figure 4: Forest plot of studies presenting raw SGRQ data for COPD patients with and without mental illnesses.

Alsaab S, *et al. BMJ Open Respir Res* 2026; 13:e003977. doi: 10.1136/bmjresp-2025-003977

	Mental illnesses + COPD			COPD alone				Mean difference	Risk of Bias
**Study or Subgroup**	**Mean [point]**	**SD [point]**	**Total**	**Mean [point]**	**SD [point]**	**Total**	**Weight**	**IV, Random, 95% CI [point]**	**A**
**1.5.1 Depression**									
Chavannes 2005	1.3	0.7	40	0.9	0.9	107	6.7%	0.40 [0.12 , 0.68]	?
Marco 2006	2.6	1.85	38	1.7	1.28	164	4.3%	0.90 [0.28 , 1.52]	?
Venkata 2012	2.5	1.26	16	1.85	0.75	20	3.8%	0.65 [-0.05 , 1.35]	−
Ito 2012	2	1.7	14	0.6	1.2	46	2.6%	1.40 [0.44 , 2.36]	+
Horita 2013	2.5	1.3	32	0.8	0.9	52	5.0%	1.70 [1.19 , 2.21]	?
Iguchi 2013	2.9	1.1	36	2.4	1.1	38	5.0%	0.50 [-0.00 , 1.00]	−
Kim 2014	2.26	1.18	43	1.58	1.12	202	5.9%	0.68 [0.29 , 1.07]	+
Marco 2014	1.8	0.6	19	1.2	0.8	51	6.2%	0.60 [0.25 , 0.95]	?
Rivera 2016	2.7	0.97	28	1.4	0.84	87	5.8%	1.30 [0.90 , 1.70]	?
Tse 2016	2.78	0.24	18	1.68	0.13	71	7.5%	1.10 [0.99 , 1.21]	?
Hong 2023	1.5	1	334	1.2	0.8	884	7.5%	0.30 [0.18 , 0.42]	+
**Subtotal (Wald[a])**			**618**			**1722**	**60.2%**	**0.83 [0.53 , 1.14]**	
Test for overall effect: Z = 5.37 (P < 0.00001)									
Heterogeneity: Tau² (DL[b]) = 0.21; Chi² = 120.22, df = 10 (P < 0.00001); I² = 92%									
**1.5.2 Anxiety**									
Marco 2006	2	1.51	57	1.8	1.2	145	5.5%	0.20 [-0.24 , 0.64]	?
Hong 2023	1.6	1	198	1.1	0.8	829	7.4%	0.50 [0.35 , 0.65]	+
Yohannes 2024	3.3	1.13	348	2.78	1.11	645	7.4%	0.52 [0.37 , 0.67]	?
**Subtotal (Wald[a])**			**603**			**1619**	**20.3%**	**0.49 [0.39 , 0.60]**	
Test for overall effect: Z = 9.50 (P < 0.00001)									
Heterogeneity: Tau² (DL[b]) = 0.00; Chi² = 1.86, df = 2 (P = 0.39); I² = 0%									
**1.5.3 SMI**									
Laurin 2009	2	1	54	2	1	56	6.0%	0.00 [-0.37 , 0.37]	+
**Subtotal**			**54**			**56**	**6.0%**	**0.00 [-0.37 , 0.37]**	
Test for overall effect: Z = 0.00 (P = 1.00)									
Heterogeneity: Not applicable									
**1.5.4 Mixed**									
Zhang 2014	1.86	0.86	95	1.42	0.82	264	7.1%	0.44 [0.24 , 0.64]	+
Dua 2018	2.2	0.78	40	1.68	0.89	88	6.5%	0.52 [0.22 , 0.82]	+
**Subtotal (Wald[a])**			**135**			**352**	**13.6%**	**0.46 [0.30 , 0.63]**	
Test for overall effect: Z = 5.45 (P < 0.00001)									
Heterogeneity: Tau² (DL[b]) = 0.00; Chi² = 0.19, df = 1 (P = 0.67); I² = 0%									
**Total (Wald[a])**			**1410**			**3749**	**100.0%**	**0.64 [0.45 , 0.84]**	
95% prediction interval								[-0.07 , 1.36]	

Test for overall effect: Z = 6.61 (P < 0.00001)
Test for subgroup differences: Chi² = 11.58, df = 3 (P = 0.009), I² = 74.1%
Heterogeneity: Tau² (DL[b]) = 0.12; Chi² = 150.71, df = 16 (P < 0.00001); I² = 89%

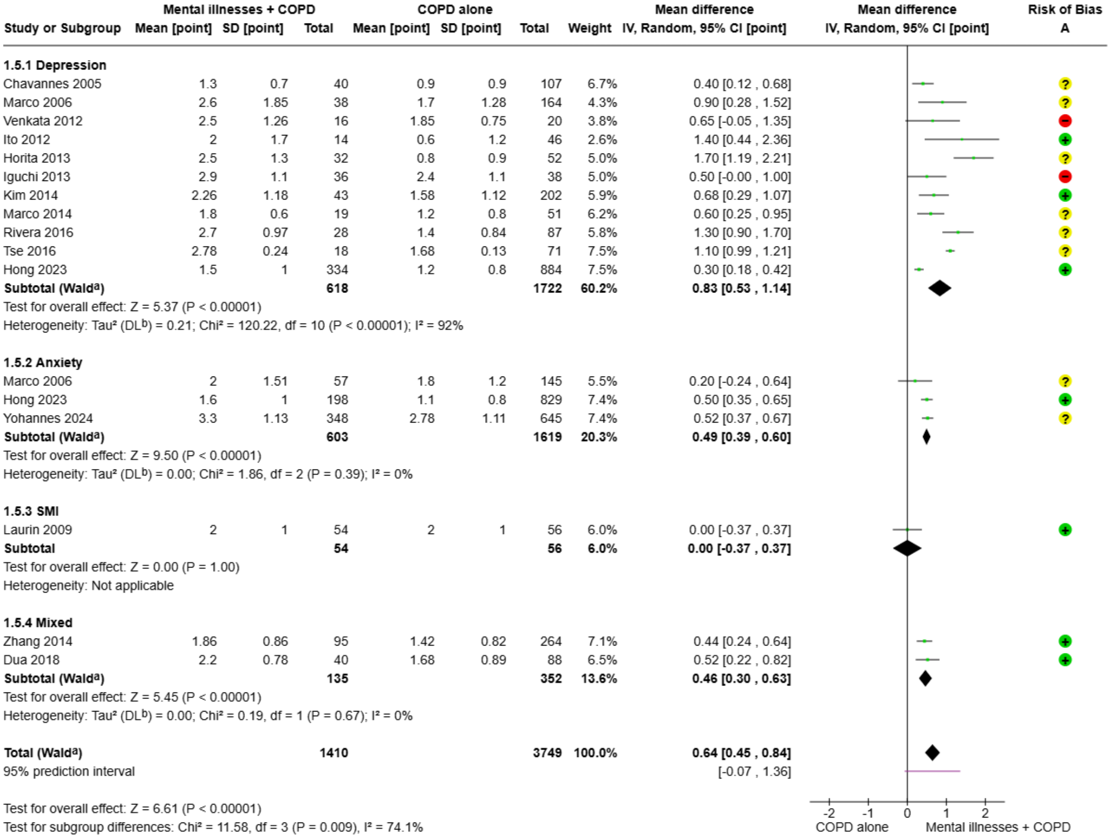

Footnotes
[a]CI calculated by Wald-type method.
[b]Tau² calculated by DerSimonian and Laird method.

Risk of bias legend
(A) Newcastle-ottwa scale

Supplement, Figure 5: Forest plot of studies presenting raw mMRC data for COPD patients with and without mental illnesses.

Study or Subgroup	Mental illnesses + COPD Mean [point]	SD [point]	Total	COPD alone Mean [point]	SD [point]	Total	Weight	Mean difference IV, Random, 95% CI [point]	Risk of Bias A
**1.6.1 Depression**									
Miravitlles 2014	23.9	6.8	642	14.5	7	217	18.4%	9.40 [8.33 , 10.47]	+
Hong 2023	18.3	8.4	334	12.1	7.1	884	18.6%	6.20 [5.18 , 7.22]	+
**Subtotal (Wald[a])**			**976**			**1101**	**37.0%**	**7.80 [4.66 , 10.93]**	
Test for overall effect: Z = 4.87 (P < 0.00001)									
Heterogeneity: Tau² (DL[b]) = 4.84; Chi² = 18.09, df = 1 (P < 0.0001); I² = 94%									
**1.6.2 Anxiety**									
Mehta 2014	21.1	7.5	11	13	6.9	48	6.3%	8.10 [3.26 , 12.94]	?
Hong 2023	18.8	8.8	198	12	7.1	829	17.5%	6.80 [5.48 , 8.12]	+
**Subtotal (Wald[a])**			**209**			**877**	**23.8%**	**6.89 [5.62 , 8.16]**	
Test for overall effect: Z = 10.62 (P < 0.00001)									
Heterogeneity: Tau² (DL[b]) = 0.00; Chi² = 0.26, df = 1 (P = 0.61); I² = 0%									
**1.6.3 SMI**									
Mehta 2014	18.4	8.5	19	12.6	6.6	40	7.3%	5.80 [1.47 , 10.13]	?
**Subtotal**			**19**			**40**	**7.3%**	**5.80 [1.47 , 10.13]**	
Test for overall effect: Z = 2.62 (P = 0.009)									
Heterogeneity: Not applicable									
**1.6.4 Mixed**									
Zhang 2014	20.17	6.5	95	15.06	6.97	264	16.6%	5.11 [3.56 , 6.66]	+
Long 2020	24.5	8	195	15.2	8.1	112	15.3%	9.30 [7.43 , 11.17]	?
**Subtotal (Wald[a])**			**290**			**376**	**31.9%**	**7.17 [3.07 , 11.28]**	
Test for overall effect: Z = 3.42 (P = 0.0006)									
Heterogeneity: Tau² (DL[b]) = 8.01; Chi² = 11.38, df = 1 (P = 0.0007); I² = 91%									
**Total (Wald[a])**			**1494**			**2394**	**100.0%**	**7.28 [5.81 , 8.74]**	
95% prediction interval								[3.71 , 10.84]	

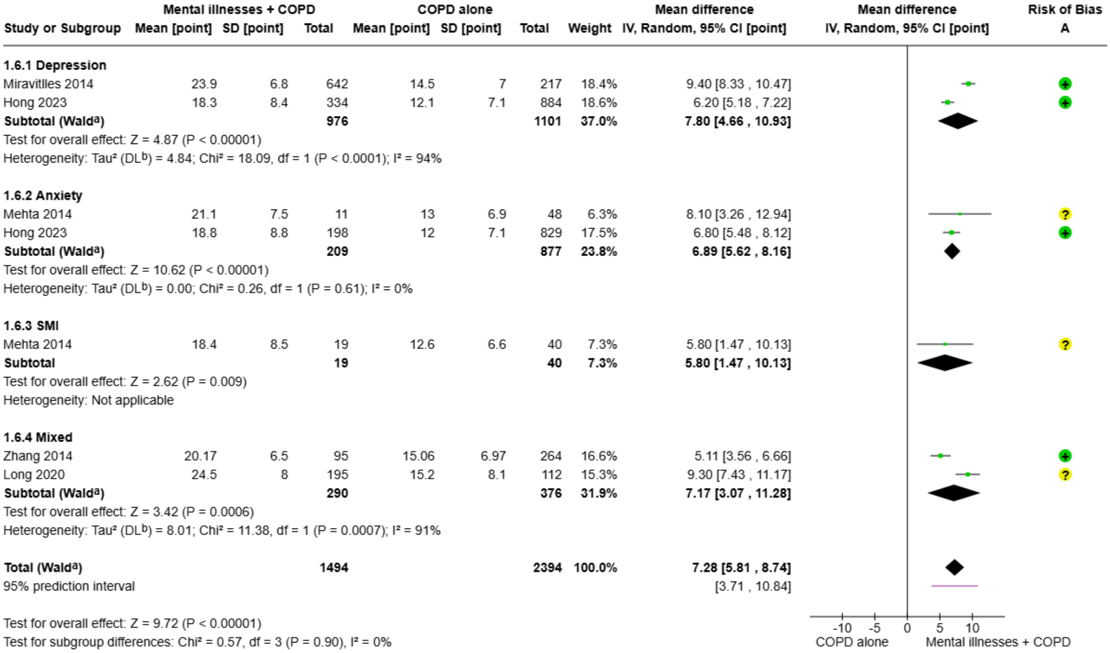

Test for overall effect: Z = 9.72 (P < 0.00001)
Test for subgroup differences: Chi² = 0.57, df = 3 (P = 0.90), I² = 0%
Heterogeneity: Tau² (DL[b]) = 2.75; Chi² = 32.43, df = 6 (P < 0.0001); I² = 81%

Footnotes
[a]CI calculated by Wald-type method.
[b]Tau² calculated by DerSimonian and Laird method.

Risk of bias legend
(A) Newcastle-ottwa scale

Supplement, Figure 6: Forest plot of studies presenting raw CAT data for COPD patients with and without mental illnesses.

Alsaab S, *et al. BMJ Open Respir Res* 2026; 13:e003977. doi: 10.1136/bmjresp-2025-003977

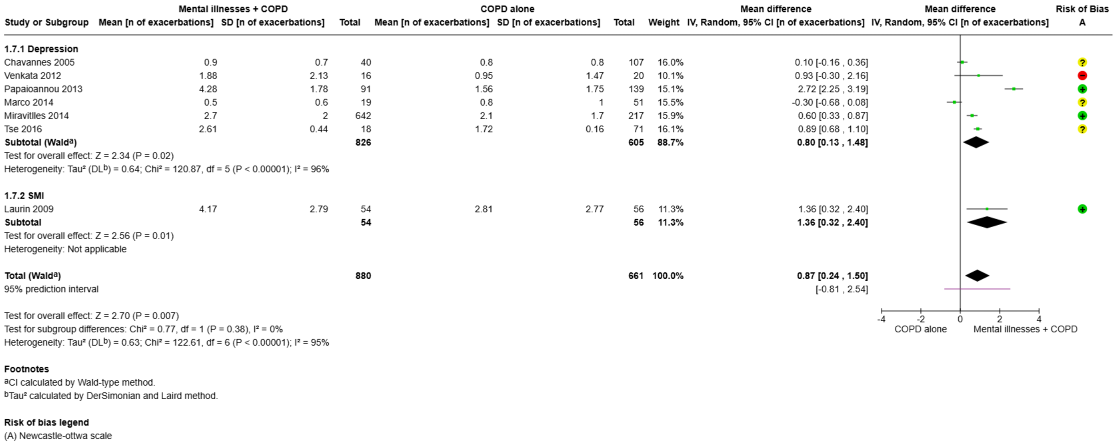

Supplement, Figure 7 (a): Forest plot of studies presenting mean number of exacerbations per year for COPD patients with and without mental illnesses.

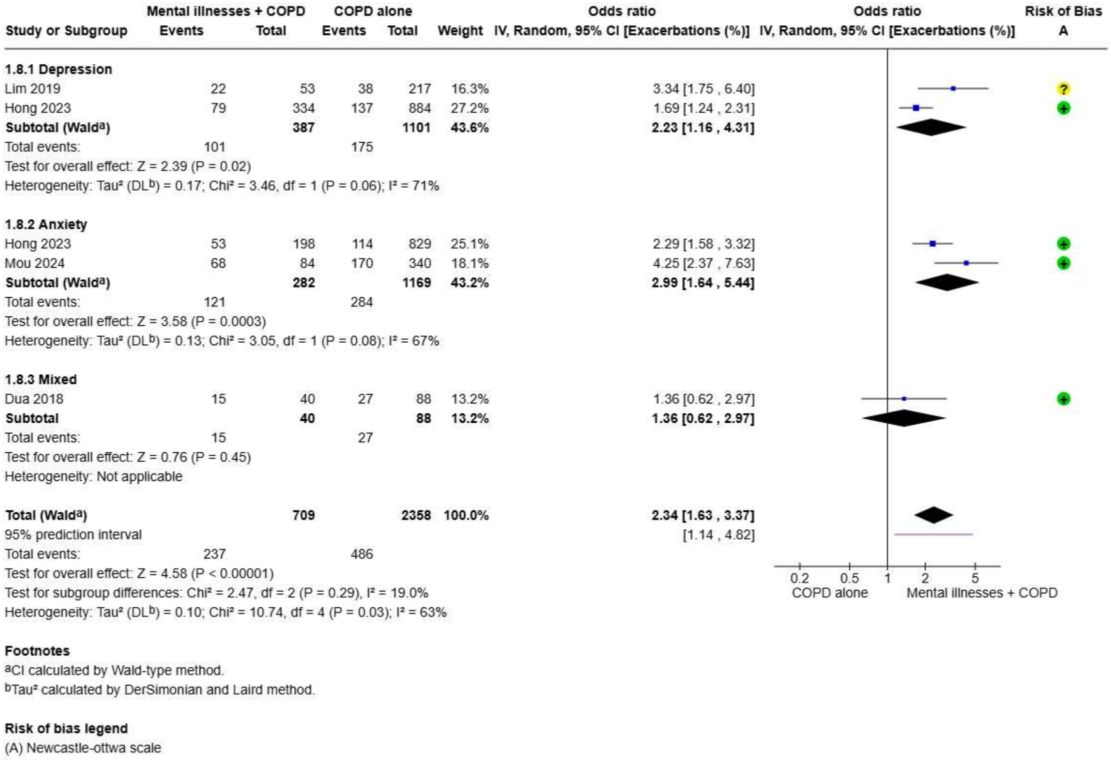

Supplement, Figure 7 (b): Forest plot of studies presenting unadjusted odds of any exacerbations last year for COPD patients with and without mental illnesses.

Alsaab S, *et al. BMJ Open Respir Res* 2026; 13:e003977. doi: 10.1136/bmjresp-2025-003977

Study or Subgroup	Mental illnesses + COPD Events	Mental illnesses + COPD Total	COPD alone Events	COPD alone Total	Weight	Odds ratio IV, Random, 95% CI [Exacerbations (%)]	Risk of Bias A
**1.9.1 Depression**							
Xu 2008	51	112	145	379	23.7%	1.35 [0.88 , 2.07]	+
Horner 2023	71	284	33	331	23.0%	3.01 [1.92 , 4.72]	?
**Subtotal (Wald[a])**		**396**		**710**	**46.7%**	**2.01 [0.91 , 4.41]**	
Total events:	122		178				
Test for overall effect: Z = 1.74 (P = 0.08)							
Heterogeneity: Tau² (DL[b]) = 0.27; Chi² = 6.46, df = 1 (P = 0.01); I² = 85%							
**1.9.2 Anxiety**							
Xu 2008	20	47	176	444	18.8%	1.13 [0.61 , 2.07]	+
Mehta 2014	9	11	19	48	5.3%	6.87 [1.34 , 35.33]	?
**Subtotal (Wald[a])**		**58**		**492**	**24.1%**	**2.36 [0.41 , 13.43]**	
Total events:	29		195				
Test for overall effect: Z = 0.97 (P = 0.33)							
Heterogeneity: Tau² (DL[b]) = 1.23; Chi² = 4.11, df = 1 (P = 0.04); I² = 76%							
**1.9.3 SMI**							
Mehta 2014	10	19	18	40	9.8%	1.36 [0.45 , 4.06]	?
**Subtotal**		**19**		**40**	**9.8%**	**1.36 [0.45 , 4.06]**	
Total events:	10		18				
Test for overall effect: Z = 0.55 (P = 0.58)							
Heterogeneity: Not applicable							
**1.9.4 Mixed**							
Gonzalez-Gutierrez 2016	39	74	45	130	19.4%	2.10 [1.18 , 3.77]	?
**Subtotal**		**74**		**130**	**19.4%**	**2.10 [1.18 , 3.77]**	
Total events:	39		45				
Test for overall effect: Z = 2.51 (P = 0.01)							
Heterogeneity: Not applicable							
**Total (Wald[a])**		**547**		**1372**	**100.0%**	**1.87 [1.23 , 2.82]**	
95% prediction interval						[0.80 , 4.34]	
Total events:	200		436				
Test for overall effect: Z = 2.96 (P = 0.003)							
Test for subgroup differences: Chi² = 0.54, df = 3 (P = 0.91), I² = 0%							
Heterogeneity: Tau² (DL[b]) = 0.14; Chi² = 12.13, df = 5 (P = 0.03); I² = 59%							

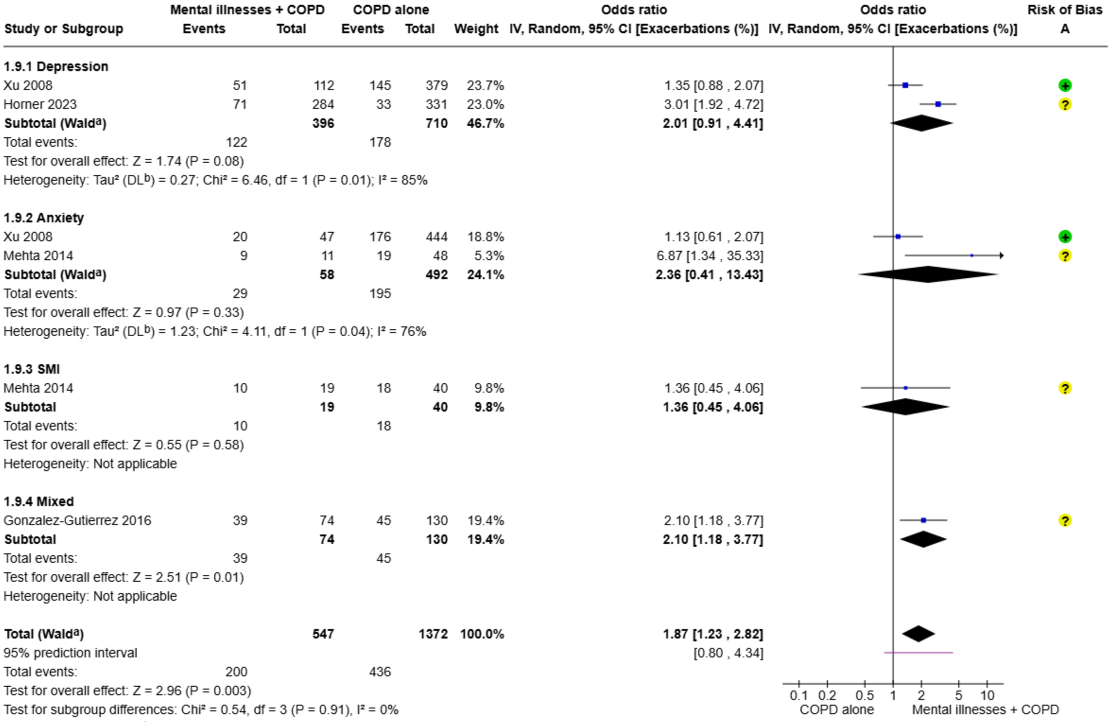

Footnotes
[a]CI calculated by Wald-type method.
[b]Tau² calculated by DerSimonian and Laird method.

Risk of bias legend
(A) Newcastle-ottwa scale

Supplement, Figure 7 (c): Forest plot of studies presenting unadjusted odds of frequent exacerbations (≥2/year) for COPD patients with and without mental illnesses.

	Mental illnesses + COPD			COPD alone				Mean difference	Risk of Bias
Study or Subgroup	Mean [n of admissions]	SD [n of admissions]	Total	Mean [n of admissions]	SD [n of admissions]	Total	Weight	IV, Random, 95% CI [n of admissions]	A
**1.10.1 Depression**									
Ng 2007	2.3	3.1	167	2	2.8	209	15.6%	0.30 [-0.30 , 0.90]	?
Papaioannou 2013	1.96	1.41	91	0.41	0.86	139	18.1%	1.55 [1.23 , 1.87]	+
Miravitlles 2014	1.5	2.2	642	0.9	1.6	217	18.5%	0.60 [0.33 , 0.87]	+
Rivera 2016	1.32	1.8	28	0.7	1.4	87	14.3%	0.62 [-0.11 , 1.35]	?
Tse 2016	2.72	0.5	18	2.59	0.36	71	18.7%	0.13 [-0.12 , 0.38]	?
**Subtotal (Wald[a])**			**946**			**723**	**85.2%**	**0.65 [0.08 , 1.21]**	
Test for overall effect: Z = 2.24 (P = 0.02)									
Heterogeneity: Tau² (DL[b]) = 0.36; Chi² = 48.20, df = 4 (P < 0.00001); I² = 92%									
**1.10.2 Mixed**									
Regvat 2011	1.7	0.9	25	2.1	1.5	25	14.8%	-0.40 [-1.09 , 0.29]	−
**Subtotal**			**25**			**25**	**14.8%**	**-0.40 [-1.09 , 0.29]**	
Test for overall effect: Z = 1.14 (P = 0.25)									
Heterogeneity: Not applicable									
**Total (Wald[a])**			**971**			**748**	**100.0%**	**0.49 [-0.05 , 1.03]**	
95% prediction interval								[-0.84 , 1.83]	

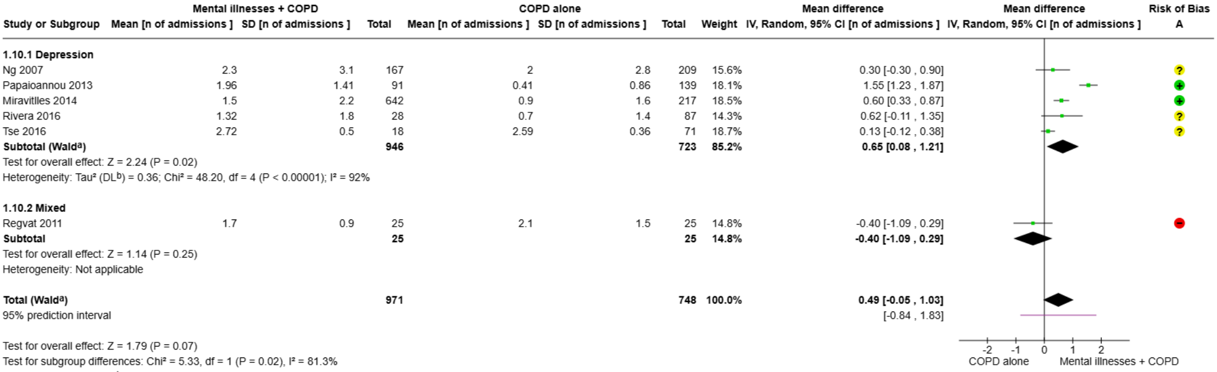

Test for overall effect: Z = 1.79 (P = 0.07)
Test for subgroup differences: Chi² = 5.33, df = 1 (P = 0.02), I² = 81.3%
Heterogeneity: Tau² (DL[b]) = 0.39; Chi² = 56.19, df = 5 (P < 0.00001); I² = 91%

Footnotes
[a]CI calculated by Wald-type method.
[b]Tau² calculated by DerSimonian and Laird method.

Risk of bias legend
(A) Newcastle-ottwa scale

Supplement, Figure 8 (a): Forest plot of studies presenting number of hospitalisations per year for COPD patients with and without mental illnesses.

Study or Subgroup	Mental illnesses + COPD Events	Mental illnesses + COPD Total	COPD alone Events	COPD alone Total	Weight	Odds ratio IV, Random, 95% CI [%]	Risk of Bias A
**1.11.1 Depression**							
Xu 2008	64	112	173	379	8.3%	1.59 [1.04 , 2.43]	+
Abrams 2011	319	3077	2723	21726	10.7%	0.81 [0.71 , 0.91]	+
Papaioannou 2013	77	91	39	139	6.0%	14.10 [7.15 , 27.81]	+
Lim 2019	12	53	9	217	4.3%	6.76 [2.68 , 17.09]	?
Feng 2022	1511	2911	151169	374213	10.9%	1.59 [1.48 , 1.71]	+
**Subtotal (Wald[a])**		**6244**		**396674**	**40.2%**	**2.44 [1.41 , 4.22]**	
Total events:	1983		154113				
Test for overall effect: Z = 3.19 (P = 0.001)							
Heterogeneity: Tau² (DL[b]) = 0.33; Chi² = 144.90, df = 4 (P < 0.00001); I² = 97%							
**1.11.2 Anxiety**							
Xu 2008	26	47	211	444	6.6%	1.37 [0.75 , 2.50]	+
Abrams 2011	201	1784	2841	21726	10.6%	0.84 [0.73 , 0.98]	+
Feng 2022	1636	3288	151169	374213	10.9%	1.46 [1.36 , 1.57]	+
**Subtotal (Wald[a])**		**5119**		**396383**	**28.1%**	**1.17 [0.74 , 1.84]**	
Total events:	1863		154221				
Test for overall effect: Z = 0.68 (P = 0.50)							
Heterogeneity: Tau² (DL[b]) = 0.13; Chi² = 41.61, df = 2 (P < 0.00001); I² = 95%							
**1.11.3 SMI**							
Abrams 2011	135	1569	2907	21726	10.4%	0.61 [0.51 , 0.73]	+
Jorgensen 2018	216	1502	22509	210366	10.6%	1.40 [1.21 , 1.62]	+
**Subtotal (Wald[a])**		**3071**		**232092**	**21.0%**	**0.93 [0.41 , 2.09]**	
Total events:	351		25416				
Test for overall effect: Z = 0.18 (P = 0.85)							
Heterogeneity: Tau² (DL[b]) = 0.34; Chi² = 49.70, df = 1 (P < 0.00001); I² = 98%							
**1.11.4 Mixed**							
Dua 2018	7	40	11	88	3.8%	1.48 [0.53 , 4.17]	+
Montserrat-Capdevila 2018	21	80	58	432	6.9%	2.30 [1.30 , 4.06]	+
**Subtotal (Wald[a])**		**120**		**520**	**10.7%**	**2.07 [1.26 , 3.41]**	
Total events:	28		69				
Test for overall effect: Z = 2.87 (P = 0.004)							
Heterogeneity: Tau² (DL[b]) = 0.00; Chi² = 0.52, df = 1 (P = 0.47); I² = 0%							
**Total (Wald[a])**		**14554**		**1025669**	**100.0%**	**1.50 [1.18 , 1.93]**	
95% prediction interval						[0.69 , 3.29]	
Total events:	4225		333819				
Test for overall effect: Z = 3.24 (P = 0.001)							
Test for subgroup differences: Chi² = 6.87, df = 3 (P = 0.08), I² = 56.4%							
Heterogeneity: Tau² (DL[b]) = 0.14; Chi² = 262.57, df = 11 (P < 0.00001); I² = 96%							

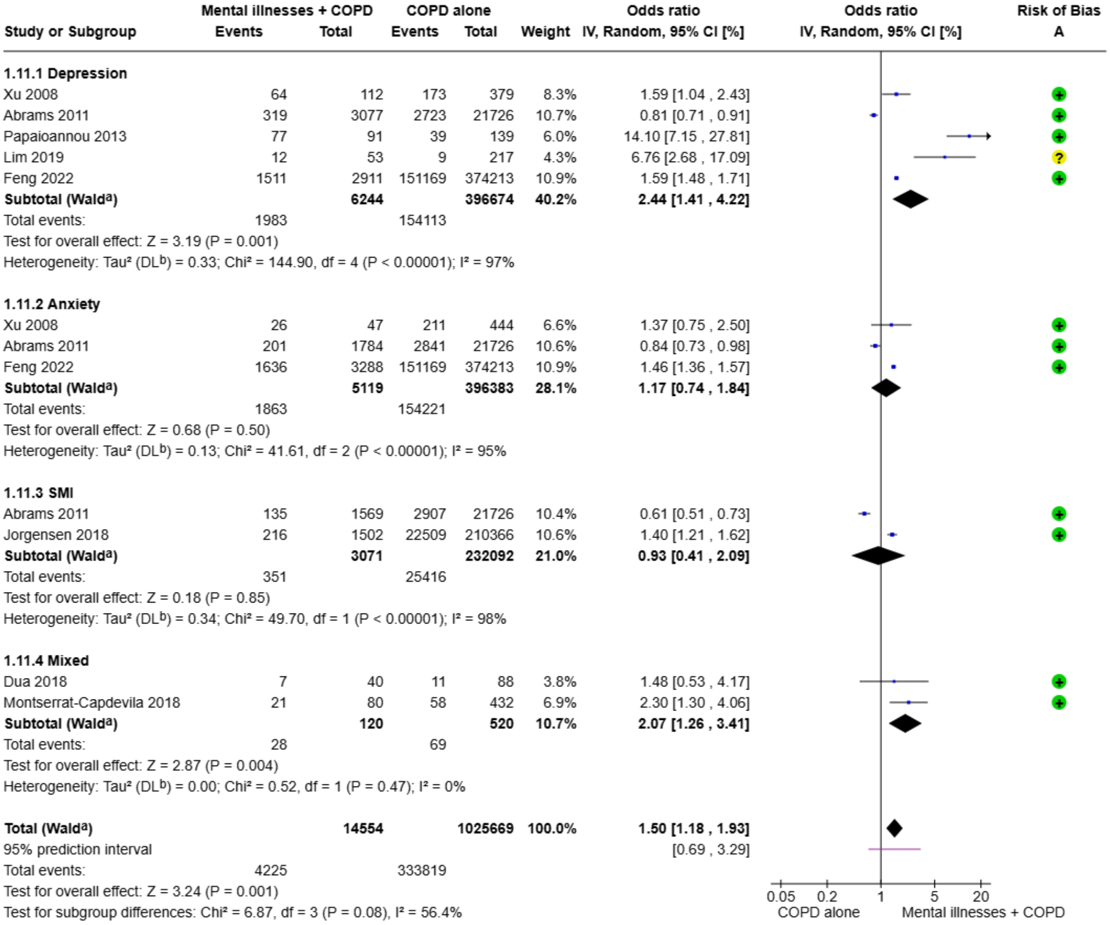

Footnotes
[a]CI calculated by Wald-type method.
[b]Tau² calculated by DerSimonian and Laird method.

Risk of bias legend
(A) Newcastle-ottwa scale

Supplement, Figure 8 (b): Forest plot of studies presenting unadjusted odds of hospitalisations per year for COPD patients with and without mental illnesses.

Study or Subgroup	Mental illnesses + COPD Events	Mental illnesses + COPD Total	COPD alone Events	COPD alone Total	Weight	Odds ratio IV, Random, 95% CI [%]	Risk of Bias A
**1.12.1 Depression**							
Fan 2007	24	249	21	361	8.3%	1.73 [0.94 , 3.18]	+
Ng 2007	35	167	22	209	8.4%	2.25 [1.26 , 4.02]	?
Xu 2008	9	112	7	379	7.9%	4.64 [1.69 , 12.77]	+
Abrams 2011	881	3077	163	21726	8.6%	53.07 [44.65 , 63.08]	+
Papaioannou 2013	23	91	5	139	7.9%	9.06 [3.30 , 24.89]	+
Feng 2022	55	2911	10708	374213	8.5%	0.65 [0.50 , 0.85]	+
**Subtotal (Wald[a])**		**6607**		**397027**	**49.7%**	**4.22 [0.51 , 34.69]**	
Total events:	1027		10926				
Test for overall effect: Z = 1.34 (P = 0.18)							
Heterogeneity: Tau^2 (DL[b]) = 6.81; Chi^2 = 813.33, df = 5 (P < 0.00001); I^2 = 99%							
**1.12.2 Anxiety**							
Xu 2008	3	47	13	444	7.6%	2.26 [0.62 , 8.24]	+
Abrams 2011	111	1784	933	21726	8.6%	1.48 [1.21 , 1.81]	+
Feng 2022	77	3288	10708	374213	8.6%	0.81 [0.65 , 1.02]	+
**Subtotal (Wald[a])**		**5119**		**396383**	**24.7%**	**1.20 [0.70 , 2.06]**	
Total events:	191		11654				
Test for overall effect: Z = 0.68 (P = 0.50)							
Heterogeneity: Tau^2 (DL[b]) = 0.16; Chi^2 = 15.85, df = 2 (P = 0.0004); I^2 = 87%							
**1.12.3 SMI**							
Abrams 2011	54	1569	990	21726	8.5%	0.75 [0.56 , 0.99]	+
Jorgensen 2018	101	1502	12739	210366	8.6%	1.12 [0.91 , 1.37]	+
Goldman 2023	40	2096	1014	52482	8.5%	0.99 [0.72 , 1.36]	+
**Subtotal (Wald[a])**		**5167**		**284574**	**25.6%**	**0.95 [0.74 , 1.22]**	
Total events:	195		14743				
Test for overall effect: Z = 0.42 (P = 0.68)							
Heterogeneity: Tau^2 (DL[b]) = 0.03; Chi^2 = 5.29, df = 2 (P = 0.07); I^2 = 62%							
**Total (Wald[a])**		**16893**		**1077984**	**100.0%**	**2.17 [0.77 , 6.16]**	
95% prediction interval						[0.05 , 88.46]	
Total events:	1413		37323				
Test for overall effect: Z = 1.46 (P = 0.14)							
Test for subgroup differences: Chi^2 = 2.42, df = 2 (P = 0.30), I^2 = 17.5%							
Heterogeneity: Tau^2 (DL[b]) = 3.29; Chi^2 = 1591.19, df = 11 (P < 0.00001); I^2 = 99%							

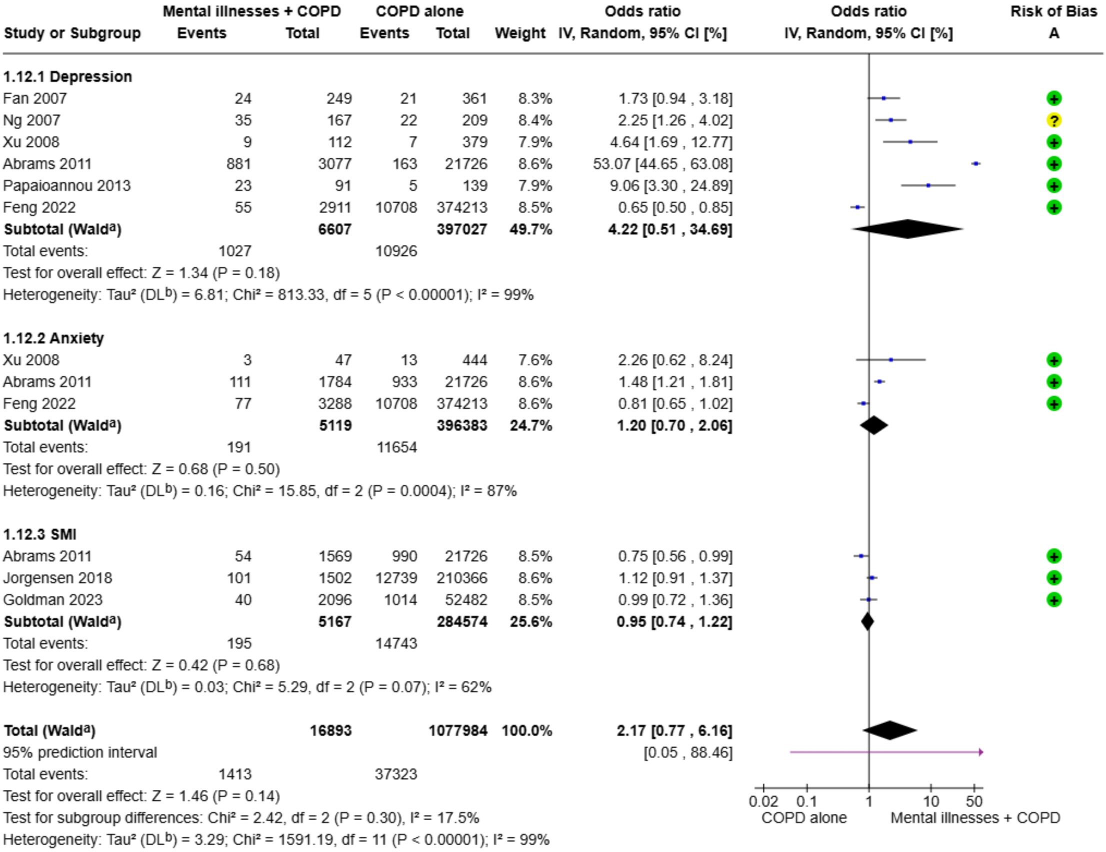

Footnotes
[a]CI calculated by Wald-type method.
[b]Tau^2 calculated by DerSimonian and Laird method.

Risk of bias legend
(A) Newcastle-ottwa scale

Supplement, Figure 9: Forest plot of studies presenting unadjusted data on mortality for COPD patients with and without mental illnesses.

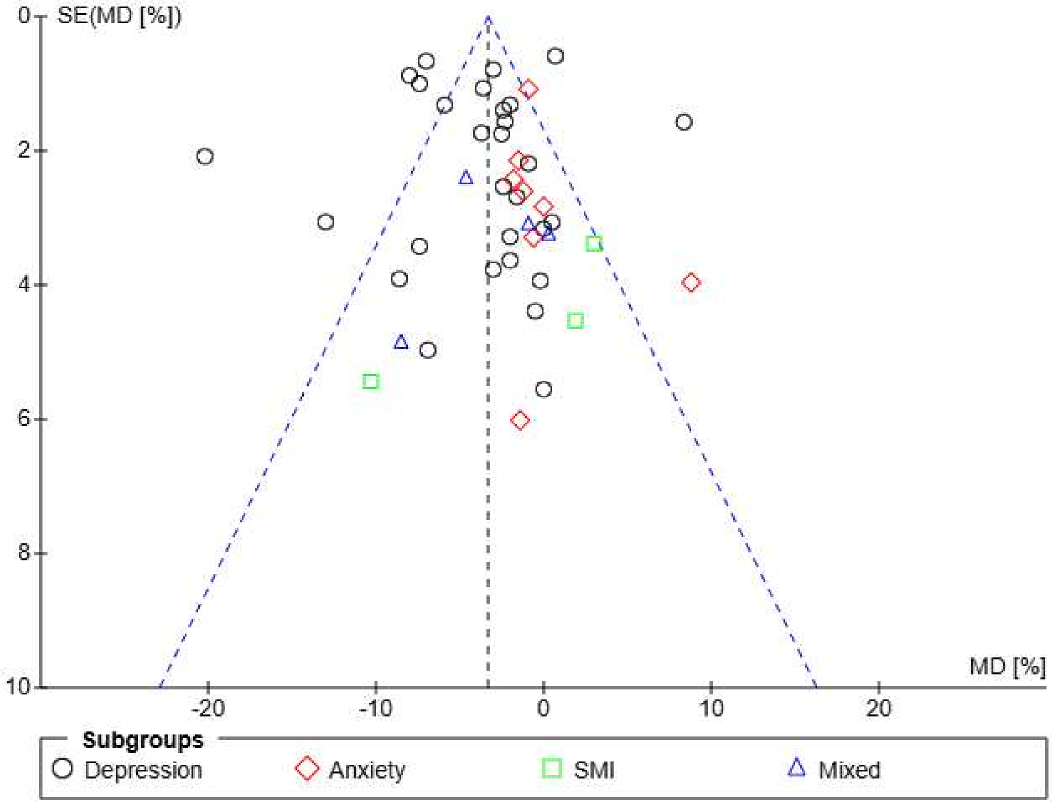

Supplement, Figure 10: Funnel plot of FEV1% by type of mental illness.

Alsaab S, *et al. BMJ Open Respir Res* 2026; 13:e003977. doi: 10.1136/bmjresp-2025-003977

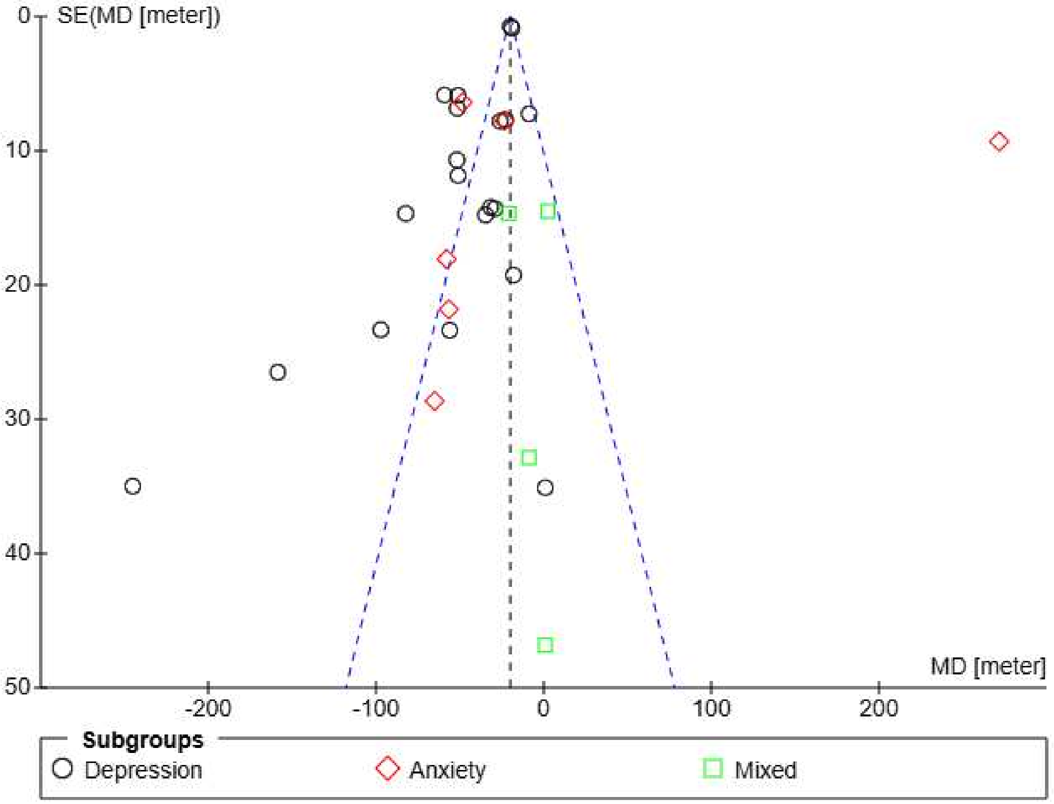

Supplement, Figure 11: Funnel plot of 6MWT by type of mental illness.

Alsaab S, *et al. BMJ Open Respir Res* 2026; 13:e003977. doi: 10.1136/bmjresp-2025-003977

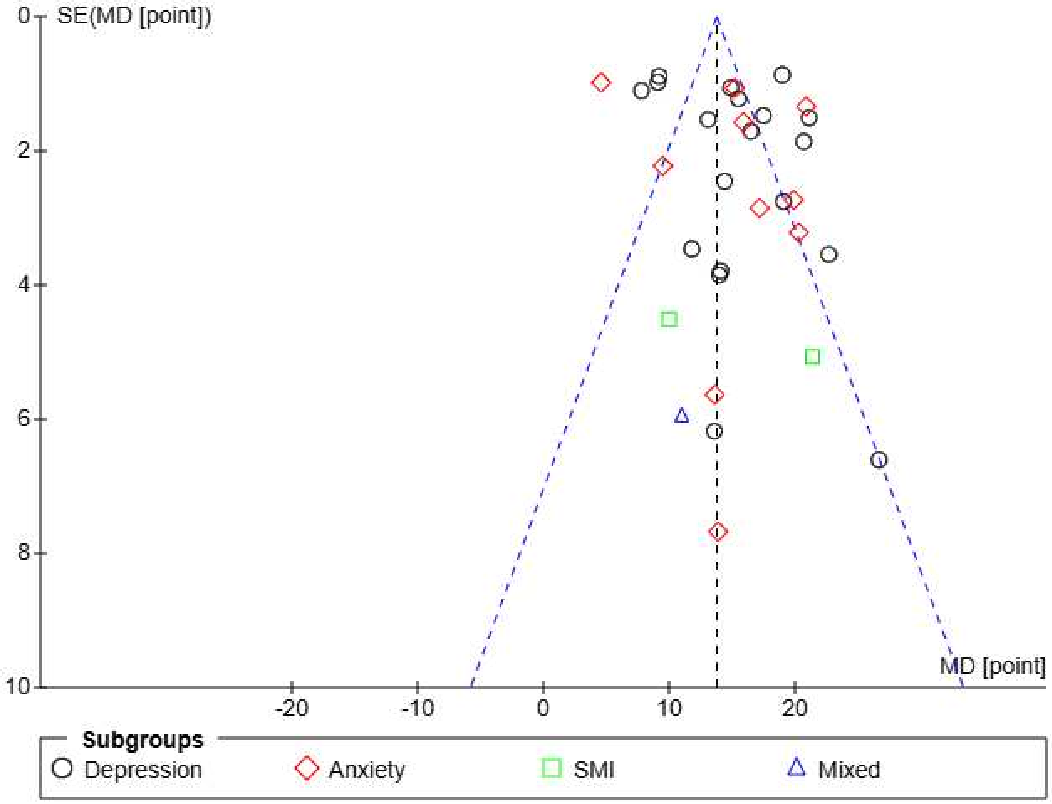

Supplement, Figure 12: Funnel plot of SGRQ by type of mental illness.

Alsaab S, *et al. BMJ Open Respir Res* 2026; 13:e003977. doi: 10.1136/bmjresp-2025-003977

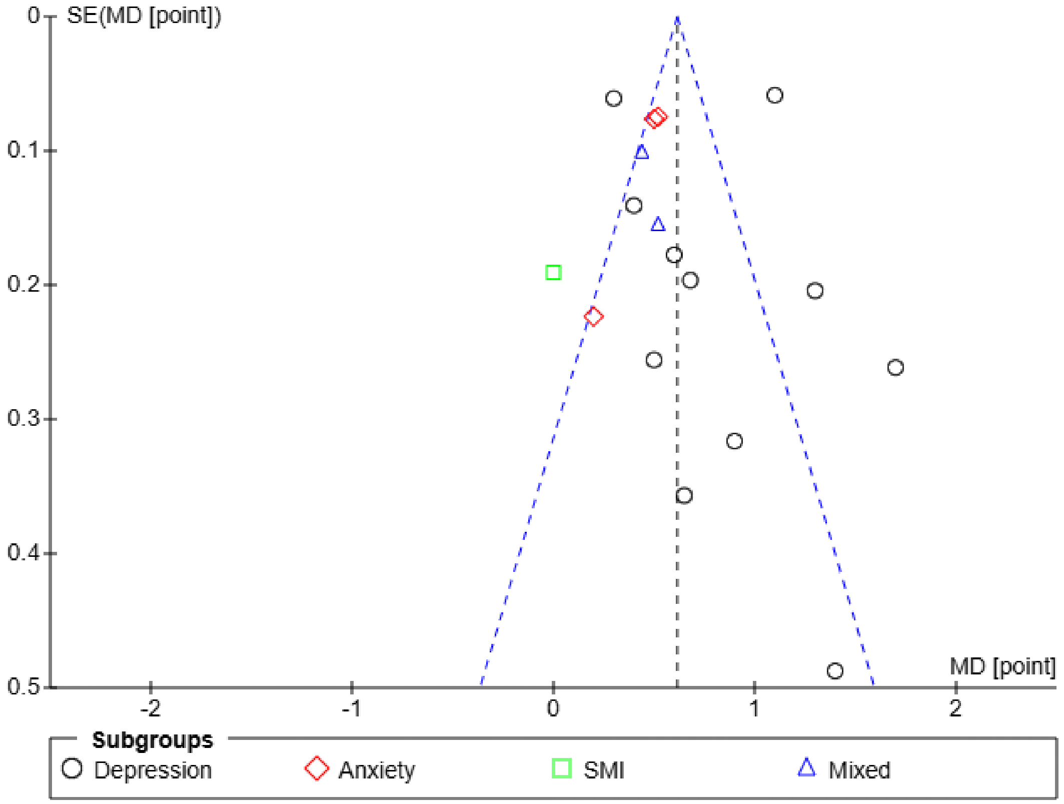

Supplement, Figure 13: Funnel plot of mMRC by type of mental illness.

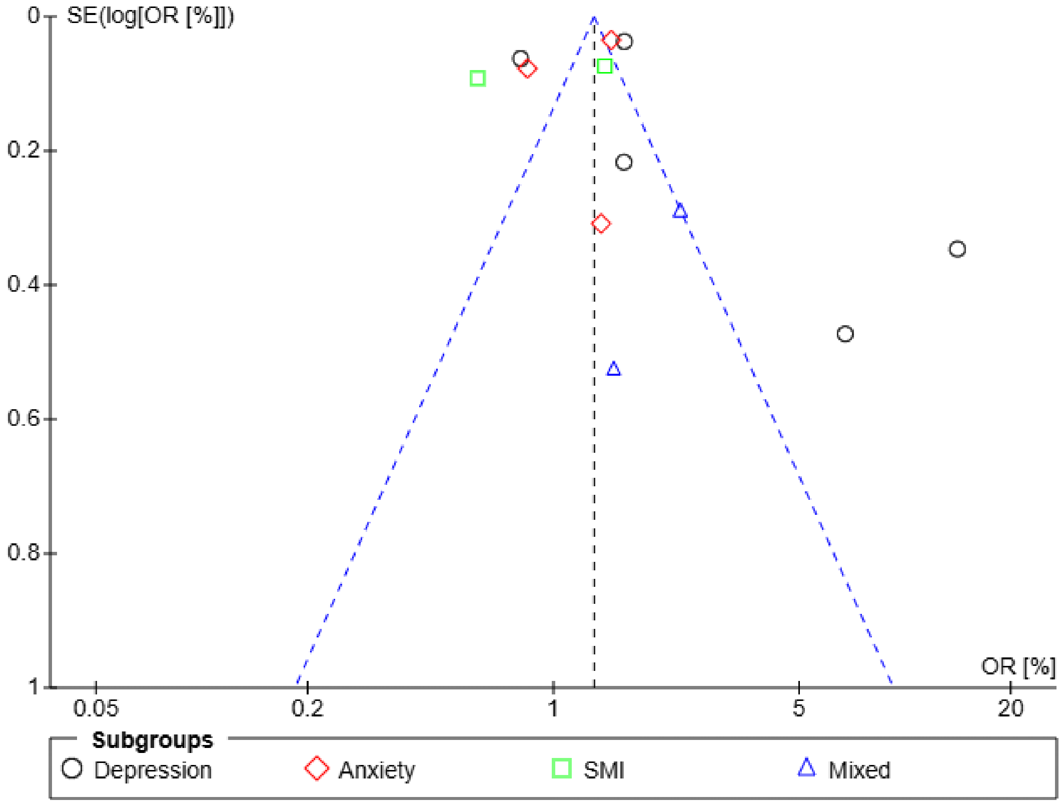

Supplement, Figure 14: Funnel plot of odds of hospitalisation by type of mental illness.

Alsaab S, *et al. BMJ Open Respir Res* 2026; 13:e003977. doi: 10.1136/bmjresp-2025-003977

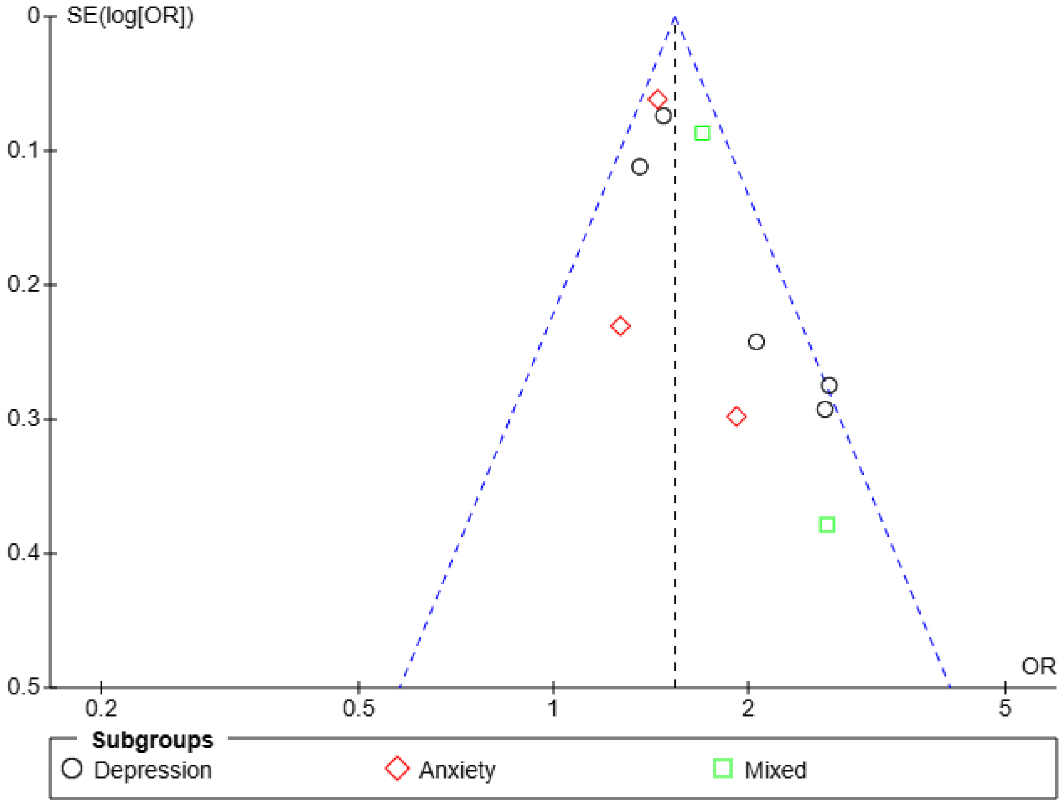

Supplement, Figure 15: Funnel plot of adjusted 1-year hospitalisation by type of mental illness.

Alsaab S, *et al. BMJ Open Respir Res* 2026; 13:e003977. doi: 10.1136/bmjresp-2025-003977

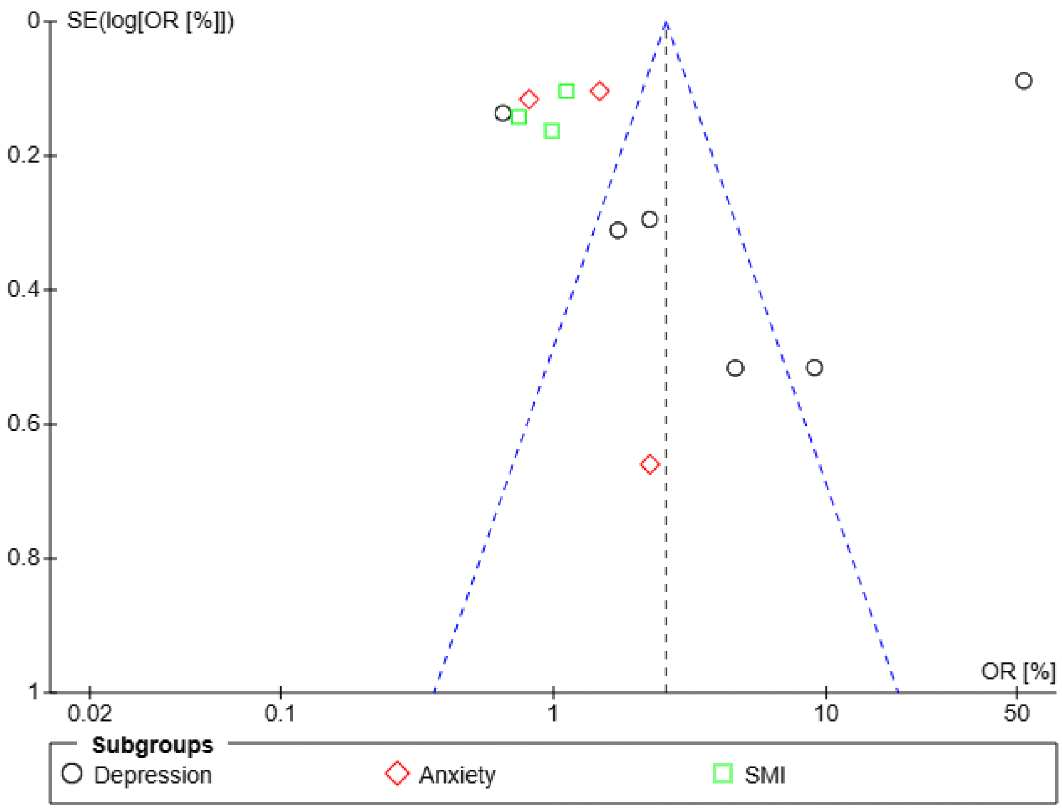

Supplement, Figure 16: Funnel plot of mortality by type of mental illness.

Alsaab S, *et al. BMJ Open Respir Res* 2026; 13:e003977. doi: 10.1136/bmjresp-2025-003977

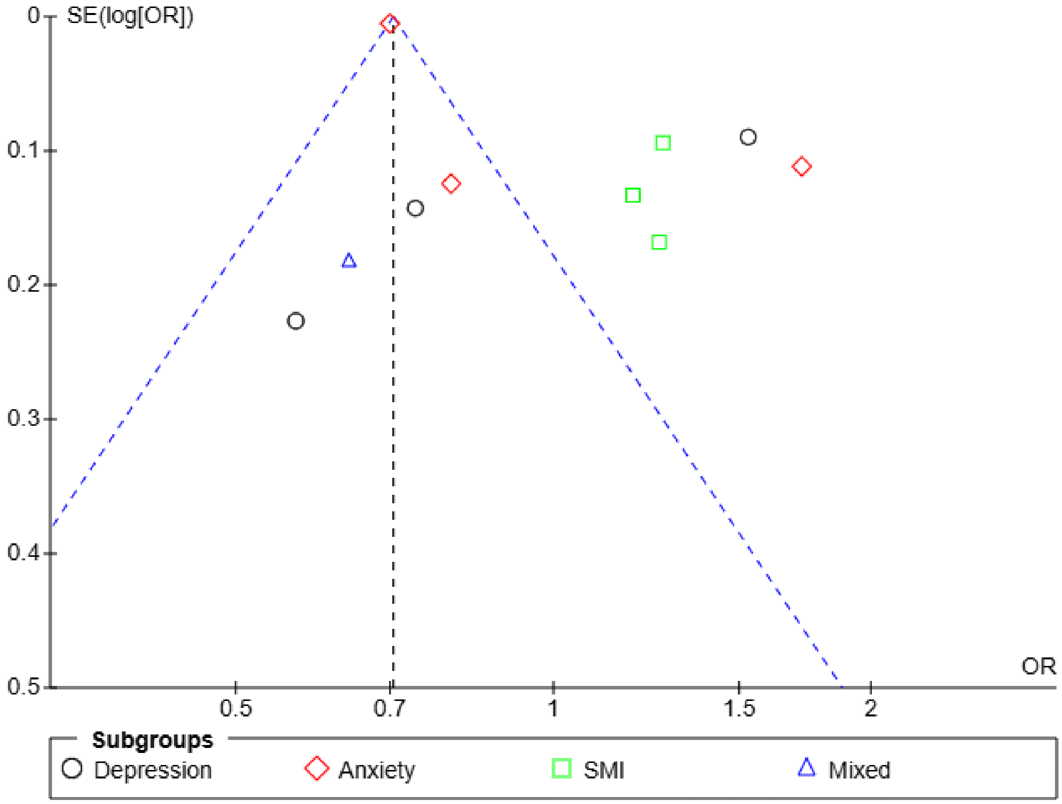

Supplement, Figure 17: Funnel plot of adjusted mortality by type of mental illness.

References:

Nayebirad, S., Mohamadi, A., Yousefi-Koma, H., Javadi, M., Farahmand, K., Atef-Yekta, R., Tamartash, Z., Jameie, M., Mohammadzadegan, A. M., & Kavosi, H. (2023). Association of anti-Ro52 autoantibody with interstitial lung disease in autoimmune diseases: a systematic review and meta-analysis. *BMJ Open Respiratory Research*, *10*(1). https://doi.org/10.1136/bmjresp-2023-002076

Zulkipli, M. S., Dahlui, M., Jamil, N., Peramalah, D., Wai, H. V. C., Bulgiba, A., & Rampal, S. (2018). The association between obesity and dengue severity among pediatric patients: A systematic review and meta-analysis. *PLoS Neglected Tropical Diseases*, *12*(2), e0006263. https://doi.org/10.1371/journal.pntd.0006263
